# Development of an online tool for linking behavior change techniques and mechanisms of action based on triangulation of findings from literature synthesis and expert consensus

**DOI:** 10.1093/tbm/ibaa050

**Published:** 2020-08-04

**Authors:** Marie Johnston, Rachel N Carey, Lauren E Connell Bohlen, Derek W Johnston, Alexander J Rothman, Marijn de Bruin, Michael P Kelly, Hilary Groarke, Susan Michie

**Affiliations:** 1 Aberdeen Health Psychology Group, Institute of Applied Health Sciences, College of Life Sciences and Medicine, University of Aberdeen, Foresterhill, Aberdeen, UK; 2 Department of Clinical, Educational and Health Psychology, University College London, London, UK; 3 Department of Kinesiology, University of Rhode Island, Kingston, RI, USA; 4 Department of Psychology, University of Minnesota, Minneapolis, MN, USA; 5 Institute of Public Health, University of Cambridge, Forvie Site, Cambridge, UK

**Keywords:** Online tool, Behavior change technique, Mechanism of action, Literature synthesis, Expert consensus, Triangulation

## Abstract

Researchers, practitioners, and policymakers develop interventions to change behavior based on their understanding of how behavior change techniques (BCTs) impact the determinants of behavior. A transparent, systematic, and accessible method of linking BCTs with the processes through which they change behavior (i.e., their mechanisms of action [MoAs]) would advance the understanding of intervention effects and improve theory and intervention development. The purpose of this study is to triangulate evidence for hypothesized BCT–MoA links obtained in two previous studies and present the results in an interactive, online tool. Two previous studies generated evidence on links between 56 BCTs and 26 MoAs based on their frequency in literature synthesis and on expert consensus. Concordance between the findings of the two studies was examined using multilevel modeling. Uncertainties and differences between the two studies were reconciled by 16 behavior change experts using consensus development methods. The resulting evidence was used to generate an online tool. The two studies showed concordance for 25 of the 26 MoAs and agreement for 37 links and for 460 “nonlinks.” A further 55 links were resolved by consensus (total of 92 [37 + 55] hypothesized BCT–MoA links). Full data on 1,456 possible links was incorporated into the online interactive Theory and Technique Tool (*https://theoryandtechniquetool.humanbehaviourchange.org/*). This triangulation of two distinct sources of evidence provides guidance on how BCTs may affect the mechanisms that change behavior and is available as a resource for behavior change intervention designers, researchers and theorists, supporting intervention design, research synthesis, and collaborative research.

**Implications**

**Practice:** Behavior change intervention designers can use the Theory and Technique Tool (TATT) to select behavior change techniques (BCTs) for inclusion based on expert evidence.

**Policy:** Policymakers involved in behavior change can use the TATT to access resources, request advice, and identify BCTs relevant to the changes they plan to implement.

**Research:** Future research can use the TATT to identify links between BCTs and theoretical constructs, download and upload evidence, and engage in discussion and collaborations.

## INTRODUCTION

Behavior change interventions are the basis for addressing many current global health challenges. Theoretical progress in behavioral science has identified key determinants of behavior and proposes that behavior change is elicited by interventions that alter these causal factors. These factors serve as the “mechanisms of action” (MoAs) mediating the effect of interventions on behavior change. In developing an intervention to change behavior, the researcher or practitioner typically has an explicit or implicit theory about the MoAs affecting the behavior and then seeks to incorporate techniques within their intervention that will engage these mechanisms and thereby the target behavior. There have been advances in the transparency and reporting of intervention methods, particularly in the specification of behavior change techniques (BCTs), the active ingredients of an intervention with the potential to change behavior, in the 93-item BCT Taxonomy (BCTTv1) [1–3]. Despite advances in specifying BCTs, investigators are faced with making decisions about which techniques to select. Further work is required to facilitate the choice of BCTs for targeting specific MoAs when designing interventions and to interpret the theoretical significance of BCTs that are part of effective interventions.

Evidence clarifying which BCTs might influence which MoAs is likely to prove useful in the field of behavioral medicine by suggesting the techniques to use in changing health-related behaviors. For example, a practitioner wishing to reduce dentists’ prescribing of antibiotic medications may seek very different intervention techniques if they have evidence that the dentists have the intention to reduce their prescribing compared with the situation where they lack the intention: in the latter case, the proposed MoA would be “intention,” and techniques would be selected that are thought to increase intention, whereas, in the former case, the MoA might be “memory,” “reinforcement,” or “social influences,” and the practitioner would select quite different techniques.

Several approaches to intervention development identify the need for guidance on links between BCTs and MoAs. In Intervention Mapping, a key step following identification of determinants is that the choice of behavior change methods to address these determinants and proposed links are provided [4]. Discussion of experimental medicine approaches proposes tests of pathways from intervention to behavior change through mediating MoAs [5]. The National Institutes of Health (NIH) Science of Behavior Change initiative aims to improve the measurement of MoAs and is testing the effect of changing MoAs [6,7]. These approaches require a methodology for linking MoAs to the BCTs that are likely to produce change in the MoA and, thereby, cause the target behavior to change. Given the large number of possible MoAs and BCTs, testing the mediating effect of all MoAs for each BCT is unrealistic and probably an inefficient use of resources. Here, we report studies designed to identify links between BCTs and MoAs that might be “best-bets” for implementation in research, practice, and policy. In two previous studies, we have identified and synthesized links made by authors in published literature [8] and by expert consensus [9], but further work is necessary to compare and reconcile the findings of these two studies before making the full evidence available to intervention designers, researchers, and policymakers in an online tool.

Triangulation of the findings of these two previous studies is necessary for two reasons. First, it is possible that the two studies provide conflicting or diverging evidence, resulting in ambiguous guidance for those wishing to implement the findings in designing interventions. By triangulating the findings, it is possible to identify the links supported in both studies, giving the user more confidence in applying the resulting evidence. Second, scientifically, triangulation of studies using different methods to tackle the same question gives greater confidence that the results obtained are not simply due to repetition of biases and limitations in design, methods, or analyses [10]. The literature synthesis presents evidence of past thinking by a wide range of authors engaged in the practical task of intervention development, and evaluation, whereas the expert consensus study represents current opinion by experts engaged in an explicit BCT–MoA linking task Furthermore, there were important methodological differences between the two studies that limit the extent of possible concordance. The literature study could have investigated any link that an author described, resulting in more BCTs than it was feasible to investigate in the consensus study.

Our approach was comprised of two steps. First, concordance between the findings of the two studies was investigated statistically to assess agreement and, then, to identify links described consistently by both methods. Second, inconclusive links from the concordance study were examined in a reconciliation study in which a new group of experts, referred to as “reconciliation experts,” were provided with the evidence from the two previous studies and asked to reconcile them. As suggested by Archibald [11], it was important that this reconciliation should be done by more than one or two researchers and should go beyond the original research team.

Since the results are intended as a resource for intervention development, evaluation, and synthesis, all findings were made accessible in an online tool that allows users to search for evidence of linkage (for each BCT–MoA combination, for each BCT, and for each MoA), share additional information about the links, and propose collaborative research into underinvestigated links.

## STUDY 1: CONCORDANCE STUDY COMPARING THE FINDINGS OF THE LITERATURE SYNTHESIS AND EXPERT CONSENSUS STUDIES

### Study design

The tables of BCT–MoA links produced by the two previous studies were compared in order to (a) estimate agreement between the findings of the two studies overall and for each MoA, (b) identify BCT–MoA links where there was agreement, that is, links that exceeded preset criteria in both the literature synthesis and expert consensus studies as either linked (frequent in literature; rated “definitely yes” by experts) or not linked (rare in literature; rated “definitely no” by experts), and c) identify links with inconclusive results from the two studies for further investigation in the reconciliation study.

Both the literature and consensus studies considered the same 26 MoAs. However, while the consensus study focused on linkages with a subset of 61 BCTs, the literature study had the potential to consider linkages with any of the 93 BCTs in BCTTv1. Also, the consensus study allowed experts to say explicitly that a potential BCT–MoA link definitely did *not* exist, whereas, in the literature study, it was only possible for a link to be absent from the 277 papers investigated. Thus, evidence of absent links in the latter study was ambiguous: a link might be absent because it could indicate the link is impossible or improbable or because it simply was not used in the 277 interventions.

### Methods

Full details of the methods used in the two previous studies are provided in [8] and [9]. Here, we provide essential details.

### Data from the literature synthesis study [8]

Peer-reviewed published behavior change intervention reports were identified using electronic searches, requests to experts, and citations of papers, which included some coding of either BCTs or theory. They were included if (a) they provided the description and/or evaluation of a behavior change intervention and (b) a BCT (not necessarily labeled as such by the authors) was explicitly linked to one or more MoA(s). BCTs in the reports were coded using BCTTv1 [1]. MoAs were coded if they (a) described a process through which behavior change could occur and (b) were clearly linked to a BCT. Authors’ descriptions of MoAs were categorized into the 26 MoAs comprised of (a) the 14 theoretical domains described in the Theoretical Domains Framework (which are starred in Table 2) [12,13] *and* (b) the 12 most frequently occurring MoAs (which did not overlap with the theoretical domains) identified in a systematic review of 83 behavior change theories [14].

A total of 2,636 BCT–MoA links were made by authors, including 70 BCTs and 25 MoAs, identified from 277 articles (mean number of links per article = 9.56, standard deviation *=* 13.80). For each possible link, a one-tailed exact binomial test was conducted comparing the observed to the expected frequency of occurrence for each link, computing an expected value as the frequency that might be observed if BCTs were randomly linked to MoAs (see details in [8], p. 696–697). The *p* values from these tests give an indication of the likelihood of a link and are the data for the current concordance study. Scores range from 0 to 1 and lower values indicate links occurring *with greater relative frequency*: in this paper, these scores are labeled *p*+ to differentiate them from tests of statistical significance. The criterion for a link was set at a probability, *p*+, of ≤.05; this criterion value should not be interpreted as a statistical test but simply as a threshold probability value for identifying links of higher relative frequency. Full details of the exact probabilities for each possible link are presented in the heat maps in [8] and in the online Theory and Technique Tool (TATT; *https://theoryandtechniquetool.humanbehaviourchange.org/*), allowing readers to examine the relative frequency of links in more detail. Eighty-seven BCT–MoA combinations met the criterion for a link.

### Data from the expert consensus study [9]

Sixty-one BCTs and 26 MoAs were included in the study based on the following criteria: (a) BCTs had to be commonly used within the intervention literature; therefore, we selected only those BCTs identified more than twice (*n* = 61) in a set of 40 systematically identified and coded intervention descriptions covering a range of different behaviors [15]; (b) MoAs were restricted to the same 26 MoAs as in the literature synthesis study. In order to ensure that the task was manageable for the 100 experts, we divided the BCTs into five groups and allocated either 13 or 14 BCTs × 26 MoAs (i.e., 338 or 364 possible links) for judgment by each group of 21 experts.

Experts individually rated each link in an online task, then participated in an online, asynchronous discussion of the ratings of their group, and finally completed a final set of individual ratings using responses: “Definitely Yes,” “Definitely No,” “Uncertain,” or “Possibly.” These final ratings provide the data for the concordance study. Two scores are used for each of the 1,586 links: Proportion of YES = the proportion of experts answering “Definitely Yes”; and Proportion of NO = the proportion of experts answering “Definitely No.” Scores ranged from 0 to 1 and higher scores indicate a higher proportion of agreeing on links or “nonlinks” (answers of “Uncertain” or “Possibly” were excluded.)

### Analyses

Because the two studies shared 56 BCTs and all 26 MoAs, the concordance in the probability of a link could be assessed for 1,456 (56 BCTs × 26 MoAs) possible links. Concordance was examined using two approaches: (a) multilevel modeling (MLM) and (b) comparison of the matrices of BCT–MoA links.

#### Multilevel modeling

The dependent variable was the proportion of experts either asserting that there was a link (definitely “yes”) for a given BCT/MoA combination or, separately in a second model, that there was no link (definitely “no”). The predictor was the relative frequency of each link as indicated by the exact *p* value, *p*+, in the literature synthesis. The decision to treat expert consensus as the independent and literature synthesis as the dependent variables for the MLM was based on (a) the better distribution of data for the consensus study as there were fewer tied results and (b) the literature synthesis preceded the consensus task.

In a two-level analysis using MLwiN 3.01, BCTs were nested within MoAs. The intercept was allowed to vary randomly at both levels; the probability of a link from the literature synthesis varied at the MoA level. These analyses can show if there is a relationship between the links established by the literature synthesis and the expert consensus across all MoAs and if that relationship varies by MoA.

In many cases, the literature synthesis identified a BCT linked to at least one MoA but no links to other MoAs. Such absences of a link to an MoA obtained a *p*+ value of 1. For example, BCT 2.6 “Biofeedback” was linked in the literature study to MoA “Skills” but not to MoA “Knowledge” (see Supplementary File 2) and, therefore, had a *p*+ value of 1 for MoA “Knowledge.” Since the other links were positively established, that is, by their presence rather than absence, they have greater credence and so the data were analyzed with and without the cases where *p*+ = 1. The variance explained by the various models was calculated using the procedures recently described by Nakagawa and Schielzeth [16] and Johnson [17].

#### Comparison of matrices of BCT–MoA links

In both literature synthesis and expert consensus studies, a criterion was set for establishing a link. There is no standard formula for setting such criteria. For the literature study, the preset criterion, *p*+, was set comparable to statistical significance conventions as a *p* value for the binomial test of ≤.05. For the expert study, the criterion was preset at agreement by 80% or more of respondents, falling between conventions in Delphi studies at approximately 67% (such as [18]) and the higher cutoff of 90% frequently used in studies of sensitivity and specificity of classifications.

We compared the findings of the two studies and classified the links into one of the following categories: (a) *Link*: evidence of link across both studies (i.e., link was found in both literature synthesis and expert consensus), (b) *No Link*: no evidence of link in literature synthesis, evidence of “no link” in expert consensus (i.e., link was absent in literature synthesis study and experts in consensus study agreed that there was no link), (c) *No evidence*: no evidence of link in literature synthesis and no strong evidence in expert consensus (i.e., link was absent in literature synthesis study and less than 80% of experts in consensus study agreed whether or not there was a link), (d) *Inconclusive*: evidence of link in literature synthesis but evidence of “no link” in expert consensus or no evidence of link in literature synthesis but evidence of link in expert consensus *or* some below-criterion level of evidence in either the literature (.05 < *p*+ <.10) and/or expert consensus (e.g., link agreed by 70%–80% of experts). The first three of these categories were categorized as “concordance” between the two studies (agreement of presence of “link,” agreement of “no link,” or agreement that evidence was lacking). Links from the fourth, inconclusive, category were brought forward to be considered by the reconciliation experts.

## RESULTS

**Fig 1:**
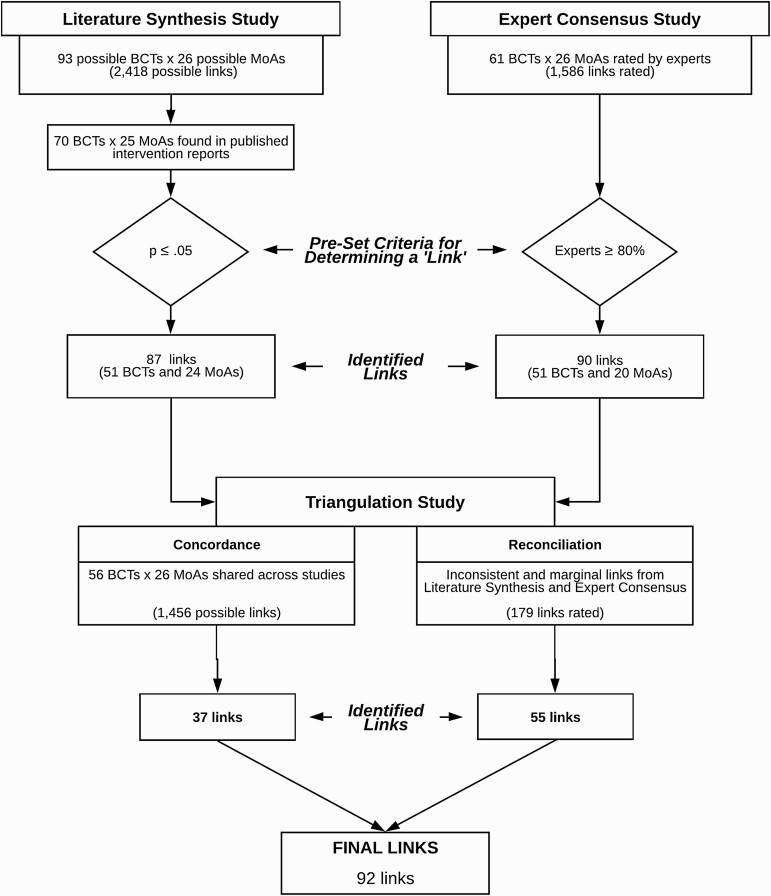
**Flowchart** showing stages in the research and the links between behavior change techniques and mechanisms of action at each stage.

The flowchart in Fig. 1 summarizes the stages of the research and the data at each stage.

### Results of MLM to assess agreement between the findings of the literature synthesis and expert consensus studies

**Fig 2:**
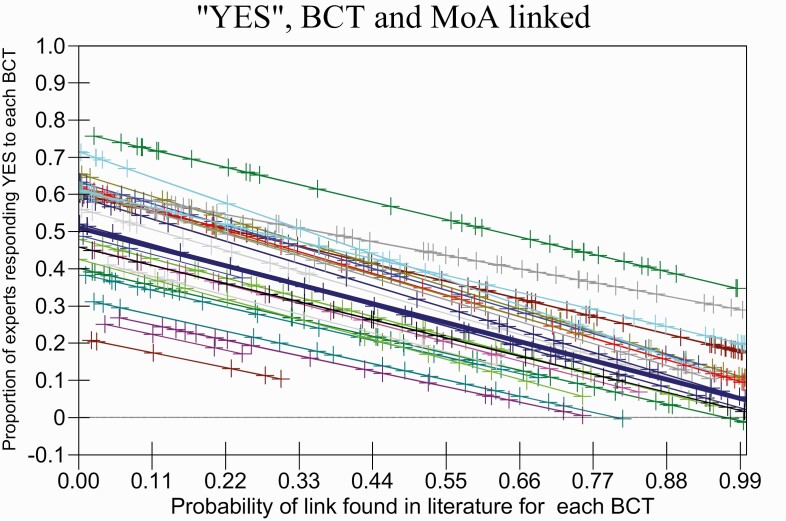
**Relation between findings from the literature synthesis and expert consensus studies** when links between behavior change techniques and mechanisms of action (MoAs) have been proposed. Each line represents the prediction from the multilevel model. The thick line is the overall regression line. Other lines represent each MoA. The dots indicate the predicted values for actual data points. The occasions when no link was proposed in the literature synthesis study (coded as *p*+ = 1) have been omitted. The negative slope indicates concordant relationships between the two studies with the steepness of the slope indicating the strength of the relationship.

Figure 2 shows the *p*+ values from the literature synthesis study plotted against the proportion of “definitely YES” responses in the expert consensus study; each line represents one MoA. Lower *p*+ values, indicating links with greater relative frequency, are associated with a higher proportion of “YES” responses.

The regression lines and scatterplots show the proportion of experts asserting a link in the expert consensus study against the probability of a link in the literature synthesis study for each BCT linked to each MoA. The negative slope indicates concordant relationships between the two studies with the steepness of the slope indicating the strength of the relationship.

**Fig 3:**
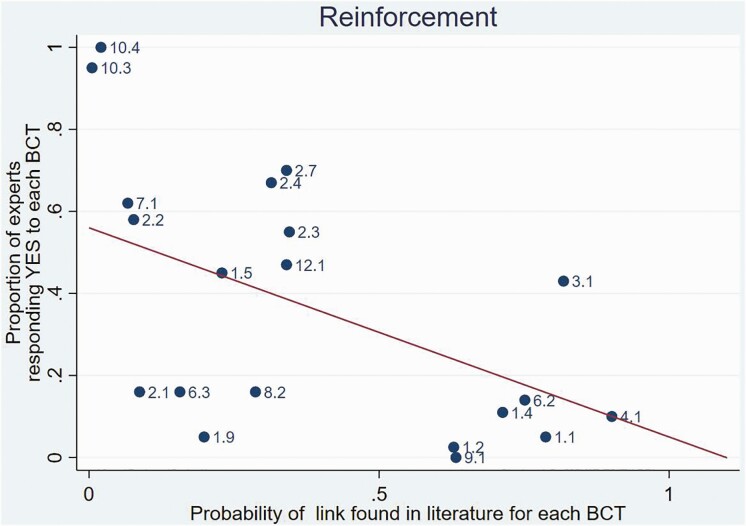
**Illustrative example** of relation between findings from the literature synthesis and expert consensus studies for mechanisms of action “Reinforcement.” Each dot represents one behavior change technique (BCT; with BCTTv1 label). The line represents the prediction from the multilevel model omitting BCTs for which *p* = 1 in the literature synthesis study (but the expert consensus values for such BCTs are shown). The BCTs labeled are: BCT 10.3 = nonspecific reward; BCT 10.4 = social reward; and BCT 4.1 = instruction on how to perform the behavior.

In order to illustrate the observed relation between the findings in the two studies, Fig. 3 provides a detailed illustration for a single MoA “Reinforcement,” showing how BCTs are linked (or not linked) to the MoA in the two studies and presenting the two BCTs with greatest agreement and the one with least agreement, with BCTs labeled as in BCTTv1.

In the Reinforcement example, a strong link is found across both studies for nonspecific reward (BCT 10.3) and social reward (BCT 10.4; i.e., a low *p*+ value in the literature and a high proportion of experts responding “YES”), whereas “no link” was found in both studies for instruction on how to perform the behavior (BCT 4.1). In addition to scientific evidence of triangulation, the results can provide recommendations to users who are trying to decide which BCT to use to engage a particular MoA. Thus, a user who hopes to change behavior via the MoA of Reinforcement might choose to use BCT 10.3 or 10.4 but avoid BCT 4.1. Detailed scatterplots for each of the 25 MoAs (not for “norms” as this was not found in the literature study), showing which BCTs are linked to which MoAs in the two studies, can be accessed in Supplementary File 2.

Further details about the multilevel models are reported in Table 1 and details of unstandardized regression intercepts and slopes for each MoA are shown in Table 2. The results are broadly similar whether *p*+ values of 1 (which occur when a BCT is not linked to the target MoA) are included or removed. Both sets of results are presented in Table 1. Table 2 presents the results with *p*+ values of 1 removed but both sets of results are presented in Supplementary File 1: S7. The total variance explained by the “YES” model was 42.8%. The comparable value for the “NO” responses was 45.0%. Larger intercepts for “YES” responses indicate greater agreement across studies that a link exists and the slopes indicate the relationship between the studies for each MoA. Apart from the MoA “norms,” which was not found in the literature synthesis study, each MoA shows negative slopes for “YES” responses and positive slopes for “NO” responses indicating moderate concordance between the results of the two studies for virtually all MoAs. There was little difference in slopes across MoAs for either the “YES” or “NO” responses.

**Table 1:** Multilevel model predicting judgments of expert consensus from the literature synthesis: estimated beta weights (standard error) for fixed effects and variances (standard error) for random effects

	“YES” expert consensus		“NO” expert consensus	
	All data	*p*± = 1 omitted	All data	*p*± = 1 omitted
Fixed effects				
Intercept	0.474(.037)***	0.510 (.036)***	0.295(.030)***	0.270(.029)***
Literature	−0.325(.031)***	−0.464(.042)***	0.327(.24)***	0.439(.035)***
Random effects				
Level 2: mechanism of action				
Intercept	0.029(.01)***	0.025(.009)*	0.017(.007)*	0.014(.006)
Literature	0.016 (.007)*	0.014 (.011)	0.006(.004)	0.005(.007)
Level 1: behavior change techniques				
Intercept	0.045(.002)***	0.059(.004)***	0.056(.002)***	0.058(.003)***

*p < .01, **p < .01, ***p < .001.

**Table 2:** Predicting expert consensus from literature study for each mechanism of action (MoA) for “YES” and “NO” responses (multilevel modeling: intercept [Int] and slope [Slp]; without *p*+ = 1 behavior change techniques)

	“YES”			“NO”		
MoA	*n*	Int	Slp	*n*	Int	Slp
Knowledge^a^	29	.52	−.50	29	.26	.43
Skills^a^	36	.40	−.41	36	.39	.54
Social/professional role and identity^a^	19	.32	−.40	19	.39	.52
Beliefs about capabilities^a^	51	.60	−.43	51	.14	.38
Optimism^a^	19	.29	−.38	19	.36	.49
Beliefs about consequences^a^	37	.66	−.55	37	.20	.37
Reinforcement^a^	20	.56	−.51	20	.26	.44
Intentions^a^	42	.62	−.33	42	.08	.34
Goals^a^	28	.63	−.53	28	.18	.37
Memory, attention, and decision processes^a^	28	.48	−.47	28	.27	.43
Environmental context and resources^a^	22	.71	−.62	22	.22	.37
Social influences^a^	28	.62	−.53	28	.26	.40
Emotion^a^	20	.46	−.47	20	.29	.43
Behavioral regulation^a^	36	.61	−.41	33	.15	.38
Norms	−	−	−	−	−	−
Subjective norms	25	.46	−.44	25	.30	.46
Attitude toward the behavior	27	.59	−.54	27	.21	.37
Motivation	36	.77	−.43	36	.03	.29
Self-image	15	.39	−.41	15	.30	.47
Needs	6	.21	−.36	6	.55	.66
Values	4	.27	−.38	4	.42	.55
Feedback processes	12	.61	−.52	12	.24	.42
Social learning/imitation	10	.43	−.46	10	.41	.54
Behavioral cueing	21	.61	−.54	21	.26	.42
General attitudes/beliefs	3	.49	−.47	3	.27	.43
Perceived susceptibility/vulnerability	11	.43	−.49	11	.38	.50

No data for “Norms.”

^a^MoA from Theoretical Domains Framework.

### Results of comparison of matrices of BCT–MoA links to identify links and nonlinks found in both literature consensus and expert consensus studies

Links exceeding preset criteria for both studies were examined: 37 BCT–MoA “links” reached the criterion for a link in both the literature synthesis and expert consensus studies, covering 28 BCTs and 18 MoAs (see Table 3).

**Table 3:** Links agreed to be present in the comparison of matrices from the two previous studies (i.e., links present in both literature synthesis and expert consensus studies)

		Literature synthesis study		Expert consensus study
Behavior change technique	Mechanism of action	Frequency (number of papers)	*p+* value	Proportion experts (definitely yes)
1.2 Problem solving	Beliefs about capabilities	65	.008	.95
1.3 Goal setting (outcome)	Goals	4	.003	1.00
1.6 Discrepancy between current behavior and goal	Goals	3	.001	.81
1.7 Review outcome goals	Goals	2	.012	.89
2.7 Feedback on outcomes of behavior	Feedback processes	2	.027	.80
3.1 Social support (unspecified)	Social influences	34	<.001	.87
3.2 Social support (practical)	Environmental context and resources	3	.026	.90
	Social influences	4	.023	.81
4.1 Instruction on how to perform the behavior	Skills	20	.024	.86
5.1 Information about health consequences	Knowledge	18	<.001	.89
	Beliefs about consequences	26	<.001	.95
	Perceived susceptibility/vulnerability	10	<.001	.84
5.3 Information about social and environmental consequences	Beliefs about consequences	20	<.001	.95
5.6 Information about emotional consequences	Beliefs about consequences	6	.005	.90
6.1 Demonstration of the behavior	Social learning/imitation	3	.044	.84
6.2 Social comparison	Social influences	9	.043	1.00
	Subjective norms	31	<.001	.81
6.3 Information about others’ approval	Subjective norms	13	<.001	.89
7.1 Prompts/cues	Behavioral cueing	6	.002	1.00
	Environmental context and resources	5	.036	.90
8.1 Behavioral practice/rehearsal	Skills	24	<.001	.95
	Beliefs about capabilities	47	.013	.86
8.3 Habit formation	Behavioral cueing	3	.001	.85
8.7 Graded tasks	Beliefs about capabilities	28	<.001	.90
9.2 Pros and cons	Beliefs about consequences	12	<.001	.90
	Attitude toward the behavior	9	<.001	.81
	Motivation	5	.023	.86
9.3 Comparative imagining of future outcomes	Beliefs about consequences	3	.017	1.00
10.3 Nonspecific reward	Reinforcement	2	.005	.95
10.4 Social reward	Reinforcement	3	.020	1.00
12.1 Restructuring the physical environment	Environmental context/resources	9	<.001	.95
	Behavioral Cueing	3	.020	.89
12.2 Restructuring the social environment	Environmental context/resources	3	.004	.95
12.5 Adding objects to the environment	Environmental context/resources	8	<.001	.95
13.1 Identification of self as role model	Self-image	2	.011	.90
13.2 Framing/reframing	Attitude toward the behavior	7	.014	.81
15.1 Verbal persuasion about capability	Beliefs about capabilities	27	<.001	1.00

For 460 links (61 BCTs and 22 MoAs), there was also concordant evidence of “no link”; that is, absence of evidence in the literature and agreement between the experts that these BCTs do not act on those MoAs (see Supplementary File 1; Supplementary Table S5). Thus, there was concordance between the studies for a total of 497 (31.3%) of the total 1,586 possible links at this stage.

Inconclusive links (*N* = 179) were brought to the reconciliation study: evidence of a link in literature synthesis but “definitely no” link in expert consensus (*n* = 3); evidence of a link in literature synthesis and below-criterion level of evidence or link not included in expert consensus (*n* = 45); no evidence of a link in literature synthesis but “definitely” a link in expert consensus (*n* = 53); below-criterion level of evidence in either the literature synthesis (.05 ≤ *p*+ ≤.1) *or* the expert consensus study (70%–79% of experts answered “definitely yes” [*n* = 78]; full details in Supplementary File 1; Supplementary Tables S1–S4). Due to error, 11 inconclusive links were omitted from the reconciliation study and 6 were incorrectly included in the reconciliation study. These 17 links were removed from analyses.

There was insufficient evidence in both studies (i.e., link did not reach the threshold of .05 > *p* < .10 in literature synthesis study and <70% experts agreed the link was definitely present or absent) for 904 (56.9%) “absence of evidence” links.

## STUDY 2: RECONCILIATION OF THE FINDINGS OF THE LITERATURE AND EXPERT CONSENSUS STUDIES

### Study design

Inconclusive findings from the concordance study were examined by a new group of experts in a consensus exercise over three rounds in order to identify links that could be reconciled.

### Methods

#### Participants: reconciliation experts

Sixteen experts who design, evaluate, and/or synthesize evidence about theory-based behavior change interventions were selected from the database of experts for the original expert consensus exercise (and who were not participants in the previous expert consensus study). Additional experts were selected based on the recommendations of the project’s international advisory board. Experts were invited to participate based on a self-assessment questionnaire on their objective experience of publishing papers and conducting systematic reviews to assure expertise and a breadth of background (see [11]). Participants were based in the UK (*n* = 11), USA (*n* = 1), Canada (*n* = 2), Australia (*n* = 1), and Russia (*n* = 1).

#### Materials

One hundred seventy-nine inconclusive links were identified in the concordance study.

### Procedure

Expert consensus methodology was used to examine the 179 BCT–MoA links that were identified as inconclusive based on the comparison of the two prior studies. The task consisted of three rounds.

#### Round 1

Prior to the initial round, experts were emailed detailed guidelines for the study, including the sources of the data to be presented (see Supplementary File 4). In the initial rating round, experts were presented with the inconclusive BCT–MoA links from the concordance study. These 179 potential (inconclusive) links were presented in random order, alongside the information from the two sources of evidence, and experts were asked to rate each possible link as “definitely yes,” “definitely no,” or “uncertain/don’t know” taking into account the evidence provided. Experts had access to definitions of all BCTs and MoAs at all times. After completing Round 1, each expert received an email with a personalized statistical summary of the results of Round 1. This included frequency distributions, which were depicted alongside their own responses for each BCT × MoA link.

#### Round 2

During Round 2, experts took part in an online, anonymous, asynchronous discussion hosted via Loomio, a digital discussion platform, which they could access over a 2 week period. The purpose of this round was to facilitate discussions between experts on the 25 links for which there remained high uncertainty and/or disagreement. Experts were advised to focus the discussion on links for which they remained uncertain or where their views differed from those of other experts. A moderator from the research team periodically summarized the discussion and raised issues for further discussion.

#### Round 3

During Round 3, experts had access to their personalized statistical summaries from the Round 1 ratings, the original information comparing the two sources of evidence, and were provided with transcripts of the Round 2 discussions. The detailed information from the previous rounds allowed experts to reevaluate their original ratings for each link in light of the thoughts and ratings of the other experts. As in Round 1, the potential links were presented in random order and experts judged whether each BCT–MoA pair was linked by rating “definitely yes,” “definitely no,” or “uncertain/don’t know.” A link was judged to be resolved as a link if 80% or more of the reconciliation experts rated it “definitely yes” or as a “no link” if 80% or more rated it “definitely no.” The data from all studies were made available for each of the 1,456 BCT–MoA links in an interactive format.

### Results

The 179 links brought forward to be considered by the reconciliation experts were those for which there was inconclusive evidence across the literature synthesis and expert consensus studies. Reconciliation experts reached the criterion for agreement for 60/179 (33.52%) BCT–MoA links: For five possible links, experts agreed there was “no link,” that is, ≥80% rated it “definitely no” link (see Supplementary File 1; Supplementary Table S6) and, for 55, agreed there was a link, that is, ≥80% rated it “definitely yes” there was a link (see Table 4). The remaining 119 possible links continued to be inconclusive as there was insufficient evidence to indicate either a “link” or “no link.”

**Table 4:** Links agreed to be present in reconciliation study (i.e., 80% or more of experts agreed there is a link)

		Literature synthesis study			Expert consensus study			Reconciliation study		
Behavior change technique	Mechanism of action	Frequency (number of papers)	*p+* value	% Experts (yes)	% Experts (possibly)	% Experts (don’t know)	% Experts (no)	% Experts (yes)	% Experts (don’t know)	% Experts (no)
8.2 Behavior substitution	Behavioral regulation	5	0.016	79	5	0	16	93.75	6.25	0
5.1 Information about health consequences	Attitude toward the behavior	19	<0.001	68	21	0	11	81.25	18.75	0
5.3 Information about social and environmental consequences	Attitude toward the behavior	16	<0.001	76	5	9.5	9.5	93.75	6.25	0
5.3 Information about social and environmental consequences	Knowledge	13	0.002	67	24	0	9	81.25	18.75	0
2.3 Self-monitoring of behavior	Behavioral regulation	18	<0.001	70	15	0	15	93.75	6.25	0
9.1 Credible source	General attitudes/beliefs	2	0.007	68	26	0	6	87.5	12.5	0
15.4 Self-talk	Beliefs about capabilities	8	0.054	42	47	0	11	81.25	18.75	0
15.3 Focus on past success	Beliefs about capabilities	23	<0.001	N/A	N/A	N/A	N/A	100	0	0
1.2 Problem solving	Behavioral regulation	13	0.13	100	0	0	0	100	0	0
1.6 Discrepancy between current behavior and goal	Feedback processes	1	0.08	100	0	0	0	100	0	0
10.8 incentive (Outcome)	Motivation	N/A	N/A	100	0	0	0	87.5	12.5	0
1.1 Goal setting (behavior)	Intention	17	0.33	95	5	0	0	87.5	12.5	0
1.1 Goal setting (behavior)	Goals	4	0.23	95	0	5	0	87.5	12.5	0
5.1 Information about health consequences	Intention	28	0.004	63	26	0	11	81.25	18.75	0
2.2 Feedback on behavior	Feedback processes	3	0.07	95	5	0	0	100	0	0
7.1 Prompts/cues	Memory, attention, and decision processes	8	<0.001	76	24	0	0	93.75	6.25	0
11.3 Conserving mental resources	Memory, attention, and decision processes	1	0.07	100	0	0	0	81.25	18.75	0
10.1 Material incentive (behavior)	Reinforcement	0	1	95	5	0	0	93.75	6.25	0
10.4 Social reward	Social influences	2	0.56	95	5	0	0	81.25	18.75	0
11.2 Reduce negative emotions	Emotion	1	0.21	95	0	5	0	87.5	12.5	0
12.3 Avoidance/reducing exposure to cues	Environmental context and resources	1	0.22	95	5	0	0	93.75	6.25	0
12.3 Avoidance/reducing exposure to cues	Behavioral cueing	0	1	95	5	0	0	100	0	0
12.5 Adding objects to the environment	Behavioral cueing	2	0.12	95	5	0	0	93.75	6.25	0
14.10 Remove punishment	Reinforcement	N/A	N/A	95	5	0	0	93.75	6.25	0
1.5 Review behavior goals	Goals	2	0.064	90	0	0	10	87.5	12.5	0
6.2 Social comparison	Norms	0	1	90	5	0	5	81.25	18.75	0
6.2 Social comparison	Feedback processes	2	0.21	90	5	0	5	100	0	0
11.2 Reduce negative emotions	Behavioral regulation	0	1	90	5	5	0	81.25	18.75	0
7.5 Remove aversive stimulus	Environmental context and resources	N/A	N/A	90	5	5	0	87.5	12.5	0
10.8 Incentive (outcome)	Reinforcement	N/A	N/A	90	0	5	5	87.5	12.5	0
10.10 Reward (outcome)	Reinforcement	N/A	N/A	90	5	5	0	100	0	0
10.10 Reward (outcome)	Motivation	N/A	N/A	90	10	0	0	81.25	18.75	0
6.3 Information about others’ approval	Norms	0	1	90	5	5	0	93.75	6.25	0
10.2 Material reward (behavior)	Reinforcement	0	1	90	0	5	5	87.5	12.5	0
4.2 Information about antecedents	Knowledge	3	0.051	86	14	0	0	93.75	6.25	0
4.1 Instruction on how to perform the behavior	Knowledge	17	0.013	74	18	1	7	81.25		0
5.2 Salience of consequences	Beliefs about consequences	2	0.43	85	10	0	5	81.25	18.75	0
6.3 Information about others’ approval	Social influences	4	0.20	84	16	0	0	87.5	12.5	0
8.7 Graded tasks	Skills	4	0.45	81	14	0	5	81.25	18.75	0
2.3 Self-monitoring of behavior	Feedback processes	2	0.17	80	15	0	5	81.25	18.75	0
2.6 Biofeedback	Knowledge	0	1	79	16	0	5	81.25	12.5	6.25
6.1 Demonstration of the behavior	Beliefs about capabilities	60	0.003	58	37	5	0	87.5		0
4.1 Instruction on how to perform the behavior	Beliefs about capabilities	62	0.08	79	17	0	4	87.5	12.5	0
9.1 Credible source	Attitude toward the behavior	7	0.09	79	21	0	0	100	0	0
10.1 Material incentive (behavior)	Beliefs about consequences	0	1	79	10.5	0	10.5	87.5	12.5	0
10.6 Nonspecific incentive	Reinforcement	N/A	N/A	79	5	0	16	93.75	6.25	0
15.4 Self-talk	Motivation	2	0.1	79	16	0	5	87.5	12.5	0
1.3 Goal setting (outcome)	Motivation	3	0.09	76	19	0	5	87.5	12.5	0
4.2 Information about antecedents	Behavioral regulation	2	0.22	76	10	0	14	81.25	18.75	0
9.2 Pros and cons	General attitudes and beliefs	0	1	76	24	0	0	87.5	12.5	0
11.3 Conserving mental resources	Behavioral regulation	1	0.15	76	14	0	10	81.25	18.75	0
1.4 Action planning	Behavioral cueing	4	0.09	74	16	0	10	81.25	18.75	0
5.2 Salience of consequences	Perceived susceptibility/vulnerability	1	0.16	70	25	0	5	81.25	18.75	0
2.2 Feedback on behavior	Motivation	8	0.09	68	32	0	0	81.25	18.75	0
5.5 Anticipated regret	Beliefs about consequences	2	0.06	N/A	N/A	N/A	N/A	87.5	12.5	0

Cells display N/A for the literature synthesis where the BCT was not coded in any of the 277 interventions. Cells display N/A for the expert consensus where the BCT was not considered by experts.

Thus, across all stages (concordance and reconciliation), 92 BCT–MoA links were identified, covering 51 of 61 BCTs and 22 of 26 MoAs. The total of 92 links identified resulted from 37 links identified through concordance and 55 links identified through the reconciliation study. In the reconciliation study, 14 of the 60 resolved links confirmed the literature synthesis finding, 42 confirmed the previous expert consensus finding, and 4 links were based on the marginal evidence provided by both studies (i.e., where .05 ≤ *p*+ ≤.1 in literature synthesis and 70%–79% of experts answered “definitely yes”). Seven of the 60 identified links were for BCTs that had not been identified in the literature study and three links were for BCTs found in the literature synthesis but not in the expert consensus study. In sum, the results provide evidence for 92 hypothesized links, 465 nonlinks, and more evidence needed for the remaining 899 links investigated.

## DEVELOPMENT OF ONLINE, INTERACTIVE TATT

The data from all studies were made available for each of the 1,456 BCT–MoA links in an interactive format. Each link was color coded as a “link,” “nonlink,” “inconclusive,” or “no evidence.” Clicking on any link revealed the evidence from the studies.

**Fig 4:**
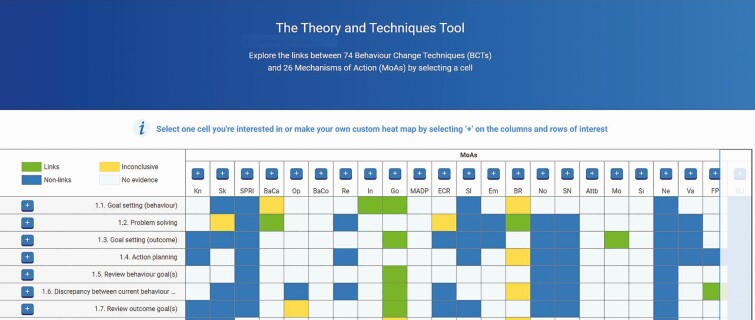
**Screen shot of the home page of the Theory and Technique Tool.** Behavior change techniques are listed on the left column; mechanisms of action are listed on the top row and hovering on the abbreviated title gives the full title and definition. Cells are color coded: green indicates a link, blue a nonlink, yellow inconclusive, and white lack of evidence. Clicking on any cell gives full information of the results of each study for that cell. Supplementary File 3 illustrates the cell data for a link and nonlink. It also illustrates the portal for finding resources or engaging in collaboration related to a given cell.

The online tool can be accessed at (*https://theoryandtechniquetool.humanbehaviourchange.org/*). A screen shot of the home page is shown in Fig. 4, illustrating the matrix of BCTs × MoAs, and the classification of each link is color coded. Supplementary File 3 gives illustrations of the information revealed by clicking on a single BCT–MoA and shows the screens giving access to online resources and the portal for collaboration related to any cell in the matrix.

## DISCUSSION

The purpose of these studies was to examine evidence regarding hypothesized links between BCTs and MoAs and to present the information in an online interactive tool that might be helpful in intervention design, evaluation, and evidence synthesis, identifying priorities for future research, and advancing research and collaborations. Triangulation of the results of the previous literature and consensus studies showed that, overall, the results produced by these two methods of investigating BCT–MoA links converged. This was true both for positive links (“YES” responses) and for nonlinks (“NO” responses) and for each MoA (except “norms,” which was not found in the literature study). Examination of each possible BCT–MoA link revealed concordance between the literature study (277 interventions) and expert consensus (*n* = 100) on 37 links; this was extended to 92 based on the reconciliation study involving a further 16 experts. Hence, this triangulation study provides the first systematic evidence of (a) 92 hypothesized BCT–MoA links that could be targeted or evaluated in interventions, (b) 465 links that this evidence suggests do not exist, and (c) more research being needed to resolve the status of the remaining links. Due to an error, data are lacking for the 11 omitted links and data from the 6 incorrectly included were removed from further analyses. Since data are provided separately for each potential link, these errors have no additional implications for other links.

The reconciliation study was successful in resolving over a third (60/179) of the inconclusive results of the previous two studies. The reconciliation experts were more likely to agree with the previous expert consensus exercise than with links identified in the literature, perhaps, in part due to the similarity of the methods. However, the reconciliation experts had much more information available than the experts in the consensus study, and comments made by experts during the discussion round made it clear that the experts were indeed using the evidence from both previous studies in addition to their own judgment and the judgments of other reconciliation experts. Where there were differences between the literature synthesis and expert consensus, the reconciliation experts discussed possible explanations, including poor methodology in some and the recent specification of BCTs and MoAs, which would not have been available to earlier literature. Nevertheless, the judgments of the reconciliation experts are more likely to be in accord with the previous judges for several reasons. First, the literature synthesis study is restricted to what has actually been done, whereas the judgments, of both groups of experts, address what is theoretically possible. Second, the expert opinion in both the original consensus study and the current reconciliation study reflects current evidence, whereas published studies synthesized in the literature synthesis study were based on the history of evidence available to authors at the time of planning and development of the interventions. Supplementary File 1 provides specific information for each MoA indicating the frequency of observed relationships of each BCT with each MoA in the two studies. No data are provided for the MoA “norms” as this was not found in the literature study. This may simply be a limitation of the studies included in the literature study or it may be that interventions tend to address *subjective* norms as an MoA while norms are seen as moderating variables.

The results have been integrated into an online TATT that allows the user to explore evidence for each possible link, presenting data from the literature, consensus, and reconciliation studies. The online tool (*https://theoryandtechniquetool.humanbehaviourchange.org/*) allows the user to examine results for each of the three studies for each possible BCT–MoA link. Users can also post comments, upload information about links (e.g., new evidence or information about ongoing or planned research), and suggest the possibility of collaborative research for underinvestigated links.

Finding that the two distinct methods, literature synthesis and expert consensus, produce comparable results gives stronger evidence of the replicability of the findings than would be achieved by two studies repeating the same methods. This provides a more secure basis for designing theory-based interventions and for interpreting the theoretical basis of reported interventions than has hitherto been possible. For example, in designing an intervention intended to work by enhancing reinforcement mechanisms, the results suggest that it would be advisable to include nonspecific and social rewards but that instruction on how to perform the behavior would be ineffective (although it might be effective via some other mechanism of action). While this example is immediately obvious, the detailed evidence for each MoA available via the online tool enables the intervention designer to move beyond the specific example illustrated in Fig. 3 and to access the information they need rather than relying on memory, common sense, or further interpretation of the theory underlying the MoA.

The substantial number of links shared by the two studies provides a resource reflecting both current expertise and practice in published interventions for selecting BCTs that might be used to change theoretical constructs that are hypothesized to mediate effects on behavior. BCTs have been identified for each MoA and, since the MoAs investigated address the main theoretical domains and commonly theorized constructs, the links identified offer a range of potential BCTs that might be applicable within a wide range of theoretical frameworks. These links may also prove helpful in suggesting alternative theoretical explanations of the effects of BCTs on behavior in intervention studies, especially those conducted without an explicit theoretical base or where the proposed theoretical basis cannot account for the findings. Further, the links may also assist the synthesis and interpretation of evidence across intervention studies, which had varying theoretical bases as illustrated by Gardner et al.’s examination of theoretically diverse interventions using audit and feedback to change behavior. In their method, “…behavior change intervention is deconstructed into component techniques, which are then mapped onto the most relevant behavior change theory or theories…” ([19], p. 1619). They investigated BCTs, including self-monitoring and action plans, by mapping them on to core MoAs of Control Theory. The findings indicated that audit and feedback could be described in terms of component BCTs and an analysis of an updated review with more data found that the intervention was more effective when a theoretically coherent combination of BCTs targeting key MoAs was included [20]. Thus, the link between BCTs and MoAs within a theory may prove useful in clarifying what would otherwise be heterogeneous interventions and results.

The findings also indicate agreement about BCTs that are *unlikely* to influence MoAs providing a further resource that might prove helpful, especially to intervention designers (i.e., what to avoid). Nevertheless, there continues to be a large number of links where our results are inconclusive. To some extent, this reflects the recency of this field of research but also the lack of an integrating framework that enables areas requiring more evidence to be identified. The online TATT may assist in integrating evidence. However, in some cases, inconclusive results can occur when a BCT may act through several MoAs and so the links found in the literature study for any particular MoA might fail to meet our preset criterion. If so, then these BCTs would be particularly useful as they might activate several MoAs simultaneously. They can be identified in the online tool as being near criterion level for the literature study on several MoAs or linked by experts to several MoAs. For example, BCT 1.1 “goal setting (behaviour)” is strongly linked by experts to MoAs “Intentions,” “Goals,” and “Motivation.” There may also be instances where BCTs are most effective in combination with others. BCTs working via many MoAs may be particularly valuable as they may be tapping more general underlying mechanisms. As a result, these BCTs might be particularly useful in practice.

The majority of experts recruited to the reconciliation study were from the UK, reflecting the origin and continuing interest in research on BCTs in that country. It is possible that different results might have been obtained with experts from other countries, although there are no a priori reasons to expect their responses to differ. A further limitation is that this study could only investigate links with the 56 BCTs shared by the two previous studies.

It is important to keep in mind that these are *hypothesized* BCT–MoA links and it has not been empirically demonstrated that the BCT works via the MoA; further work is needed to ascertain whether the specified technique is able to activate the hypothesized mechanism and, then, in turn, elicit change in behavior. Moreover, although there is concordance between the findings obtained by the two different approaches, they are not completely independent. On the one hand, the experts in the consensus study are likely to have influenced the content of behavior change interventions by having published in the field and, on the other hand, their judgments about links likely to be effective may have derived in part from their knowledge of the literature. The finding of concordance between the results of the two methods provides confirmation of the knowledge base in this field as represented both by a systematic search of relevant literature and by the informed opinions of those working in the field and, therefore, goes beyond the value of either method alone. In addition, by identifying and reconciling discrepant findings, the results go beyond simple confirmation of the quantitative findings [11].

Links may also have been shared by the two methods as both are informed by dominant behavior change theories [21]. For example, it is relatively easy to see how BCTs might be linked to MoA “intentions” if the intervention designers in the literature synthesis studies and the experts in the consensus studies were influenced by the Theory of Planned Behavior [22] or other theories proposing a critical decision point in rational, reflective behavior change processes as in theories involving stages of change [23]. It is also likely that, if interventions have been dominated by such theories, there will be less evidence for BCT–MoA links where the MoA is an automatic, associative, and impulsive process as proposed in dual processing theories [24]. This may change as greater specification of nonreflective processes is achieved, for example, in identifying attributes of the proximal physical environment that prompt behavior [25].

The concordant links may prove particularly useful in developing theory-based interventions. Intervention developers have been criticized for the limited connections between the proposed theoretical basis for their intervention and its actual implementation [26,27], and the current findings may make this easier and, therefore, more likely to fulfill the aim of having interventions that are truly based on theory [28–30]. In a similar manner, Intervention Mapping proposes linkage between behavior change methods and determinants of behavior [31] based on theory and informal input from experts rather than formal triangulation of described methods. The current results reflect hypotheses about the links between BCTs and MoAs in general; thus, it is possible that there are contexts and behavioral domains where an observed link (or nonlink) is more or less likely to emerge. It will be important to investigate how populations and settings moderate the relationship between intervention content (i.e., BCTs) and MoA as proposed to be investigated in the ontology of behavior change interventions [32].

Theoretical constructs are hypothesized to influence behavior in tandem with other theoretical constructs within each theory, a process included within Intervention Mapping as the “parameters” within which one might expect a behavior change method to have an effect [31]. In the literature study, a single MoA was frequently targeted by more than one BCT in a given study and one BCT was targeting more than one MoA. The co-occurrence of BCTs over several studies might hint at a shared underlying theory and this is currently under investigation as part of the current programme of research [33].

## CONCLUSION

The triangulation of the literature synthesis and expert consensus studies has resulted in a replicable set of hypothesized links between BCTs and MoAs, as well as a set of hypothesized nonlinks of BCTs that are unlikely to influence a particular MoA. This has resulted in an online resource (see the interactive online TATT), which can be used to guide intervention development and the theoretical interpretation of results of behavior change interventions. Additionally, this study revealed key areas where experts disagree on potential BCT–MoA links, offering ample opportunity for further research to strengthen the TATT. Hence, we hope this work will provide the basis for more systematic, coordinated research studies to examine and strengthen the evidence base underlying behavior change interventions.

## Supplementary Material

ibaa050_suppl_Supplementary-File-1

ibaa050_suppl_Supplementary-File-2

ibaa050_suppl_Supplementary-File-3

ibaa050_suppl_Supplementary-File-4
