# Supplementary material for: Development of an online tool for linking behavior change techniques and mechanisms of action based on triangulation of findings from literature synthesis and expert consensus
Source: Transl Behav Med. 2020 Aug 4;11(5):1049–65. doi: 10.1093/tbm/ibaa050 (PMC8158171; doi:10.1093/tbm/ibaa050)
Supplement: ibaa050_suppl_Supplementary-File-1 [file ibaa050_suppl_supplementary-file-1.docx]

**Supplement 1: tables S1-S8**

**Tables**

*S1:* Evidence of a link in literature synthesis study (Carey et al., in press), evidence of ‘definitely no’ link in the expert consensus study (Connell et al., in press)

*S2:* Evidence of a link in the literature synthesis study (Carey et al., in press), and no link or absent in the expert consensus study (Connell et al., in press)

*S3:* No evidence of a link in the literature synthesis study (Carey et al., in press), evidence indicates there was ‘definitely’ a link in the expert consensus study (Connell et al., in press)

*S4:* Marginal evidence in either the literature synthesis study (Carey et al., in press) (.05<= p<=.1), and/or the expert consensus study (Connell et al., in press) (70-79% of experts answered ‘definitely yes’ or “definitely no”)

*S5:* Links agreed to be absent in comparison of matrices from the two studies [i.e. links for which there was no evidence in literature synthesis study, (Carey et al., in press) evidence of no link in the expert consensus study (Connell et al., in press)]

*S6:* Links agreed to be absent in reconciliation study (i.e. 80% or more of experts in the current study agreed there is no link)

*S7*: Predicting expert consensus from literature study for each MoA for ‘YES’ and ‘NO’ responses (Multilevel modelling: intercept (Int) and slope (Slp); with and without p=1 BCTs)

*S8:* Multilevel model predicting judgements of expert consensus from the literature synthesis with and without p=1: estimated beta weights (standard error) for fixed effects and variances (standard error) for random effects.

*Table S1:* Evidence of a link in literature synthesis study (Carey et al., in press), evidence of ‘definitely no’ link in the expert consensus study (Connell et al., in press)

|  |  | **Literature Synthesis** | | **Expert Consensus** | | | |
| --- | --- | --- | --- | --- | --- | --- | --- |
| **Behaviour Change Technique** | **Mechanism of Action** | Frequency (# papers) | P value | % Experts (Def Yes) | % Experts (Possibly) | % Experts (Don’t Know) | % Experts (Def No) |
| 2.1 Monitoring of Behaviour by Others without Feedback | Needs | 1 | .019 | 0 | 11 | 0 | 89 |
| 2.7 Feedback on Outcomes of Behaviour | Subjective Norms | 5 | .02 | 0 | 10 | 10 | 80 |
| 12.3 Avoidance / Reducing Exposure to Cues for the Behaviour | Needs | 1 | .027 | 5 | 5 | 0 | 90 |

Notes: The *p* value, in this context, represent an index of likelihood of relationship, whereby low values denote higher likelihood; Experts n = 21 unless specified otherwise.

*Table S2:* Evidence of a link in the literature synthesis study (Carey et al., in press),, and no link or absent in the expert consensus study (Connell et al., in press)

|  | | **Literature Synthesis** | | **Expert Consensus** | | | |
| --- | --- | --- | --- | --- | --- | --- | --- |
| **Behaviour Change Technique** | **Mechanism of Action** | Frequency (# papers) | *p* value | % Experts  (Yes) | % Experts (Possibly) | % Experts (Don’t Know) | % Experts (No) |
| 13.3 Incompatible Beliefs | Attitude towards the Behaviour | 1 | .048 | N/A | N/A | N/A | N/A |
| 13.2 Framing / Reframing | Self-Image | 2 | 0.049 | 5 | 57 | 5 | 33 |
| 15.2 Mental Rehearsal of Successful Performance | Motivation | 2 | 0.008 | N/A | N/A | N/A | N/A |
| 1.2 Problem Solving | Skills | 18 | 0.04 | 43 | 24 | 5 | 28 |
| 1.2 Problem Solving | Environment Context and Resources | 9 | 0.03 | 43 | 24 | 0 | 33 |
| 8.2 Behaviour Substitution | Behavioural Regulation | 5 | 0.016 | 79 | 5 | 0 | 16 |
| 5.3 Information about Social and Environmental Consequences | Attitude towards the Behaviour | 16 | <0.001 | 76 | 5 | 9.5 | 9.5 |
| 7.1 Prompts/Cues | Memory, Attention, and Decision Processes | 8 | <0.001 | 76 | 24 | 0 | 0 |
| 4.1 Instruction on how to Perform the Behaviour | Knowledge | 17 | 0.013 | 74 | 18 | 1 | 7 |
| 8.4 Habit Reversal | Behavioural Regulation | 4 | 0.006 | 74 | 21 | 0 | 5 |
| 8.4 Habit Reversal | Behavioural Cueing | 2 | 0.023 | 74 | 26 | 0 | 0 |
| 8.6 Generalisation of the Target Behaviour | Skills | 2 | 0.047 | 74 | 10 | 0 | 16 |
| 2.4 Self-Monitoring of Outcomes of Behaviour | Behavioural Regulation | 5 | 0.024 | 71 | 24 | 0 | 5 |
| 1.8 Behavioural Contract | Goals | 4 | 0.002 | 70 | 0 | 20 | 10 |
| 2.3 Self-Monitoring of Behaviour | Behavioural Regulation | 18 | <0.001 | 70 | 15 | 0 | 15 |
| 5.6 Information about Emotional Consequences | Emotion | 2 | 0.02 | 70 | 10 | 5 | 15 |
| 5.1 Information about Health Consequences | Attitude towards the Behaviour | 19 | <0.001 | 68 | 32 | 5 | 11 |
| 9.1 Credible Source | General Attitudes / Beliefs | 2 | 0.007 | 68 | 26 | 0 | 6 |
| 5.3 Information about Social and Environmental Consequences | Knowledge | 13 | 0.002 | 67 | 24 | 0 | 9 |
| 12.2 Restructuring the Social Environment | Social Influences | 6 | <0.001 | 65 | 5 | 5 | 25 |
| 5.1 Information about Health Consequences | Intention | 28 | 0.004 | 63 | 26 | 0 | 11 |
| 11.2 Reduce Negative Emotions | Beliefs about Capabilities | 12 | 0.039 | 62 | 14 | 5 | 19 |
| 5.6 Information about Emotional Consequences | Attitude towards the Behaviour | 5 | 0.006 | 60 | 35 | 0 | 5 |
| 1.4 Action Planning | Behavioural Regulation | 14 | 0.001 | 58 | 16 | 0 | 26 |
| 2.1 Monitoring of Behaviour by Others without Feedback | Social Influences | 2 | 0.036 | 58 | 37 | 0 | 5 |
| 6.1 Demonstration of the Behaviour | Beliefs about Capabilities | 60 | 0.003 | 58 | 37 | 5 | 0 |
| 8.4 Habit Reversal | Memory, Attention, and Decision Processes | 2 | 0.036 | 58 | 16 | 5 | 21 |
| 10.1 Material Incentive (behaviour) | Attitude towards the behaviour | 1 | 0.048 | 58 | 21 | 0 | 21 |
| 5.2 Salience of Consequences | Attitude towards the Behaviour | 4 | 0.025 | 55 | 30 | 0 | 15 |
| 6.3 Information about Others’ Approval | Intention | 12 | 0.043 | 53 | 32 | 5 | 10 |
| 8.3 Habit Formation | Behavioural Regulation | 3 | 0.024 | 45 | 20 | 0 | 35 |
| 2.2 Feedback on Behaviour | Knowledge | 13 | 0.013 | 42 | 32 | 0 | 26 |
| 1.6 Discrepancy between current behaviour and goal | Behavioural Regulation | 3 | 0.019 | 33 | 24 | 0 | 43 |
| 1.1 Goal Setting (behaviour) | Behavioural Regulation | 15 | 0.003 | 35 | 35 | 0 | 30 |
| 6.1 Demonstration of the Behaviour | Skills | 17 | 0.020 | 26 | 37 | 5 | 32 |
| 1.9 Commitment | Values | 1 | 0.039 | 21 | 42 | 5 | 32 |
| 9.1 Credible Source | Social / Professional Role and Identity | 4 | 0.023 | 11 | 58 | 0 | 31 |
| 3.1 Social Support (Unspecified) | Social / Professional Role and Identity | 5 | 0.037 | 10 | 28 | 4 | 58 |
| 2.2 Feedback on Behaviour | Subjective Norms | 19 | <0.001 | 5 | 16 | 11 | 68 |
| 9.2 Pros and Cons | Feedback Processes | 3 | 0.005 | 0 | 29 | 0 | 71 |
| 7.8 Associative Learning | Reinforcement | 1 | 0.038 | N/A | N/A | N/A | N/A |
| 10.7 Self-Incentive | Motivation | 1 | 0.036 | N/A | N/A | N/A | N/A |
| 13.5 Identity Associated with Changed Behaviour | Values | 1 | 0.016 | N/A | N/A | N/A | N/A |
| 15.2 Mental Rehearsal of Successful Performance | Values | 1 | 0.026 | N/A | N/A | N/A | N/A |
| 15.3 Focus on Past Success | Beliefs about Capabilities | 23 | <0.001 | N/A | N/A | N/A | N/A |

Notes: The *p* value, in this context, represent an index of likelihood of relationship, whereby low values denote higher likelihood; Experts n = 21 unless specified otherwise; N/A where BCT was not considered by experts.

*Table S3:* No evidence of a link in the literature synthesis study (Carey et al., in press), evidence indicates there was ‘definitely’ a link in the expert consensus study (Connell et al., in press)

|  | | **Literature Synthesis Study** | | **Expert Consensus Study** | | | |
| --- | --- | --- | --- | --- | --- | --- | --- |
| **Behaviour Change Technique** | **Mechanism of Action** | Frequency (# papers) | *p* value | % Experts  (Yes) | % Experts (Possibly) | % Experts (Don’t Know) | % Experts (No) |
| 1.2 Problem Solving | Behavioural Regulation | 13 | .16 | 100 | 0 | 0 | 0 |
| 1.6 Discrepancy between Current Behaviour and Goal | Feedback Processes | 1 | .07 | 100 | 0 | 0 | 0 |
| 11.3 Conserving Mental Resources | Memory, Attention, Decision Processes | 1 | .06 | 100 | 0 | 0 | 0 |
| 10.8 Incentive (Outcome) | Motivation | N/A | N/A | 100 | 0 | 0 | 0 |
| 1.1 Goal Setting (behaviour) | Intention | 17 | .34 | 95 | 5 | 0 | 0 |
| 1.1 Goal Setting (behaviour) | Goals | 4 | .23 | 95 | 0 | 5 | 0 |
| 1.5 Review Behaviour Goals | Feedback Processes | 0 | 1 | 95 | 5 | 0 | 0 |
| 2.2 Feedback on Behaviour | Feedback Processes | 3 | .07 | 95 | 5 | 0 | 0 |
| 10.1 Material Incentive (Behaviour) | Reinforcement | 0 | 1 | 95 | 5 | 0 | 0 |
| 10.4 Social Reward | Social Influences | 2 | .55 | 95 | 5 | 0 | 0 |
| 11.2 Reduce Negative Emotions | Emotion | 1 | .21 | 95 | 0 | 5 | 0 |
| 12.3 Avoidance/Reducing Exposure to Cues | Environmental Context & Resources | 1 | .24 | 95 | 5 | 0 | 0 |
| 12.3 Avoidance/Reducing Exposure to Cues | Behavioural Cueing | 0 | 1 | 95 | 5 | 0 | 0 |
| 12.5 Adding Objects to the Environment | Behavioural Cueing | 2 | .11 | 95 | 5 | 0 | 0 |
| 14.10 Remove Punishment | Reinforcement | 0 | 1 | 95 | 5 | 0 | 0 |
| 10.8 Incentive (Outcome) | Intention | N/A | N/A | 95 | 0 | 0 | 5 |
| 1.5 Review Behaviour Goals | Goals | 2 | .07 | 90 | 0 | 0 | 10 |
| 6.2 Social Comparison | Norms | 0 | 1 | 90 | 5 | 0 | 5 |
| 6.2 Social Comparison | Feedback Processes | 2 | .20 | 90 | 5 | 0 | 5 |
| 11.2 Reduce Negative Emotions | Behavioural Regulation | 0 | 1 | 90 | 5 | 5 | 0 |
| 7.5 Remove Aversive Stimulus | Environmental Context & Resources | N/A | N/A | 90 | 5 | 5 | 0 |
| 10.8 Incentive (Outcome) | Beliefs about Consequences | N/A | N/A | 90 | 5 | 0 | 5 |
| 10.8 Incentive (Outcome) | Reinforcement | N/A | N/A | 90 | 0 | 5 | 5 |
| 10.10 Reward (Outcome) | Reinforcement | N/A | N/A | 90 | 5 | 5 | 0 |
| 10.10 Reward (Outcome) | Motivation | N/A | N/A | 90 | 10 | 0 | 0 |
| 6.3 Information about Other’s Approval | Norms | 0 | 1 | 90 | 5 | 5 | 0 |
| 10.2 Material Reward (Behaviour) | Reinforcement | 0 | 1 | 90 | 0 | 5 | 5 |
| 2.6 Biofeedback | Feedback Processes | 0 | 1 | 89 | 11 | 0 | 0 |
| 1.3 Goal Setting (Outcome) | Intention | 5 | .27 | 86 | 9 | 0 | 5 |
| 4.2 Information about Antecedents | Knowledge | 3 | .051 | 86 | 14 | 0 | 0 |
| 5.3 Information about Social and Environmental Consequences | Motivation | 5 | .24 | 86 | 5 | 0 | 9 |
| 9.3 Comparative Imagining of Future Outcomes | Motivation | 1 | .26 | 86 | 9 | 0 | 5 |
| 10.10 Reward (Outcome) | Beliefs about Consequences | N/A | N/A | 85 | 5 | 0 | 10 |
| 5.2 Salience of Consequences | Beliefs about Consequences | 2 | .46 | 85 | 10 | 0 | 5 |
| 16.3 Vicarious Consequences | Social Learning/Imitation | 1 | .10 | 85 | 5 | 5 | 5 |
| 1.9 Commitment | Intention | 5 | .13 | 84 | 16 | 0 | 0 |
| 1.9 Commitment | Motivation | 1 | .53 | 84 | 16 | 0 | 0 |
| 3.3 Social Support (Emotional) | Emotion | 1 | .16 | 84 | 16 | 0 | 0 |
| 6.3 Information about Others’ Approval | Social Influences | 4 | .19 | 84 | 16 | 0 | 0 |
| 8.4 Habit Reversal | Skills | 1 | .58 | 84 | 5 | 0 | 11 |
| 10.1 Material Incentive (Behaviour) | Motivation | 0 | 1 | 84 | 16 | 0 | 0 |
| 10.3 Non-specific Reward | Motivation | 1 | .29 | 84 | 11 | 0 | 5 |
| 4.2 Information about Antecedents | Behavioural Cueing | 0 | 1 | 81 | 5 | 0 | 14 |
| 8.7 Graded Tasks | Skills | 4 | .42 | 81 | 14 | 0 | 5 |
| 9.3 Comparative Imagining of Future Outcomes | Intention | 1 | .66 | 81 | 10 | 0 | 9 |
| 2.4 Self-Monitoring of Outcomes of Behaviour | Feedback Processes | 1 | .20 | 81 | 9 | 5 | 5 |
| 10.4 Social Reward | Motivation | 3 | .20 | 81 | 14 | 0 | 5 |
| 1.1 Goal Setting (behaviour) | Motivation | 3 | .80 | 80 | 20 | 0 | 0 |
| 1.8 Behavioural Contract | Intention | 4 | .44 | 80 | 5 | 5 | 10 |
| 2.3 Self-Monitoring of Behaviour | Feedback Processes | 2 | .16 | 80 | 15 | 0 | 5 |
| 5.4 Monitoring of Emotional Consequences | Emotion | 0 | 1 | 80 | 15 | 0 | 5 |
| 12.2 Restructuring the Social Environment | Behavioural Cueing | 0 | 1 | 80 | 10 | 0 | 10 |
| 16.3 Vicarious Consequences | Norms | 0 | 1 | 80 | 5 | 10 | 5 |

*Note:* Cells display N/A for the literature synthesis where the BCT was not coded in any of the 277 interventions. Cells display N/A for the expert consensus where the BCT was not considered by experts.

*Table S4:* Marginal evidence in either the literature synthesis study (Carey et al., in press) (.05<= p<=.1), and/or the expert consensus study (Connell et al., in press) (70-79% of experts answered ‘definitely yes’ or “definitely no”)

|  | | **Literature Synthesis Study** | | **Expert Consensus Study** | | | |
| --- | --- | --- | --- | --- | --- | --- | --- |
| **Behaviour Change Technique** | **Mechanism of Action** | Frequency (# papers) | *p* value | % Experts (Yes) | % Experts (Possibly) | % Experts (Don’t Know) | % Experts (No) |
| 1.7 Review Outcome Goals | Intention | 1 | 0.63 | 79 | 16 | 0 | 5 |
| 1.7 Review Outcome Goals | Feedback Processes | 0 | 1 | 79 | 21 | 0 | 0 |
| 1.9 Commitment | Goals | 1 | 0.29 | 79 | 21 | 0 | 0 |
| 2.6 Biofeedback | Knowledge | 0 | 1 | 79 | 16 | 0 | 5 |
| 3.3 Social Support (emotional) | Social Influences | 2 | 0.15 | 79 | 21 | 0 | 0 |
| 4.1 Instruction on how to Perform the Behaviour | Beliefs about Capabilities | 62 | 0.08 | 79 | 17 | 0 | 4 |
| 8.4 Habit Reversal | Intention | 1 | 0.80 | 79 | 16 | 0 | 5 |
| 8.2 Behaviour Substitution | Behavioural Cueing | 2 | 0.08 | 79 | 21 | 0 | 0 |
| 9.1 Credible Source | Attitude towards the Behaviour | 7 | 0.09 | 79 | 21 | 0 | 0 |
| 10.1 Material Incentive (behaviour) | Beliefs about Consequences | 0 | 1 | 79 | 11 | 0 | 11 |
| 10.6 Non-specific Incentive | Reinforcement | N/A | N/A | 79 | 5 | 0 | 16 |
| 14.10 Remove Punishment | Beliefs about Consequences | 0 | 1 | 79 | 10.5 | 0 | 10.5 |
| 15.4 Self-talk | Motivation | 2 | 0.1 | 79 | 16 | 0 | 5 |
| 1.3 Goal Setting (outcome) | Motivation | 3 | 0.09 | 76 | 19 | 0 | 5 |
| 2.4 Self-Monitoring of Outcomes of Behaviour | Beliefs about Capabilities | 7 | 0.73 | 76 | 24 | 0 | 0 |
| 2.4 Self-Monitoring of Outcomes of Behaviour | Motivation | 0 | 1 | 76 | 5 | 9.5 | 9.5 |
| 3.2 Social Support (practical) | Behavioural Cueing | 0 | 1 | 76 | 19 | 0 | 5 |
| 4.2 Information about Antecedents | Behavioural Regulation | 2 | 0.22 | 76 | 10 | 0 | 14 |
| 8.7 Graded Tasks | Motivation | 1 | 0.84 | 76 | 19 | 0 | 5 |
| 9.2 Pros and Cons | General Attitudes & Beliefs | 0 | 1 | 76 | 24 | 0 | 0 |
| 11.3 Conserving Mental Resources | Behavioural Regulation | 1 | 0.15 | 76 | 14 | 0 | 10 |
| 15.1 Verbal Persuasion about Capability | Social Influences | 1 | 0.80 | 76 | 10 | 0 | 14 |
| 15.1 Verbal Persuasion about Capability | Motivation | 1 | 0.76 | 76 | 14 | 0 | 10 |
| 16.3 Vicarious Consequences | Subjective Norms | 1 | 0.56 | 75 | 20 | 0 | 5 |
| 1.4 Action Planning | Goals | 3 | 0.31 | 74 | 16 | 0 | 10 |
| 1.4 Action Planning | Behavioural Cueing | 4 | 0.09 | 74 | 16 | 0 | 10 |
| 1.7 Review Outcome Goals | Motivation | 0 | 1 | 74 | 16 | 0 | 10 |
| 2.1 Monitoring of Behaviour by Others without Feedback | Behavioural Cueing | 0 | 1 | 74 | 26 | 0 | 0 |
| 2.6 Biofeedback | Reinforcement | 0 | 1 | 74 | 21 | 0 | 5 |
| 2.6 Biofeedback | Motivation | 1 | 0.26 | 74 | 21 | 0 | 5 |
| 8.6 Generalisation of the Target Behaviour | Beliefs about Capabilities | 0 | 1 | 74 | 21 | 0 | 5 |
| 9.1 Credible Source | Social Influences | 5 | 0.23 | 74 | 26 | 0 | 0 |
| 10.1 Material Incentive (behaviour) | Intention | 0 | 1 | 74 | 26 | 0 | 0 |
| 10.6 Non-specific Incentive | Intention | N/A | N/A | 74 | 16 | 0 | 10 |
| 10.6 Non-specific Incentive | Motivation | N/A | N/A | 74 | 16 | 0 | 10 |
| 14.10 Remove Punishment | Motivation | 0 | 1 | 74 | 21 | 0 | 5 |
| 6.2 Social Comparison | Values | 0 | 1 | 71 | 19 | 0 | 10 |
| 9.2 Pros and Cons | Memory, Attention, & Decision Processes | 0 | 1 | 71 | 14 | 5 | 10 |
| 9.3 Comparative Imagining of Future Outcomes | Attitude towards the Behaviour | 0 | 1 | 71 | 14 | 5 | 10 |
| 10.8 Incentive (outcome) | Goals | N/A | N/A | 71 | 14.5 | 0 | 14.5 |
| 12.6 Body Changes | Self-Image | 0 | 1 | 71 | 19 | 0 | 10 |
| 13.2 Framing / Reframing | Beliefs about Consequences | 5 | 0.25 | 71 | 24 | 5 | 0 |
| 13.2 Framing / Reframing | Motivation | 3 | 0.12 | 71 | 29 | 0 | 0 |
| 15.4 Self-talk | Beliefs about Capabilities | 8 | 0.054 | 42 | 47 | 0 | 11 |
| 15.1 Verbal Persuasion about Capability | Self-Image | 0 | 1 | 71 | 14.5 | 0 | 14.5 |
| 1.5 Review Behaviour Goals | Intention | 5 | 0.11 | 70 | 15 | 0 | 15 |
| 1.5 Review Behaviour Goals | Behavioural Regulation | 2 | 0.30 | 70 | 15 | 0 | 15 |
| 2.7 Feedback on Outcomes of Behaviour | Beliefs about Capabilities | 7 | 0.82 | 70 | 20 | 0 | 10 |
| 2.7 Feedback on Outcomes of Behaviour | Reinforcement | 1 | 0.34 | 70 | 15 | 5 | 10 |
| 5.2 Salience of Consequences | Perceived Susceptibility/Vulnerability | 1 | 0.16 | 70 | 25 | 0 | 5 |
| 8.3 Habit Formation | Memory, Attention, & Decision Processes | 1 | 0.22 | 70 | 10 | 5 | 15 |
| 8.3 Habit Formation | Environmental Context & Resources | 0 | 1 | 70 | 10 | 5 | 15 |
| 16.3 Vicarious Consequences | Social Influences | 0 | 1 | 70 | 25 | 0 | 5 |
| 2.2 Feedback on Behaviour | Motivation | 8 | 0.09 | 68 | 32 | 0 | 0 |
| 11.3 Conserving Mental Resources | Environmental Context and Resources | 1 | 0.07 | 67 | 28 | 0 | 5 |
| 9.1 Credible Source | Social Learning/Imitation | 2 | 0.07 | 63 | 21 | 0 | 16 |
| 2.2 Feedback on Behaviour | Reinforcement | 4 | 0.08 | 58 | 26 | 0 | 16 |
| 13.2 Framing / Reframing | General Attitude & Beliefs | 1 | 0.08 | 52 | 38 | 5 | 5 |
| 10.4 Social Reward | Beliefs about Capabilities | 18 | 0.09 | 43 | 29 | 0 | 28 |
| 1.1 Goal Setting (behaviour) | Beliefs about Capabilities | 44 | 0.06 | 35 | 30 | 0 | 35 |
| 8.3 Habit Formation | Motivation | 2 | 0.06 | 35 | 20 | 0 | 45 |
| 1.8 Behavioural Contract | Behavioural Regulation | 3 | 0.06 | 25 | 15 | 5 | 55 |
| 2.1 Monitoring of Behaviour by Others without Feedback | Reinforcement | 1 | 0.09 | 16 | 31.5 | 10.5 | 42 |
| 10.2 Material Reward (behaviour) | Goals | 1 | 0.10 | 16 | 58 | 10 | 16 |
| 12.6 Body Changes | Emotion | 1 | 0.06 | 14 | 29 | 9 | 48 |
| 1.7 Review Outcome Goals | Optimism | 1 | 0.06 | 11 | 53 | 5 | 31 |
| 1.9 Commitment | Memory, Attention, & Decision Processes | 2 | 0.06 | 11 | 26 | 16 | 47 |
| 2.6 Biofeedback | Beliefs about Capabilities | 5 | 0.09 | 11 | 42 | 0 | 47 |
| 8.1 Behavioural Practice / Rehearsal | Behavioural Cueing | 5 | 0.06 | 10 | 33 | 0 | 57 |
| 4.2 Information about Antecedents | Skills | 3 | 0.10 | 9 | 5 | 0 | 86 |
| 10.3 Non-specific Reward | Self-Image | 1 | 0.056 | 5 | 0 | 0 | 95 |
| 5.5 Anticipated Regret | Beliefs about Consequences | 2 | 0.06 | N/A | N/A | N/A | N/A |
| 7.8 Associative Learning | Behavioural Cueing | 1 | 0.051 | N/A | N/A | N/A | N/A |
| 7.8 Associative Learning | Memory, Attention, & Decision Processes | 1 | 0.07 | N/A | N/A | N/A | N/A |
| 13.4 Valued Self-Identity | Intention | 2 | .055 | N/A | N/A | N/A | N/A |
| 13.5 Identity Associated with Changed Behaviour | Social/Professional Role & Identity | 1 | 0.09 | N/A | N/A | N/A | N/A |

*Note:* Cells display N/A for the literature synthesis where the BCT was not coded in any of the 277 interventions. Cells display N/A for the expert consensus where the BCT was not considered by experts.

*Table S5:* Links agreed to be absent in comparison of matrices from the two studies [i.e. links for which there was no evidence in literature synthesis study, (Carey et al., in press) evidence of no link in the expert consensus study (Connell et al., in press)]

|  |  | **Literature Synthesis Study** | | **Expert Consensus Study** |
| --- | --- | --- | --- | --- |
| **Behaviour Change Technique** | **Mechanism of Action** | Frequency (number of papers) | *p*-value | Proportion Experts  (‘Definitely No’) |
| 1.1 Goal Setting (behaviour) | Social / Professional Role & Identity | 2 | 0.49 | 0.90 |
|  | Social Learning / Imitation | 1 | 0.48 | 0.90 |
|  | Perceived Susceptibility/Vulnerability | 0 | 1 | 0.90 |
|  | Skills | 7 | 0.71 | 0.85 |
|  | Social Influences | 2 | 0.96 | 0.80 |
|  | Needs | 1 | 0.28 | 0.80 |
| 1.2 Problem Solving | Norms | 0 | 1 | 0.95 |
|  | Subjective Norms | 0 | 1 | 0.95 |
|  | Social Learning / Imitation | 0 | 1 | 0.95 |
|  | Social / Professional Role & Identity | 2 | 0.66 | 0.90 |
|  | Needs | 0 | 1 | 0.90 |
|  | Values | 0 | 1 | 0.90 |
|  | Reinforcement | 2 | 0.63 | 0.86 |
|  | Social Influences | 3 | 0.97 | 0.81 |
|  | Emotion | 1 | 0.81 | 0.81 |
|  | General Attitudes & Beliefs | 0 | 1 | 0.81 |
| 1.3 Goal Setting (outcome) | Perceived Susceptibility/Vulnerability | 0 | 1 | 1.00 |
|  | Social/ Professional Role & Identity | 0 | 1 | 0.95 |
|  | Norms | 0 | 1 | 0.95 |
|  | Social Learning / Imitation | 1 | 0.15 | 0.95 |
|  | Skills | 4 | 0.1405 | 0.90 |
|  | Social Influences | 1 | 0.71 | 0.90 |
|  | Subjective Norms | 0 | 1 | 0.90 |
|  | Knowledge | 0 | 1 | 0.86 |
|  | Emotion | 0 | 1 | 0.86 |
|  | Values | 0 | 1 | 0.86 |
|  | General Attitudes & Beliefs | 0 | 1 | 0.86 |
|  | Environmental Context & Resources | 1 | 0.518 | 0.81 |
|  | Needs | 0 | 1 | 0.81 |
| 1.4 Action Planning | Social/ Professional Role & Identity | 2 | 0.39179 | 0.95 |
|  | Norms | 0 | 1 | 0.95 |
|  | Subjective Norms | 2 | 0.94 | 0.95 |
|  | Needs | 0 | 1 | 0.95 |
|  | Social Learning / Imitation | 0 | 1 | 0.95 |
|  | Social Influences | 0 | 1 | 0.89 |
|  | General Attitudes & Beliefs | 0 | 1 | 0.89 |
|  | Perceived Susceptibility/Vulnerability | 0 | 1 | 0.89 |
|  | Knowledge | 3 | 0.8715 | 0.84 |
|  | Reinforcement | 1 | 0.71 | 0.84 |
|  | Values | 1 | 0.20 | 0.84 |
|  | Feedback Processes | 0 | 1 | 0.84 |
| 1.5 Review Behaviour Goals | Social Learning / Imitation | 0 | 1 | 0.90 |
|  | Norms | 0 | 1 | 0.85 |
|  | Perceived Susceptibility/Vulnerability | 0 | 1 | 0.85 |
|  | Social/ Professional Role & Identity | 0 | 1 | 0.80 |
|  | Subjective Norms | 0 | 1 | 0.80 |
|  | Needs | 0 | 1 | 0.80 |
| 1.6 Discrepancy between current behaviour and goal | Social Learning / Imitation | 0 | 1 | 1.00 |
|  | Social/ Professional Role & Identity | 0 | 1 | 0.90 |
|  | Reinforcement | 0 | 1 | 0.90 |
|  | Norms | 0 | 1 | 0.90 |
|  | Subjective Norms | 0 | 1 | 0.90 |
|  | Environmental Context & Resources | 0 | 1 | 0.86 |
|  | Needs | 0 | 1 | 0.86 |
|  | Perceived Susceptibility/Vulnerability | 0 | 1 | 0.86 |
|  | Skills | 0 | 1 | 0.81 |
|  | Optimism | 0 | 1 | 0.81 |
|  | Behavioural Cueing | 0 | 1 | 0.81 |
|  | General Attitudes & Beliefs | 0 | 1 | 0.81 |
| 1.7 Review Outcome Goals | Social/ Professional Role & Identity | 0 | 1 | 0.95 |
|  | Social Influences | 0 | 1 | 0.95 |
|  | Norms | 0 | 1 | 0.95 |
|  | Subjective Norms | 0 | 1 | 0.95 |
|  | Social Learning / Imitation | 0 | 1 | 0.95 |
|  | Environmental Context & Resources | 0 | 1 | 0.89 |
|  | Perceived Susceptibility/Vulnerability | 0 | 1 | 0.89 |
|  | Knowledge | 0 | 1 | 0.84 |
|  | Skills | 1 | 0.422 | 0.84 |
|  | Needs | 0 | 1 | 0.84 |
|  | Behavioural Cueing | 0 | 1 | 0.84 |
| 1.8 Behavioural Contract | Social Learning / Imitation | 0 | 1 | 0.95 |
|  | Needs | 0 | 1 | 0.90 |
|  | Perceived Susceptibility/Vulnerability | 0 | 1 | 0.90 |
|  | Skills | 3 | 0.26 | 0.85 |
|  | Knowledge | 0 | 1 | 0.80 |
| 1.9 Commitment | Skills | 0 | 1 | 0.84 |
|  | Reinforcement | 1 | 0.198 | 0.84 |
|  | Social Learning/Imitation | 0 | 1 | 0.84 |
| 2.1 Monitoring of Behaviour by Others without Feedback | Optimism | 0 | 1 | 0.84 |
| 2.2 Feedback on Behaviour | Needs | 0 | 1 | 0.84 |
|  | Social Learning/Imitation | 0 | 1 | 0.84 |
| 2.3 Self-Monitoring of Behaviour | Social Learning/Imitation | 0 | 1 | 0.95 |
|  | Norms | 0 | 1 | 0.90 |
|  | Subjective Norms | 0 | 1 | 0.90 |
|  | Social/ Professional Role & Identity | 1 | 0.72 | 0.85 |
|  | Needs | 1 | 0.23 | 0.85 |
|  | Values | 0 | 1 | 0.85 |
|  | General Attitudes & Beliefs | 0 | 1 | 0.85 |
|  | Perceived Susceptibility/Vulnerability | 0 | 1 | 0.85 |
|  | Social Influences | 1 | 0.98 | 0.80 |
| 2.4 Self-Monitoring of Outcomes of Behaviour | Norms | 0 | 1 | 1 |
|  | Social Influences | 3 | 0.13 | 0.95 |
|  | Subjective Norms | 0 | 1 | 0.95 |
|  | Needs | 0 | 1 | 0.95 |
|  | Social Learning/Imitation | 0 | 1 | 0.95 |
|  | Social/ Professional Role & Identity | 0 | 1 | 0.9 |
|  | Perceived Susceptibility/Vulnerability | 0 | 1 | 0.9 |
|  | Values | 0 | 1 | 0.86 |
|  | General Attitudes & Beliefs | 0 | 1 | 0.81 |
| 2.5 Monitoring of outcomes of behaviour without feedback | Needs | 0 | 1 | 0.95 |
|  | Social Learning/Imitation | 0 | 1 | 0.95 |
|  | Skills | 0 | 1 | 0.86 |
|  | Social/ Professional Role & Identity | 0 | 1 | 0.86 |
|  | Optimism | 0 | 1 | 0.86 |
|  | Beliefs about Capabilities | 2 | 0.11 | 0.81 |
|  | Memory, Attention, & Decision Processes | 0 | 1 | 0.81 |
|  | Environmental Context & Resources | 0 | 1 | 0.81 |
|  | Norms | 0 | 1 | 0.81 |
|  | Values | 0 | 1 | 0.81 |
|  | General Attitudes & Beliefs | 0 | 1 | 0.81 |
| 2.6 Biofeedback | Social/ Professional Role & Identity | 0 | 1 | 0.89 |
|  | Norms | 0 | 1 | 0.89 |
|  | Subjective Norms | 1 | 0.32 | 0.89 |
|  | Social Learning/Imitation | 0 | 1 | 0.89 |
|  | Social Influences | 0 | 1 | 0.84 |
|  | Values | 0 | 1 | 0.84 |
| 2.7 Feedback on Outcomes of Behaviour | Social Learning/Imitation | 0 | 1 | 0.95 |
|  | Environmental Context & Resources | 0 | 1 | 0.9 |
|  | Norms | 0 | 1 | 0.9 |
|  | Perceived Susceptibility/Vulnerability | 1 | 0.22 | 0.85 |
| 3.1 Social Support (Unspecified) | Skills | 6 | 0.88 | 0.87 |
|  | Perceived Susceptibility/Vulnerability | 0 | 1 | 0.82 |
| 3.2 Social Support (practical) | Perceived Susceptibility/Vulnerability | 0 | 1 | 0.95 |
|  | Feedback Processes | 0 | 1 | 0.9 |
|  | Self-image | 0 | 1 | 0.86 |
|  | Needs | 0 | 1 | 0.86 |
|  | Values | 0 | 1 | 0.81 |
| 3.3 Social Support (emotional) | Feedback Processes | 0 | 1 | 0.84 |
| 4.1 Instruction on how to Perform the Behaviour | Needs | 0 | 1 | 0.9 |
|  | Perceived Susceptibility/Vulnerability | 1 | 0.754 | 0.9 |
|  | Values | 0 | 1 | 0.87 |
|  | Emotion | 1 | 0.84 | 0.84 |
|  | Social/Professional Role & Identity | 2 | 0.70 | 0.82 |
|  | Subjective Norms | 4 | 0.97 | 0.82 |
|  | Norms | 0 | 1 | 0.81 |
| 4.2 Information about Antecedents | Needs | 0 | 1 | 0.95 |
|  | Social/Professional Role & Identity | 0 | 1 | 0.9 |
|  | Norms | 0 | 1 | 0.9 |
|  | Subjective Norms | 0 | 1 | 0.9 |
|  | Values | 0 | 1 | 0.9 |
|  | Feedback Processes | 0 | 1 | 0.9 |
|  | General Attitudes & Beliefs | 0 | 1 | 0.9 |
|  | Skills | 3 | 0.098 | 0.86 |
|  | Self-image | 0 | 1 | 0.86 |
|  | Social Learning / Imitation | 0 | 1 | 0.86 |
|  | Optimism | 0 | 1 | 0.81 |
|  | Reinforcement | 0 | 1 | 0.81 |
|  | Social Influences | 0 | 1 | 0.81 |
|  | Attitude towards the Behaviour | 0 | 1 | 0.81 |
| 5.1 Information about Health Consequences | Reinforcement | 0 | 1 | 0.95 |
|  | Social Learning / Imitation | 0 | 1 | 0.95 |
|  | Skills | 2 | 0.99 | 0.89 |
|  | Optimism | 0 | 1 | 0.89 |
|  | Behavioural Regulation | 1 | 0.999 | 0.89 |
|  | Subjective Norms | 3 | 0.95 | 0.89 |
|  | Social/Professional Role & Identity | 0 | 1 | 0.84 |
|  | Environmental Context & Resources | 0 | 1 | 0.84 |
|  | Self-Image | 1 | 0.60 | 0.84 |
|  | Needs | 0 | 1 | 0.84 |
| 5.2 Salience of Consequences | Skills | 0 | 1 | 0.95 |
|  | Social Learning/Imitation | 0 | 1 | 0.9 |
|  | Optimism | 0 | 1 | 0.85 |
|  | Needs | 0 | 1 | 0.85 |
|  | Social/Professional Role & Identity | 0 | 1 | 0.8 |
|  | Beliefs about Capabilities | 3 | 0.95 | 0.8 |
| 5.3 Information about Social and Environmental Consequences | Skills | 1 | 0.99 | 1 |
|  | Social Learning / Imitation | 0 | 1 | 0.95 |
|  | Behavioural Regulation | 0 | 1 | 0.9 |
|  | Feedback Processes | 2 | 0.17 | 0.9 |
|  | Beliefs about Capabilities | 7 | 0.999 | 0.86 |
|  | Optimism | 0 | 1 | 0.86 |
|  | Needs | 0 | 1 | 0.81 |
| 5.4 Monitoring of emotional consequences | Skills | 1 | 0.13 | 0.95 |
|  | Social/Professional Role & Identity | 0 | 1 | 0.9 |
|  | Memory, Attention, & Decision Processes | 0 | 1 | 0.9 |
|  | Norms | 0 | 1 | 0.9 |
|  | Subjective Norms | 0 | 1 | 0.9 |
|  | Social Learning / Imitation | 0 | 1 | 0.9 |
|  | Environmental Context & Resources | 0 | 1 | 0.85 |
|  | Values | 0 | 1 | 0.85 |
|  | Perceived Susceptibility/Vulnerability | 0 | 1 | 0.8 |
| 5.6 Information about Emotional Consequences | Social Learning / Imitation | 0 | 1 | 0.95 |
|  | Behavioural Cueing | 0 | 1 | 0.9 |
|  | Skills | 0 | 1 | 0.85 |
|  | Social/Professional Role & Identity | 0 | 1 | 0.85 |
|  | Memory, Attention, & Decision Processes | 0 | 1 | 0.85 |
|  | Environmental Context & Resources | 0 | 1 | 0.85 |
|  | Beliefs about Capabilities | 3 | 0.96 | 0.8 |
|  | Social Influences | 0 | 1 | 0.8 |
|  | Norms | 0 | 1 | 0.8 |
|  | Subjective Norms | 1 | 0.69 | 0.8 |
| 6.1 Demonstration of the Behaviour | Needs | 0 | 1 | 0.95 |
|  | Perceived Susceptibility/Vulnerability | 1 | 0.67 | 0.89 |
|  | Self-Image | 1 | 0.63 | 0.84 |
| 6.2 Social Comparison | Skills | 2 | 0.99 | 1.00 |
|  | Needs | 0 | 1 | 0.95 |
|  | Behavioural Regulation | 0 | 1 | 0.86 |
|  | Perceived Susceptibility/Vulnerability | 1 | 0.56 | 0.86 |
| 6.3 Information about Other’s Approval | Skills | 1 | 0.98 | 0.95 |
|  | Needs | 0 | 1 | 0.84 |
| 7.1 Prompts/Cues | Needs | 0 | 1 | 1 |
|  | Perceived Susceptibility/Vulnerability | 1 | 0.43 | 1 |
|  | Knowledge | 4 | 0.50 | 0.95 |
|  | Social/Professional Role & Identity | 1 | 0.62 | 0.95 |
|  | Self-Image | 0 | 1 | 0.95 |
|  | Values | 0 | 1 | 0.95 |
|  | Social Learning / Imitation | 0 | 1 | 0.95 |
|  | General Attitudes & Beliefs | 0 | 1 | 0.95 |
|  | Beliefs about Consequences | 3 | 0.85 | 0.9 |
|  | Norms | 0 | 1 | 0.9 |
|  | Subjective Norms | 0 | 1 | 0.9 |
|  | Feedback Processes | 0 | 1 | 0.9 |
|  | Beliefs about Capabilities | 14 | 0.95 | 0.86 |
|  | Optimism | 1 | 0.43 | 0.86 |
|  | Attitude towards the Behaviour | 2 | 0.86 | 0.86 |
|  | Emotion | 1 | 0.516 | 0.81 |
| 7.5 Remove Aversive Stimulus | Knowledge | N/A | N/A | 1 |
|  | Needs | N/A | N/A | 1 |
|  | Skills | N/A | N/A | 0.95 |
|  | Social/Professional Role & Identity | N/A | N/A | 0.95 |
|  | Values | N/A | N/A | 0.9 |
|  | Subjective Norms | N/A | N/A | 0.86 |
|  | Self-Image | N/A | N/A | 0.86 |
|  | Perceived Susceptibility/Vulnerability | N/A | N/A | 0.86 |
|  | Norms | N/A | N/A | 0.81 |
|  | Feedback Processes | N/A | N/A | 0.81 |
|  | Social Learning / Imitation | N/A | N/A | 0.81 |
| 8.1 Behavioural Practice / Rehearsal | Norms | 0 | 1 | 0.95 |
|  | Needs | 0 | 1 | 0.95 |
|  | Values | 0 | 1 | 0.95 |
|  | Social/Professional Role & Identity | 0 | 1 | 0.9 |
|  | Subjective Norms | 1 | 0.996 | 0.9 |
|  | Social Influences | 1 | 0.99 | 0.86 |
|  | Social Learning / Imitation | 2 | 0.13 | 0.86 |
|  | Perceived Susceptibility/Vulnerability | 0 | 1 | 0.86 |
|  | General Attitudes & Beliefs | 0 | 1 | 0.81 |
| 8.2 Behaviour Substitution | Needs | 0 | 1 | 1 |
|  | Social/Professional Role & Identity | 1 | 0.30 | 0.95 |
|  | Social Learning / Imitation | 0 | 1 | 0.95 |
|  | Social Influences | 0 | 1 | 0.89 |
|  | Emotion | 0 | 1 | 0.89 |
|  | Norms | 0 | 1 | 0.89 |
|  | Subjective Norms | 0 | 1 | 0.89 |
|  | General Attitudes & Beliefs | 0 | 1 | 0.89 |
|  | Self-Image | 0 | 1 | 0.84 |
| 8.3 Habit Formation | Perceived Susceptibility/Vulnerability | 0 | 1 | 0.9 |
|  | Needs | 0 | 1 | 0.85 |
|  | General Attitudes & Beliefs | 0 | 1 | 0.85 |
|  | Knowledge | 0 | 1 | 0.8 |
|  | Optimism | 0 | 1 | 0.8 |
|  | Norms | 0 | 1 | 0.8 |
|  | Values | 0 | 1 | 0.8 |
|  | Feedback Processes | 0 | 1 | 0.8 |
| 8.4 Habit Reversal | Needs | 0 | 1 | 0.84 |
| 8.6 Generalisation of the Target Behaviour | Needs | 0 | 1 | 0.89 |
|  | Perceived Susceptibility/Vulnerability | 0 | 1 | 0.84 |
| 8.7 Graded Tasks | Social Influences | 0 | 1 | 1 |
|  | Norms | 0 | 1 | 1 |
|  | Needs | 0 | 1 | 1 |
|  | Subjective Norms | 0 | 1 | 0.95 |
|  | Values | 0 | 1 | 0.95 |
|  | Social/Professional Role & Identity | 0 | 1 | 0.86 |
|  | Social Learning / Imitation | 0 | 1 | 0.86 |
|  | Perceived Susceptibility/Vulnerability | 0 | 1 | 0.86 |
|  | Knowledge | 1 | 0.92 | 0.81 |
|  | Behavioural Cueing | 2 | 0.22 | 0.81 |
| 9.1 Credible Source | Skills | 4 | 0.77 | 0.89 |
|  | Needs | 0 | 1 | 0.89 |
|  | Reinforcement | 1 | 0.63 | 0.84 |
| 9.2 Pros and Cons | Social Learning / Imitation | 0 | 1 | 1 |
|  | Skills | 0 | 1 | 0.9 |
|  | Behavioural Cueing | 0 | 1 | 0.86 |
|  | Social/Professional Role & Identity | 0 | 1 | 0.81 |
|  | Self-Image | 0 | 1 | 0.81 |
|  | Needs | 1 | 0.11 | 0.81 |
| 9.3 Comparative Imagining of Future Outcomes | Skills | 0 | 1 | 1 |
|  | Social Learning / Imitation | 0 | 1 | 0.95 |
|  | Reinforcement | 0 | 1 | 0.9 |
|  | Norms | 0 | 1 | 0.9 |
|  | Environment | 0 | 1 | 0.86 |
|  | Social Influences | 0 | 1 | 0.86 |
|  | Social/Professional Role & Identity | 0 | 1 | 0.81 |
| 10.1 Material Incentive (behaviour) | Social Learning / Imitation | 0 | 1 | 0.84 |
| 10.2 Material Reward (behaviour) | Social/Professional Role & Identity | 0 | 1 | 0.95 |
|  | Social Learning / Imitation | 0 | 1 | 0.95 |
|  | Perceived Susceptibility/Vulnerability | 0 | 1 | 0.95 |
|  | Knowledge | 1 | 0.23 | 0.89 |
|  | Skills | 0 | 1 | 0.89 |
|  | Memory, Attention, & Decision Processes | 0 | 1 | 0.89 |
|  | Social Influences | 1 | 0.19 | 0.89 |
|  | Norms | 0 | 1 | 0.89 |
|  | Subjective Norms | 0 | 1 | 0.89 |
|  | Self-Image | 0 | 1 | 0.89 |
|  | Optimism | 0 | 1 | 0.84 |
| 10.3 Non-specific Reward | Social/Professional Role & Identity | 0 | 1 | 0.95 |
|  | Subjective Norms | 0 | 1 | 0.95 |
|  | Self-image | 1 | 0.055 | 0.95 |
|  | Needs | 0 | 1 | 0.95 |
|  | Values | 0 | 1 | 0.95 |
|  | Social Learning / Imitation | 0 | 1 | 0.95 |
|  | Perceived Susceptibility/Vulnerability | 0 | 1 | 0.95 |
|  | Optimism | 0 | 1 | 0.89 |
|  | Social Influences | 0 | 1 | 0.89 |
|  | Behavioural Regulation | 0 | 1 | 0.89 |
|  | Norms | 0 | 1 | 0.89 |
|  | Knowledge | 0 | 1 | 0.84 |
|  | Skills | 0 | 1 | 0.84 |
|  | Feedback Processes | 0 | 1 | 0.84 |
|  | General Attitudes & Beliefs | 0 | 1 | 0.84 |
| 10.4 Social reward | Perceived Susceptibility/Vulnerability | 0 | 1 | 1 |
|  | Skills | 2 | 0.80 | 0.95 |
|  | Memory, Attention, & Decision Processes | 2 | 0.27 | 0.9 |
|  | Social Learning / Imitation | 0 | 1 | 0.9 |
|  | Knowledge | 0 | 1 | 0.86 |
|  | Needs | 0 | 1 | 0.86 |
|  | General Attitudes & Beliefs | 0 | 1 | 0.86 |
| 10.6 Non-specific Incentive | Skills | N/A | N/A | 0.95 |
|  | Social/Professional Role & Identity | N/A | N/A | 0.95 |
|  | Social Influences | N/A | N/A | 0.95 |
|  | Social Learning / Imitation | N/A | N/A | 0.95 |
|  | Perceived Susceptibility/Vulnerability | N/A | N/A | 0.95 |
|  | Knowledge | N/A | N/A | 0.89 |
|  | Optimism | N/A | N/A | 0.89 |
|  | Norms | N/A | N/A | 0.89 |
|  | Subjective Norms | N/A | N/A | 0.89 |
|  | Self-Image | N/A | N/A | 0.89 |
|  | Beliefs about Capabilities | N/A | N/A | 0.84 |
|  | Values | N/A | N/A | 0.84 |
|  | Feedback Processes | N/A | N/A | 0.84 |
| 10.8 Incentive (outcome) | Skills | N/A | N/A | 1 |
|  | Perceived Susceptibility/Vulnerability | N/A | N/A | 1 |
|  | Social/Professional Role & Identity | N/A | N/A | 0.95 |
|  | Self -Image | N/A | N/A | 0.95 |
|  | Social Learning / Imitation | N/A | N/A | 0.95 |
|  | Subjective Norms | N/A | N/A | 0.9 |
|  | Social Influences | N/A | N/A | 0.86 |
|  | Norms | N/A | N/A | 0.86 |
|  | Values | N/A | N/A | 0.86 |
|  | Beliefs about Capabilities | N/A | N/A | 0.81 |
|  | Memory, Attention, & Decision Processes | N/A | N/A | 0.81 |
|  | General Attitudes & Beliefs | N/A | N/A | 0.81 |
| 10.10 Reward (outcome) | Skills | N/A | N/A | 1 |
|  | Social/Professional Role & Identity | N/A | N/A | 1 |
|  | Memory, Attention, & Decision Processes | N/A | N/A | 0.95 |
|  | Social Influences | N/A | N/A | 0.95 |
|  | Social Learning / Imitation | N/A | N/A | 0.95 |
|  | Perceived Susceptibility/Vulnerability | N/A | N/A | 0.95 |
|  | Knowledge | N/A | N/A | 0.9 |
|  | Norms | N/A | N/A | 0.9 |
|  | Self-Image | N/A | N/A | 0.9 |
|  | Subjective Norms | N/A | N/A | 0.86 |
|  | General Attitudes & Beliefs | N/A | N/A | 0.81 |
| 11.1 Pharmacological Support | Skills | 1 | 0.13 | 0.95 |
|  | Subjective Norms | 0 | 1 | 0.95 |
|  | Values | 0 | 1 | 0.95 |
|  | Feedback Processes | 0 | 1 | 0.95 |
|  | Social Learning / Imitation | 0 | 1 | 0.95 |
|  | Social/Professional Role & Identity | 0 | 1 | 0.89 |
|  | Norms | 0 | 1 | 0.89 |
|  | Perceived Susceptibility/Vulnerability | 0 | 1 | 0.84 |
| 11.2 Reduce Negative Emotions | Subjective Norms | 0 | 1 | 0.95 |
|  | Values | 0 | 1 | 0.95 |
|  | Social/Professional Role & Identity | 0 | 1 | 0.9 |
|  | Norms | 0 | 1 | 0.9 |
|  | Feedback Processes | 0 | 1 | 0.9 |
|  | Social Learning / Imitation | 0 | 1 | 0.9 |
|  | Knowledge | 0 | 1 | 0.86 |
|  | Behavioural Cueing | 0 | 1 | 0.86 |
| 11.3 Conserving Mental Resources | Social/Professional Role & Identity | 0 | 1 | 1 |
|  | Norms | 0 | 1 | 1 |
|  | Subjective Norms | 0 | 1 | 1 |
|  | Perceived Susceptibility/Vulnerability | 0 | 1 | 1 |
|  | Social Influences | 0 | 1 | 0.95 |
|  | Needs | 0 | 1 | 0.95 |
|  | Values | 0 | 1 | 0.95 |
|  | Feedback Processes | 0 | 1 | 0.95 |
|  | Social Learning / Imitation | 0 | 1 | 0.95 |
|  | Self-Image | 0 | 1 | 0.9 |
|  | Reinforcement | 0 | 1 | 0.86 |
|  | Emotion | 0 | 1 | 0.86 |
|  | General Attitudes & Beliefs | 0 | 1 | 0.86 |
|  | Beliefs about Consequences | 0 | 1 | 0.81 |
| 12.1 Restructuring the Physical Environment | Social/Professional Role & Identity | 1 | 0.36 | 0.84 |
|  | Optimism | 1 | 0.22 | 0.84 |
|  | Self-Image | 0 | 1 | 0.84 |
|  | Needs | 0 | 1 | 0.84 |
| 12.2 Restructuring the Social Environment | Skills | 0 | 1 | 0.9 |
|  | Optimism | 0 | 1 | 0.9 |
|  | Needs | 0 | 1 | 0.9 |
|  | Beliefs about Consequences | 0 | 1 | 0.85 |
|  | Feedback Processes | 0 | 1 | 0.85 |
|  | Attitude towards the Behaviour | 0 | 1 | 0.8 |
| 12.3 Avoidance / Reducing Exposure to Cues for the Behaviour | Feedback Processes | 0 | 1 | 0.95 |
|  | Social Learning / Imitation | 0 | 1 | 0.95 |
|  | Social/Professional Role & Identity | 0 | 1 | 0.89 |
|  | Subjective Norms | 1 | 0.38 | 0.89 |
|  | General Attitudes & Beliefs | 0 | 1 | 0.89 |
|  | Knowledge | 0 | 1 | 0.84 |
|  | Norms | 0 | 1 | 0.84 |
|  | Self-Image | 0 | 1 | 0.84 |
| 12.5 Adding Objects to the Environment | Social Learning / Imitation | 0 | 1 | 1 |
|  | Skills | 0 | 1 | 0.86 |
|  | Social/Professional Role & Identity | 0 | 1 | 0.86 |
|  | Beliefs about Consequences | 0 | 1 | 0.86 |
|  | Norms | 0 | 1 | 0.86 |
|  | Values | 0 | 1 | 0.86 |
|  | Optimism | 0 | 1 | 0.81 |
|  | Social Influences | 0 | 1 | 0.81 |
|  | Self-Image | 0 | 1 | 0.81 |
|  | Feedback Processes | 0 | 1 | 0.81 |
|  | Perceived Susceptibility/Vulnerability | 0 | 1 | 0.81 |
| 12.6 Body Changes | Norms | 0 | 1 | 1 |
|  | Subjective Norms | 0 | 1 | 0.95 |
|  | Social Learning / Imitation | 0 | 1 | 0.95 |
|  | Knowledge | 0 | 1 | 0.9 |
|  | Values | 0 | 1 | 0.9 |
|  | Social/Professional Role & Identity | 0 | 1 | 0.86 |
|  | Social Influences | 0 | 1 | 0.86 |
|  | Memory, Attention, & Decision Processes | 0 | 1 | 0.81 |
|  | Feedback Processes | 0 | 1 | 0.81 |
| 13.1 Identification of Self as Role Model | Perceived Susceptibility/Vulnerability | 0 | 1 | 0.9 |
|  | Skills | 2 | 0.44 | 0.8 |
|  | Optimism | 1 | 0.16 | 0.8 |
|  | Needs | 0 | 1 | 0.8 |
| 13.2 Framing / Reframing | Skills | 3 | 0.67 | 0.95 |
|  | Needs | 0 | 1 | 0.95 |
|  | Social/Professional Role & Identity | 0 | 1 | 0.9 |
|  | Social Learning / Imitation | 0 | 1 | 0.9 |
|  | Reinforcement | 0 | 1 | 0.86 |
|  | Norms | 0 | 1 | 0.86 |
|  | Behavioural Cueing | 1 | 0.587 | 0.86 |
|  | Environmental Context & Resources | 0 | 1 | 0.81 |
|  | Feedback Processes | 0 | 1 | 0.81 |
| 14.1 Behaviour cost | Skills | N/A | N/A | 0.84 |
|  | Social/Professional Role & Identity | N/A | N/A | 0.84 |
|  | Subjective Norms | 0 | 1 | 0.84 |
| 15.1 Verbal Persuasion about Capability | Needs | 0 | 1 | 0.95 |
|  | Social/Professional Role & Identity | 0 | 1 | 0.9 |
|  | Memory, Attention, & Decision Processes | 0 | 1 | 0.9 |
|  | Social Learning / Imitation | 0 | 1 | 0.9 |
|  | Norms | 0 | 1 | 0.86 |
|  | Values | 0 | 1 | 0.86 |
|  | General Attitudes & Beliefs | 0 | 1 | 0.86 |
|  | Perceived Susceptibility/Vulnerability | 0 | 1 | 0.86 |
|  | Feedback Processes | 0 | 1 | 0.81 |
| 15.4 Self-talk | Knowledge | 0 | 1 | 0.95 |
|  | Environment | 0 | 1 | 0.95 |
|  | Norms | 0 | 1 | 0.95 |
|  | Perceived Susceptibility/Vulnerability | 0 | 1 | 0.95 |
|  | Social Influences | 0 | 1 | 0.89 |
|  | Subjective Norms | 0 | 1 | 0.89 |
|  | Values | 0 | 1 | 0.89 |
|  | Feedback Processes | 0 | 1 | 0.89 |
|  | Social Learning / Imitation | 0 | 1 | 0.89 |
|  | Social/Professional Role & Identity | 0 | 1 | 0.84 |
|  | Reinforcement | 0 | 1 | 0.84 |
|  | Needs | 0 | 1 | 0.84 |
| 16.3 Vicarious Consequences | Skills | 1 | 0.69 | 0.95 |
|  | Needs | 0 | 1 | 0.9 |
|  | Perceived Susceptibility/Vulnerability | 0 | 1 | 0.85 |

*Table S6:* Links agreed to be absent in reconciliation study (i.e. 80% or more of experts in the current study agreed there is no link)

|  | | **Literature Synthesis Study** | | **Expert Consensus Study** | | | | **Reconciliation Study** | | |
| --- | --- | --- | --- | --- | --- | --- | --- | --- | --- | --- |
| **Behaviour Change Technique** | **Mechanism of Action** | Frequency (number of papers) | *p* value | % Experts (Yes) | % Experts (Possibly) | % Experts (Don’t Know) | % Experts (No) | % Experts (Yes) | % Experts (Don’t Know) | % Experts  (No) |
| 2.1 Monitoring of Behaviour by Others without Feedback | Needs | 1 | 0.019 | 0 | 11 | 0 | 89 | 6.25 | 12.5 | 81.25 |
| 12.3 Avoidance / Reducing Exposure to Cues for the Behaviour | Needs | 1 | 0.027 | 5 | 5 | 0 | 90 | 0 | 0 | 100 |
| 2.2 Feedback on Behaviour | Subjective Norms | 19 | <0.001 | 5 | 16 | 11 | 68 | 6.25 | 12.5 | 81.25 |
| 4.2 Information about Antecedents | Skills | 3 | 0.10 | 9 | 5 | 0 | 86 | 0 | 0 | 100 |
| 10.3 Non-specific Reward | Self-Image | 1 | 0.06 | 5 | 0 | 0 | 95 | 0 | 12.5 | 87.5 |

*Table S7*: Predicting expert consensus from literature study for each MoA for ‘YES’ and ‘NO’ responses (Multilevel modelling: intercept (Int) and slope (Slp); with and without p=1 BCTs)

|  | ‘YES’ All | | | ‘YES’, p=1 BCTs removed | | | ‘NO’ All | | | ‘NO’, p=1 BCTs removed | | |
| --- | --- | --- | --- | --- | --- | --- | --- | --- | --- | --- | --- | --- |
| **MoA** | n | Int | Slp | N | Int | Slp | n | Int | Slp | n | Int | Slp |
| Knowledge | 56 | .48 | -.37 | 29 | .52 | -.50 | 56 | .30 | .36 | 29 | .26 | .43 |
| Skills | 56 | .35 | -.29 | 36 | .40 | -.41 | 56 | .47 | .35 | 36 | .39 | .54 |
| Social/professional role & identity | 56 | .24 | -.19 | 19 | .32 | -.40 | 56 | .46 | .35 | 19 | .39 | .52 |
| Beliefs about capabilities | 56 | .58 | -.36 | 51 | .60 | -.43 | 56 | .18 | .29 | 51 | .14 | .38 |
| Optimism | 56 | .22 | -.15 | 19 | .29 | -.38 | 56 | .38 | .29 | 19 | .36 | .49 |
| Beliefs about consequences | 56 | .63 | -.44 | 37 | .66 | -.55 | 56 | .19 | .35 | 37 | .20 | .37 |
| Reinforcement | 56 | .54 | -.33 | 20 | .56 | -.51 | 56 | .26 | .31 | 20 | .26 | .44 |
| Intentions | 56 | .59 | -.25 | 42 | .62 | -.33 | 56 | .14 | .22 | 42 | .08 | .34 |
| Goals | 56 | .61 | -.38 | 28 | .63 | -.53 | 56 | .19 | .29 | 28 | .18 | .37 |
| Memory, Attention and Decision Processes | 56 | .43 | -.33 | 28 | .48 | -.47 | 56 | .29 | .36 | 28 | .27 | .43 |
| Environmental context and resources | 56 | .71 | -.54 | 22 | .71 | -.62 | 56 | .20 | .40 | 22 | .22 | .37 |
| Social influences | 56 | .60 | -.50 | 28 | .62 | -.53 | 56 | .27 | .43 | 28 | .26 | .40 |
| Emotion | 56 | .42 | -.29 | 20 | .46 | -.47 | 56 | .27 | .30 | 20 | .29 | .43 |
| Behavioral regulation | 56 | .59 | -.36 | 36 | .61 | -.41 | 56 | .20 | .32 | 33 | .15 | .38 |
| Norms | - | - | - | - | - | - | - | - | - | - | - | - |
| Subjective norms | 56 | .41 | -.34 | 25 | .46 | -.44 | 56 | .36 | .40 | 25 | .30 | .46 |
| Attitude toward the behavior | 56 | .57 | -.43 | 27 | .59 | -.54 | 56 | .20 | .35 | 27 | .21 | .37 |
| Motivation | 56 | .76 | -.40 | 36 | .77 | -.43 | 56 | .04 | .27 | 36 | .03 | .29 |
| Self-image | 56 | .34 | -.17 | 15 | .39 | -.41 | 56 | .30 | .27 | 15 | .30 | .47 |
| Needs | 56 | .15 | -.12 | 6 | .21 | -.36 | 56 | .57 | .21 | 6 | .55 | .66 |
| Values | 56 | .24 | -.13 | 4 | .27 | -.38 | 56 | .39 | .29 | 4 | .42 | .55 |
| Feedback processes | 56 | .60 | -.42 | 12 | .61 | -.52 | 56 | .28 | .36 | 12 | .24 | .42 |
| Social learning/imitation | 56 | .38 | -.33 | 10 | .43 | -.46 | 56 | .47 | .36 | 10 | .41 | .54 |
| Behavioral cueing | 56 | .60 | -.37 | 21 | .61 | -.54 | 56 | .23 | .29 | 21 | .26 | .42 |
| General attitudes/beliefs | 56 | .46 | -.34 | 3 | .49 | -.47 | 56 | .28 | .34 | 3 | .27 | .43 |
| Perceived susceptibility/vulnerability | 56 | .37 | -.31 | 11 | .43 | -.49 | 56 | .41 | .36 | 11 | .38 | .50 |

*Table S8*: Multilevel model predicting judgements of expert consensus from the literature synthesis with and without p=1: estimated beta weights (standard error) for fixed effects and variances (standard error) for random effects.

**____________________________________________________________________________­­­­­­­­­­­­­­­­­­­­­­­­­­­­­­­­­**

|  | “YES” Expert Consensus | | “NO” Expert Consensus | |  |
| --- | --- | --- | --- | --- | --- |
|  | All data | p=1 omitted | All data | p=1 omitted |  |
| Fixed effects | |  |  |  |  |
| Intercept | 0.474(0.037)*** | 0.510 (0.036)*** | 0.295(0.030)*** | 0.270(0.029)*** |  |
| Literature | -0.325(0.031)*** | -0.464(0.042)*** | 0.327(0.24)*** | 0.439(0.035)*** |  |
| Random effects |  |  |  |  |  |
| *Level 2: Mechanism of Action* |  |  |  |  |  |
| Intercept | 0.029(0.01)*** | 0.025(0.009)* | 0.017(0.007)* | 0.014(0.006) |  |
| Literature | 0.016 (0.007)* | 0.014 (0.011) | 0.006(0.004) | 0.005(0.007) |  |
| *Level 1: BCT* |  |  |  |  |  |
| Intercept | 0.045(0.002)*** | 0.059(0.004)*** | 0.056(0.002)*** | 0.058(0.003)*** |  |
|  |  |  |  |  |  |

*p<.01, ** p<.01, *** p<.001
