# Supplementary material for: Development of an online tool for linking behavior change techniques and mechanisms of action based on triangulation of findings from literature synthesis and expert consensus
Source: Transl Behav Med. 2020 Aug 4;11(5):1049–65. doi: 10.1093/tbm/ibaa050 (PMC8158171; doi:10.1093/tbm/ibaa050)
Supplement: ibaa050_suppl_Supplementary-File-2 [file ibaa050_suppl_supplementary-file-2.docx]

**Supplement 2.** Regression of expert consensus on links from systematic review for individual MoAs.

This supplement shows the individual data points and regressions lines from the multilevel models regressing expert consensus on links established from the sytematic literature review. The regression lines are from the analyses excluding the cases where a BCT was detected but no link to a MoA was proposed in the paper (i.e., when p=1 for the link). Figures 1a-25a show the results for occasions when the experts indicated whether they thought there was a link (YES responses) and Figures 1b-25b when they thought there was no link (NO responses). Twenty five of the 26 possible MoAs were found in the literature review. The data points show the identifier for each BCT. The BCTs with p=1 in the literature review are shown, but as stated, were not included in the analyses. It should be recalled that the regression lines are derived from a multilevel analysis and when there are very few data points (e.g., Figures 19 (Needs) and 24 (General attitudes)) the regression lines will be pulled towards the overall regression line because of residual shrinkage. Regression lines based solely on the data for the specific MoA are unlikely to be accurate when numbers are so low.

**Index**

Table 1. Behaviour Change Technique Taxonomy v1 Identifier Codes

Figure 1a. Relation between literature and expert consensus when links between BCTs and MoA **“Knowledge”** have been proposed

Figure 1b. Relation between literature and expert consensus when NO links between BCTs and MoA **“Knowledge”** have been proposed

Figure 2a. Relation between literature and expert consensus when links between BCTs and MoA **“Skills”** have been proposed.

Figure 2b. Relation between literature and expert consensus when NO links between BCTs and MoA **“Skills”** have been proposed.

Figure 3a. Relation between literature and expert consensus when links between BCTs and MoA **“Social/professional role and identity”** have been proposed.

Figure 3b. Relation between literature and expert consensus when NO links between BCTs and MoA **“Social/professional role and identity”** have been proposed.

Figure 4a. Relation between literature and expert consensus when links between BCTs and MoA **“Beliefs about Capabilities”** have been proposed.

Figure 4b. Relation between literature and expert consensus when NO links between BCTs and MoA **“Beliefs about Capabilities”** have been proposed.

Figure 5a. Relation between literature and expert consensus when links between BCTs and MoA **“Optimism”** have been proposed.

Top of Form

Bottom of Form

Figure 5b. Relation between literature and expert consensus when NO links between BCTs and MoA **“Optimism”** have been proposed.

Figure 6a. Relation between literature and expert consensus when links between BCTs and MoA **“Beliefs about consequences”** have been proposed.

Figure 6b. Relation between literature and expert consensus when NO links between BCTs and MoA **“Beliefs about consequences”** have been proposed.

Figure 7a. Relation between literature and expert consensus when links between BCTs and MoA **“Reinforcement”** have been proposed.

Figure 7b. Relation between literature and expert consensus when NO links between BCTs and MoA **“Reinforcement”** have been proposed.

Figure 8a. Relation between literature and expert consensus when links between BCTs and MoA **“Intentions”** have been proposed.

Figure 8b. Relation between literature and expert consensus when NO links between BCTs and MoA **“Intentions”** have been proposed.

Figure 9a. Relation between literature and expert consensus when links between BCTs and MoA **“Goals”** have been proposed.

Figure 9b Relation between literature and expert consensus when NO links between BCTs and MoA **“Goals”** have been proposed.

Figure 10a. Relation between literature and expert consensus when links between BCTs and MoA **“Memory, attention and decision processes”** have been proposed

Figure 10b. Relation between literature and expert consensus when NO links between BCTs and MoA **“Memory, attention and decision processes”** have been proposed.

Figure 11a. Relation between literature and expert consensus when links between BCTs and MoA **“Environmental context and resources”** have been proposed.

Figure 11b. Relation between literature and expert consensus when NO links between BCTs and MoA **“Environmental context and resources”** have been proposed.

Figure 12a. Relation between literature and expert consensus when links between BCTs and MoA **“Social influence”** have been proposed.

Figure 12b. Relation between literature and expert consensus when NO links between BCTs and MoA **“Social influence”** have been proposed.

Figure 13a. Relation between literature and expert consensus when links between BCTs and MoA **“Emotion”** have been proposed.

Figure 13b. Relation between literature and expert consensus when NO links between BCTs and MoA **“Emotion”** have been proposed

Figure 14a. Relation between literature and expert consensus when links between BCTs and MoA **“Behavioural regulation”** have been proposed.

Figure 14b. Relation between literature and expert consensus when NO links between BCTs and MoA **“Behavioural regulation”** have been proposed.

Figure 15a. Relation between literature and expert consensus when links between BCTs and MoA **“Subjective knowledge”** have been proposed.

Figure 15b. Relation between literature and expert consensus when NO links between BCTs and MoA **“Subjective knowledge”** have been proposed.

Figure 16a. Relation between literature and expert consensus when links between BCTs and MoA **“Attitude towards the behaviour”** have been proposed.

Figure 16b. Relation between literature and expert consensus when NO links between BCTs and MoA **“Attitude towards the behaviour”** have been proposed.

Figure 17a. Relation between literature and expert consensus when links between BCTs and MoA **“Motivation”** have been proposed.

Figure 17b. Relation between literature and expert consensus when NO links between BCTs and MoA **“Motivation”** have been proposed.

Figure 18a. Relation between literature and expert consensus when links between BCTs and MoA **“Self-image”** have been proposed.

Figure 18b. Relation between literature and expert consensus when NO links between BCTs and MoA **“Self-image”** have been proposed.

Figure 19a. Relation between literature and expert consensus when links between BCTs and MoA **“Needs”** have been proposed.

Figure 19b. Relation between literature and expert consensus when NO links between BCTs and MoA **“Needs”** have been proposed.

Figure 20a. Relation between literature and expert consensus when links between BCTs and MoA **“Values”** have been proposed.

Figure 20b. Relation between literature and expert consensus when NO links between BCTs and MoA **“Values”** have been proposed.

Figure 21a. Relation between literature and expert consensus when links between BCTs and MoA **“Feedback processes”** have been proposed.

Figure 21ab. Relation between literature and expert consensus when NO links between BCTs and MoA **“Feedback processes”** have been proposed.

Figure 22a. Relation between literature and expert consensus when links between BCTs and MoA **“Social learning/imitation”** have been proposed.

Figure 22b. Relation between literature and expert consensus when NO links between BCTs and MoA **“Social learning/imitation”** have been proposed.

Figure 23a. Relation between literature and expert consensus when links between BCTs and MoA **“Behavioral cueing”** have been proposed.

Figure 23b. Relation between literature and expert consensus when NO links between BCTs and MoA **“Behavioral cueing”** have been proposed.

Figure 24a. Relation between literature and expert consensus when links between BCTs and MoA **“General attitudes/beliefs”** have been proposed.

Figure 24b. Relation between literature and expert consensus when NO links between BCTs and MoA **“General attitudes/beliefs”** have been proposed.

Figure 25a. Relation between literature and expert consensus when links between BCTs and MoA **“Perceived susceptibility/vulnerability”** have been proposed.

Figure 25b. Relation between literature and expert consensus when NO links between BCTs and MoA  **“Perceived susceptibility/vulnerability”** have been proposed.

**Table 1.**  Behaviour Change Technique Taxonomy v1 (BCTTv1) Identifier Codes

| BCTTv1 CODE | BCT NAME |
| --- | --- |
| 1.1 | Goal setting (behaviour) |
| 1.2 | Problem solving |
| 1.3 | Goal setting (outcome) |
| 1.4 | Action planning |
| 1.5 | Review behaviour goals |
| 1.6 | Discrepancy between current behaviour and goal |
| 1.7 | Review outcome goals |
| 1.8 | Behavioural contract |
| 1.9 | Commitment |
| 2.1 | Monitoring of behaviour by others without feedback |
| 2.2 | Feedback on behaviour |
| 2.3 | Self-monitoring of behaviour |
| 2.4 | Self-monitoring of outcomes of behaviour |
| 2.5 | Monitoring of outcomes of behaviour without feedback |
| 2.6 | Biofeedback |
| 2.7 | Feedback on outcomes of behaviour |
| 3.1 | Social support (unspecified) |
| 3.2 | Social support (practical) |
| 3.3 | Social support (emotional) |
| 4.1 | Instruction on how to perform the behaviour |
| 4.2 | Information about Antecedents |
| 4.3 | Re-attribution |
| 4.4 | Behavioural experiments |
| 5.1 | Information about health consequences |
| 5.2 | Salience of consequences |
| 5.3 | Information about social and environmental consequences |
| 5.4 | Monitoring of emotional consequences |
| 5.5 | Anticipated regret |
| 5.6 | Information about emotional consequences |
| 6.1 | Demonstration of the behaviour |
| 6.2 | Social comparison |
| 6.3 | Information about other's approval |
| 7.1 | Prompts/cues |
| 7.2 | Cue signalling reward |
| 7.3 | Reduce prompts/cues |
| 7.4 | Remove access to the reward |
| 7.5 | Remove aversive stimulus |
| 7.6 | Satiation |
| 7.7 | Exposure |
| 7.8 | Associative learning |
| 8.1 | Behavioural practice/rehearsal |
| 8.2 | Behaviour substitution |
| 8.3 | Habit formation |
| 8.4 | Habit reversal |
| 8.5 | Overcorrection |
| 8.6 | Generalisation of the target behaviour |
| 8.7 | Graded tasks |
| 9.1 | Credible source |
| 9.2 | Pros and cons |
| 9.3 | Comparative imagining of future outcomes |
| 10.1 | Material incentive (behaviour) |
| 10.2 | Material reward (behaviour) |
| 10.3 | Non-specific reward |
| 10.4 | Social reward |
| 10.5 | Social incentive |
| 10.6 | Non-specific incentive |
| 10.7 | Self-incentive |
| 10.8 | Incentive (outcome) |
| 10.9 | Self-reward |
| 10.10 | Reward (outcome) |
| 10.11 | Future punishment |
| 11.1 | Pharmacological support |
| 11.2 | Reduce negative emotions |
| 11.3 | Conserving mental resources |
| 11.4 | Paradoxical instructions |
| 12.1 | Restructuring the physical environment |
| 12.2 | Restructuring the social environment |
| 12.3 | Avoidance/reducing exposure to cues for the behaviour |
| 12.4 | Distraction |
| 12.5 | Adding objects to the environment |
| 12.6 | Body changes |
| 13.1 | Identification of self as role model |
| 13.2 | Framing/reframing |
| 13.3 | Incompatible beliefs |
| 13.4 | Valued self-identity |
| 13.5 | Identity associated with changed behaviour |
| 14.1 | Behaviour cost |
| 14.2 | Punishment |
| 14.3 | Remove reward |
| 14.4 | Reward approximation |
| 14.5 | Rewarding completion |
| 14.6 | Situation-specific reward |
| 14.7 | Reward incompatible behaviour |
| 14.8 | Reward alternative behaviour |
| 14.9 | Reduce reward frequency |
| 14.10 | Remove punishment |
| 15.1 | Verbal persuasion about capability |
| 15.2 | Mental rehearsal of successful performance |
| 15.3 | Focus on past success |
| 15.4 | Self-talk |
| 16.1 | Imaginary punishment |
| 16.2 | Imaginary reward |
| 16.3 | Vicarious consequences |


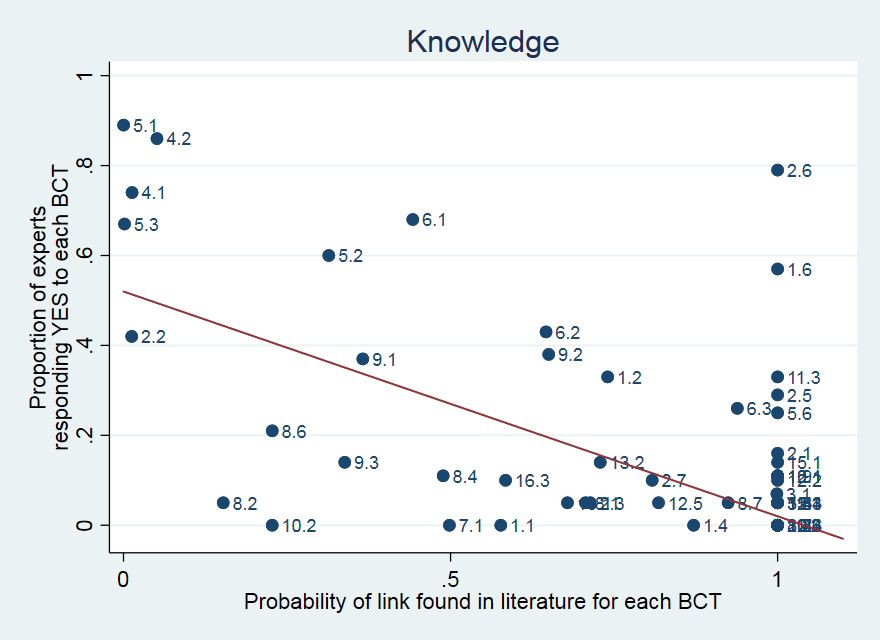


Figure 1a. Relation between literature and expert consensus when links between BCTs and MoA “Knowledge” have been proposed. Each dot represents one BCT (with BCTTv1 label). The line represents the prediction from the MLM omitting BCTs for which p=1 in the literature study (but the expert consensus values for such BCTs are shown).


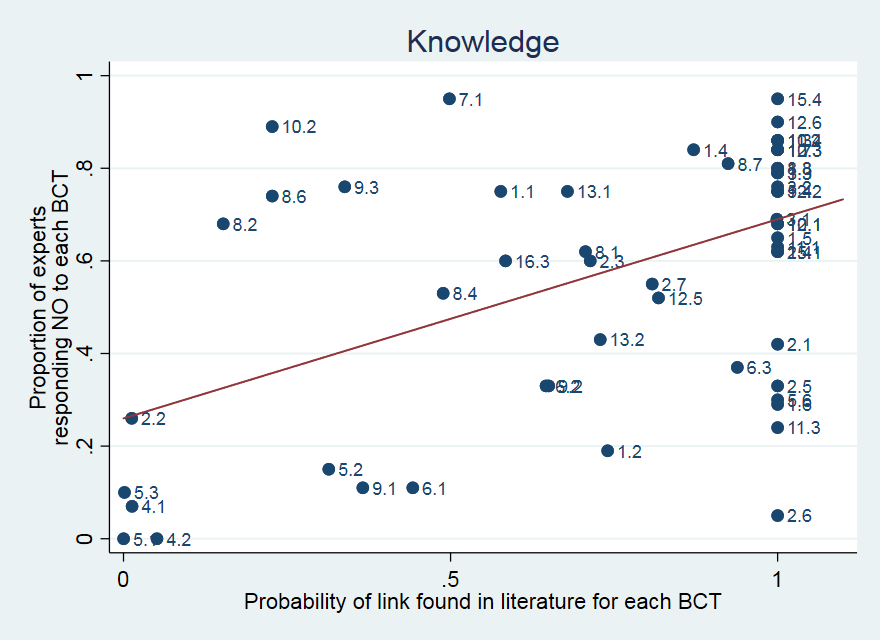


Figure 1b. Relation between literature and expert consensus when no links between BCTs and MoA “Knowledge” have been proposed. Each dot represents one BCT (with BCTTv1 label). The line represents the prediction from the MLM omitting BCTs for which p=1 in the literature study (but the expert consensus values for such BCTs are shown).


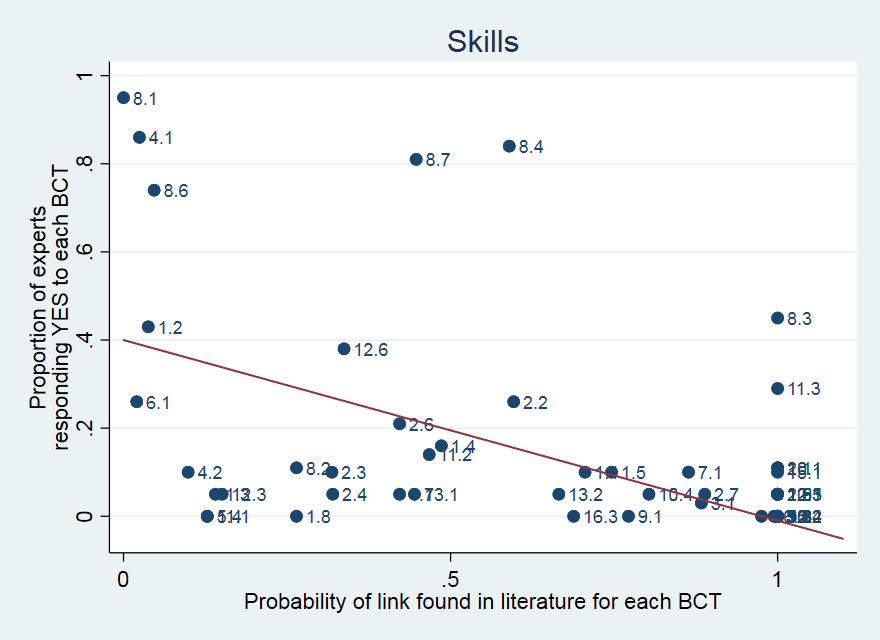


Figure 2a. Relation between literature and expert consensus when links between BCTs and MoA “Skills” have been proposed. Each dot represents one BCT (with BCTTv1 label). The line represents the prediction from the MLM omitting BCTs for which p=1 in the literature study (but the expert consensus values for such BCTs are shown).


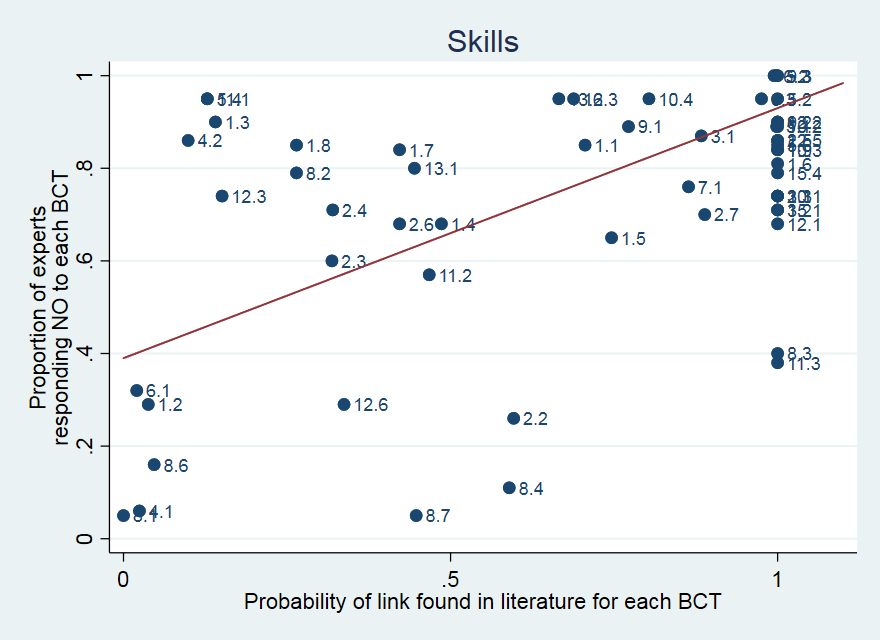


Figure 2b. Relation between literature and expert consensus when no links between BCTs and MoA “Skills” have been proposed. Each dot represents one BCT (with BCTTv1 label). The line represents the prediction from the MLM omitting BCTs for which p=1 in the literature study (but the expert consensus values for such BCTs are shown).


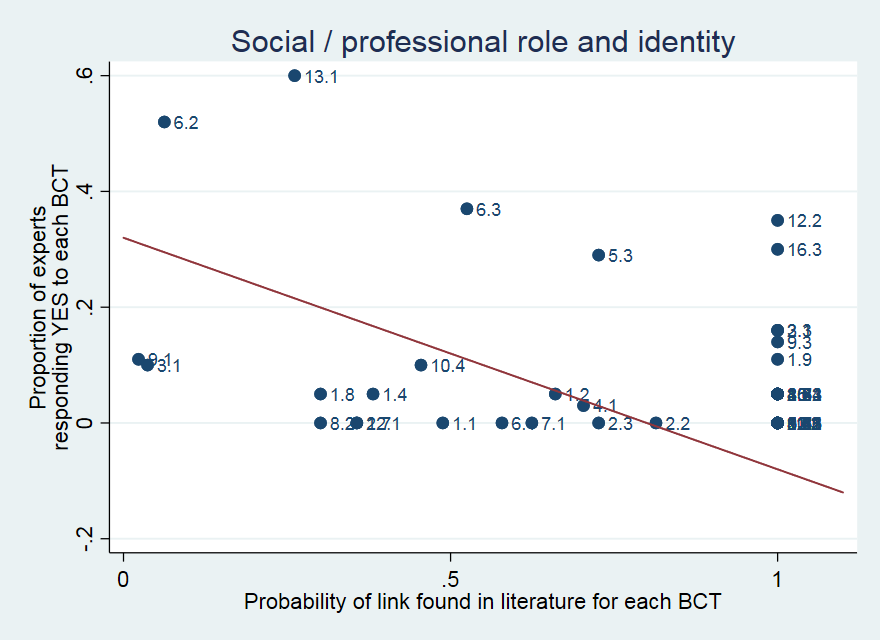


Figure 3a. Relation between literature and expert consensus when links between BCTs and MoA “Social/professional role and identity” have been proposed. Each dot represents one BCT (with BCTTv1 label). The line represents the prediction from the MLM omitting BCTs for which p=1 in the literature study (but the expert consensus values for such BCTs are shown).


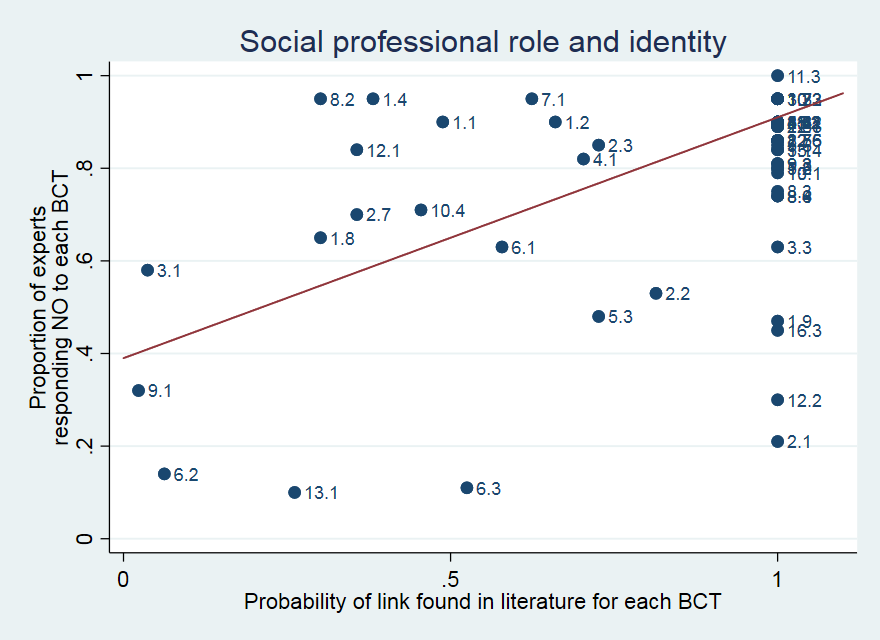


Figure 3b. Relation between literature and expert consensus when no links between BCTs and MoA “Social/professional role and identity” have been proposed. Each dot represents one BCT (with BCTTv1 label). The line represents the prediction from the MLM omitting BCTs for which p=1 in the literature study (but the expert consensus values for such BCTs are shown).


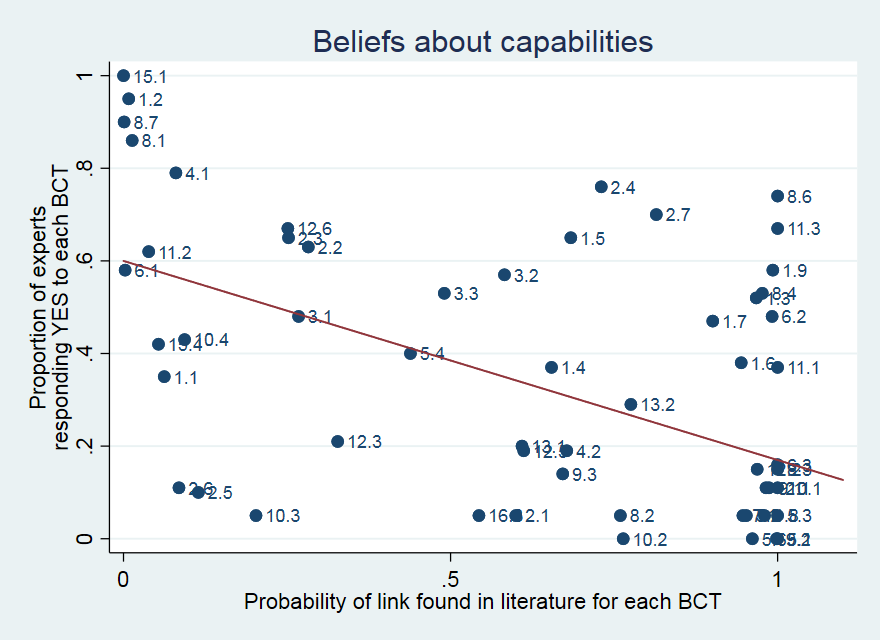


Figure 4a. Relation between literature and expert consensus when links between BCTs and MoA “Beliefs about Capabilities” have been proposed. Each dot represents one BCT (with BCTTv1 label). The line represents the prediction from the MLM omitting BCTs for which p=1 in the literature study (but the expert consensus values for such BCTs are shown).


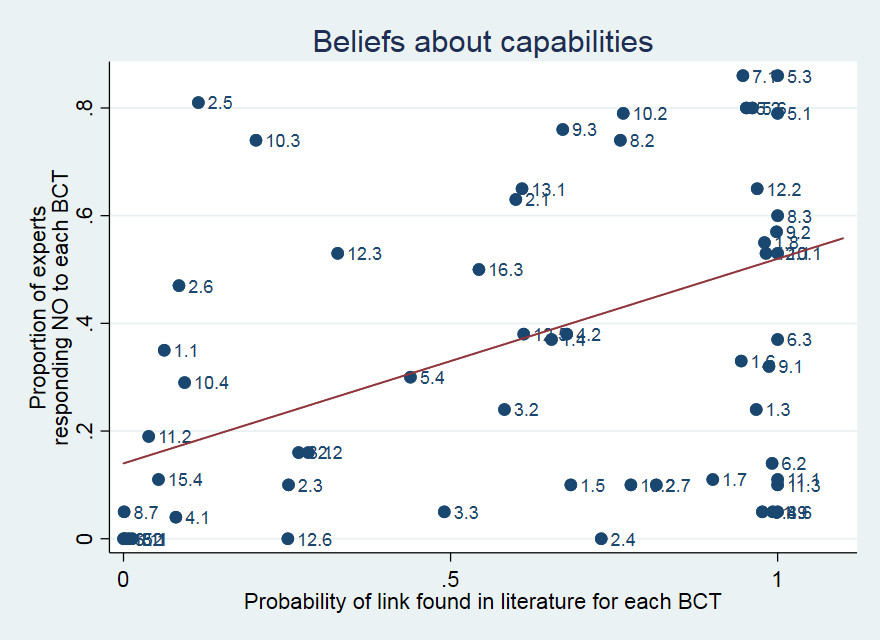


Figure 4b. Relation between literature and expert consensus when no links between BCTs and MoA “Beliefs about Capabilities” have been proposed. Each dot represents one BCT (with BCTTv1 label). The line represents the prediction from the MLM omitting BCTs for which p=1 in the literature study (but the expert consensus values for such BCTs are shown).


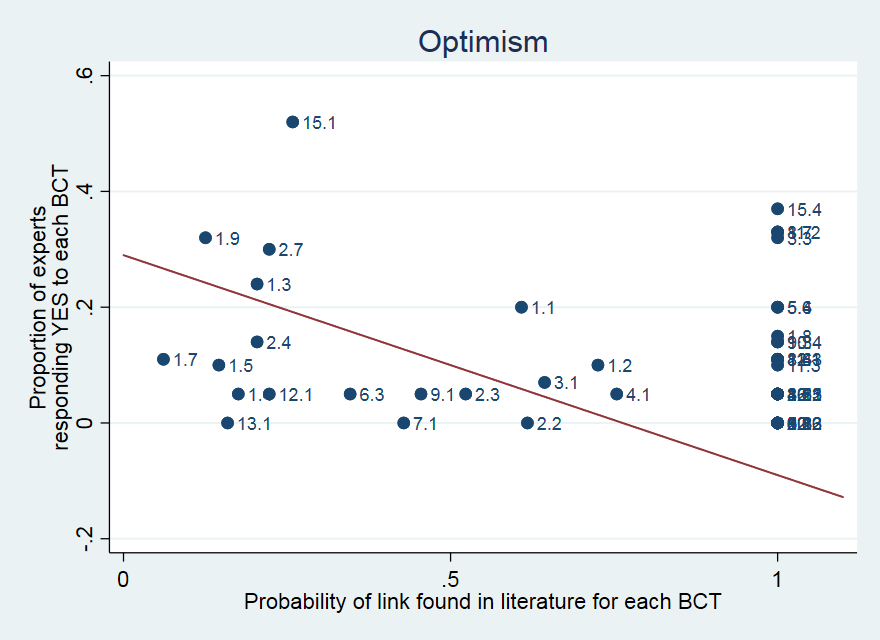


Figure 5a. Relation between literature and expert consensus when links between BCTs and MoA “Optimism” have been proposed. Each dot represents one BCT (with BCTTv1 label). The line represents the prediction from the MLM omitting BCTs for which p=1 in the literature study (but the expert consensus values for such BCTs are shown).

Top of Form

Bottom of Form


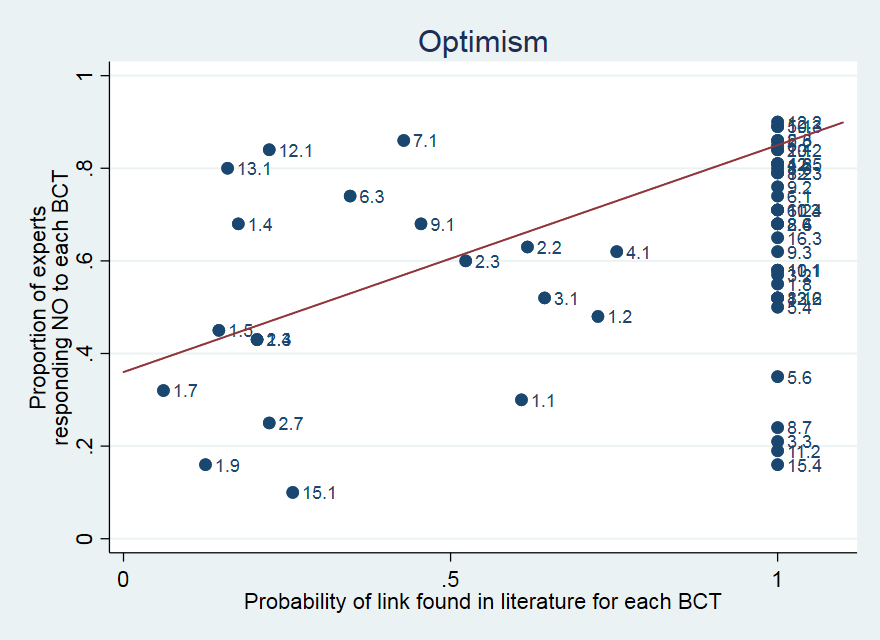


Figure 5b. Relation between literature and expert consensus when no links between BCTs and MoA “Optimism” have been proposed. Each dot represents one BCT (with BCTTv1 label). The line represents the prediction from the MLM omitting BCTs for which p=1 in the literature study (but the expert consensus values for such BCTs are shown).


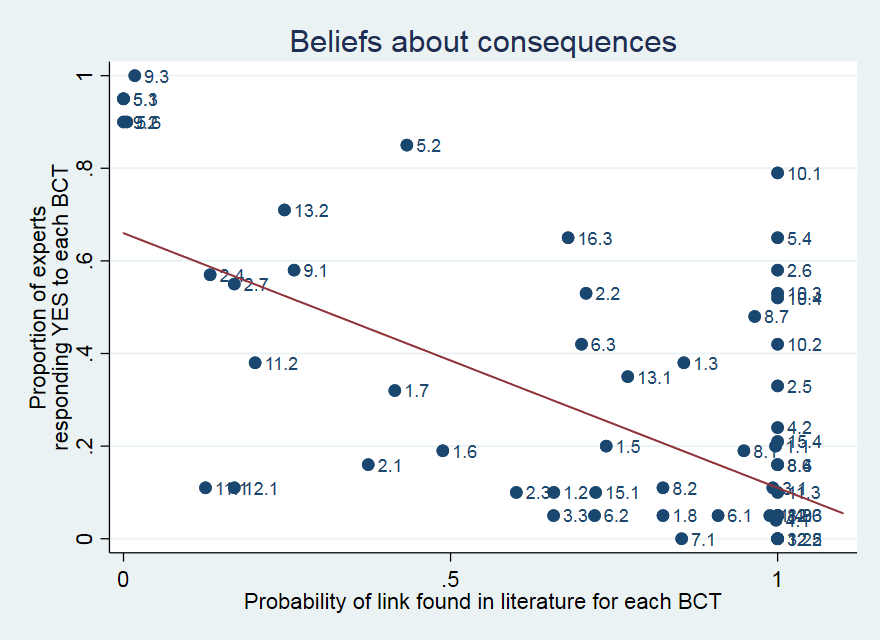


Figure 6a. Relation between literature and expert consensus when links between BCTs and MoA “Beliefs about consequences” have been proposed. Each dot represents one BCT (with BCTTv1 label). The line represents the prediction from the MLM omitting BCTs for which p=1 in the literature study (but the expert consensus values for such BCTs are shown).


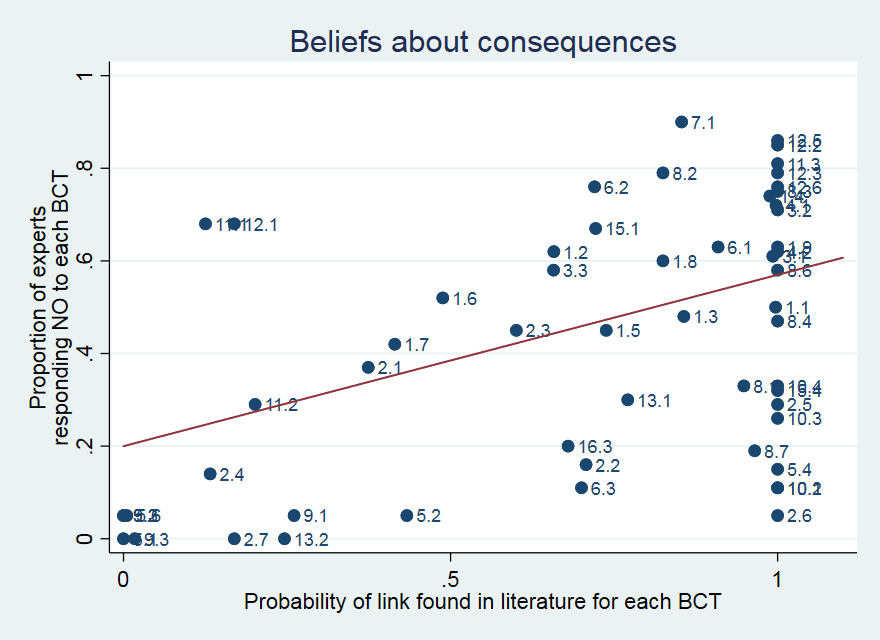


Figure 6a. Relation between literature and expert consensus when links between BCTs and MoA “Beliefs about consequences” have been proposed. Each dot represents one BCT (with BCTTv1 label). The line represents the prediction from the MLM omitting BCTs for which p=1 in the literature study (but the expert consensus values for such BCTs are shown).


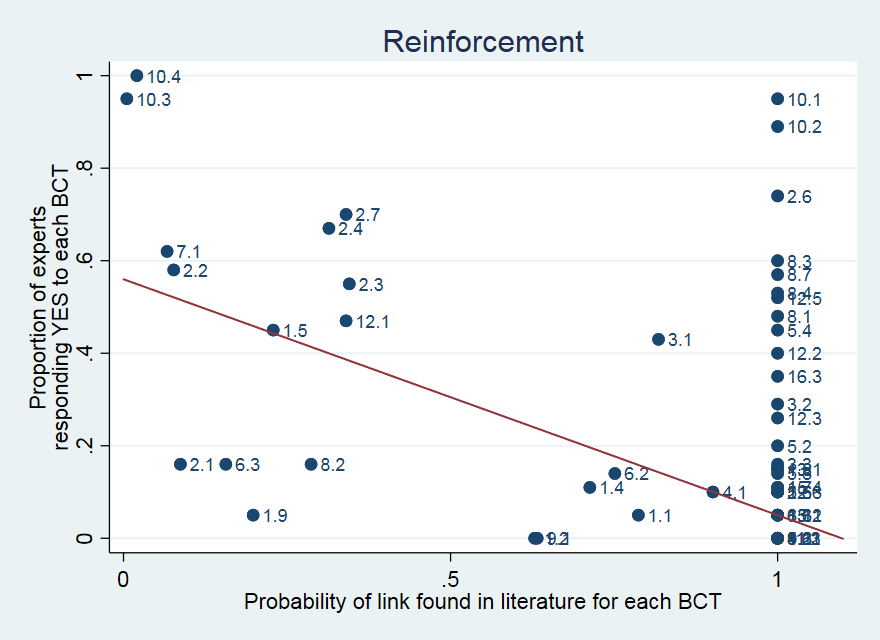


Figure 7a. Relation between literature and expert consensus when links between BCTs and MoA “Reinforcement” have been proposed. Each dot represents one BCT (with BCTTv1 label). The line represents the prediction from the MLM omitting BCTs for which p=1 in the literature study (but the expert consensus values for such BCTs are shown).


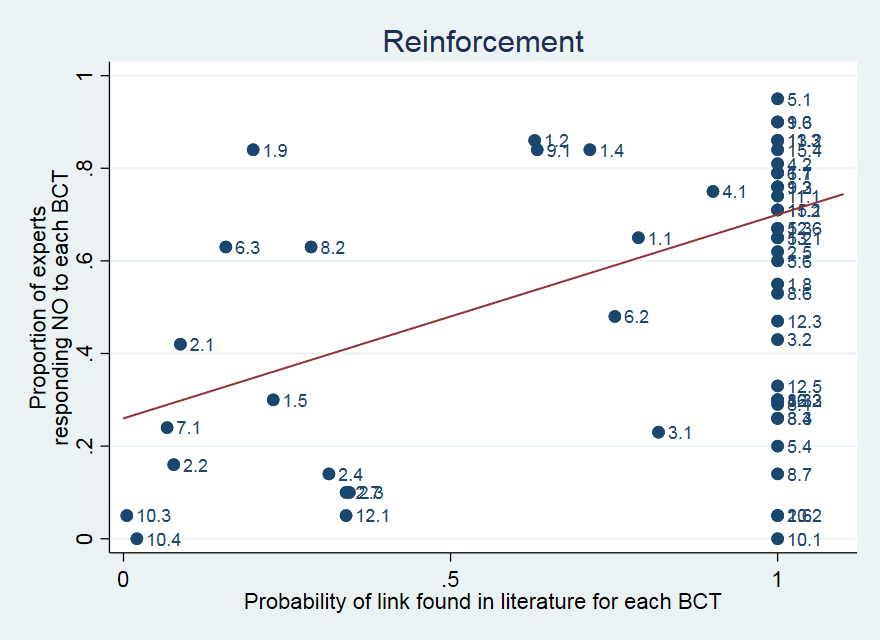


Figure 7b. Relation between literature and expert consensus when no links between BCTs and MoA “Reinforcement” have been proposed. Each dot represents one BCT (with BCTTv1 label). The line represents the prediction from the MLM omitting BCTs for which p=1 in the literature study (but the expert consensus values for such BCTs are shown).


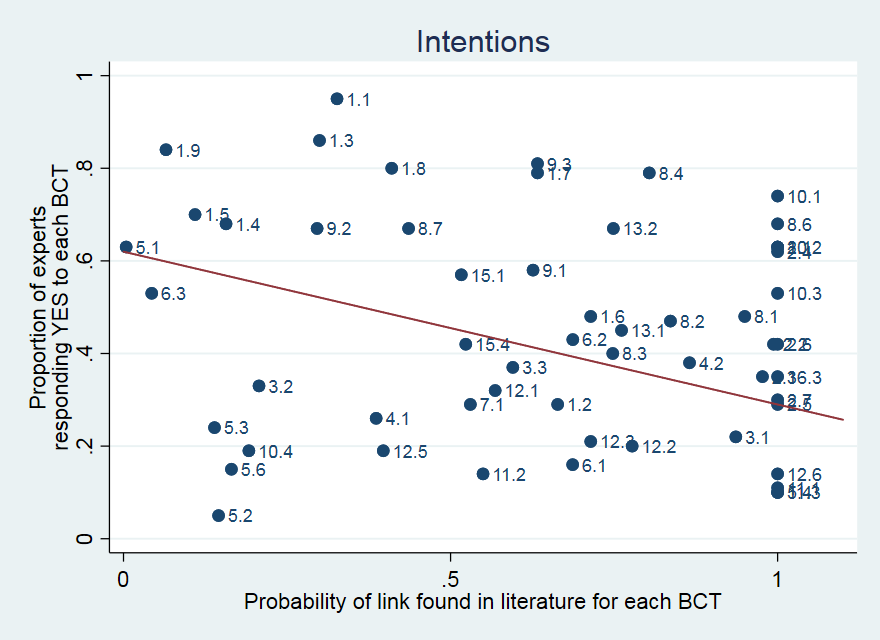


Figure 8a. Relation between literature and expert consensus when links between BCTs and MoA “Intentions” have been proposed. Each dot represents one BCT (with BCTTv1 label). The line represents the prediction from the MLM omitting BCTs for which p=1 in the literature study (but the expert consensus values for such BCTs are shown).


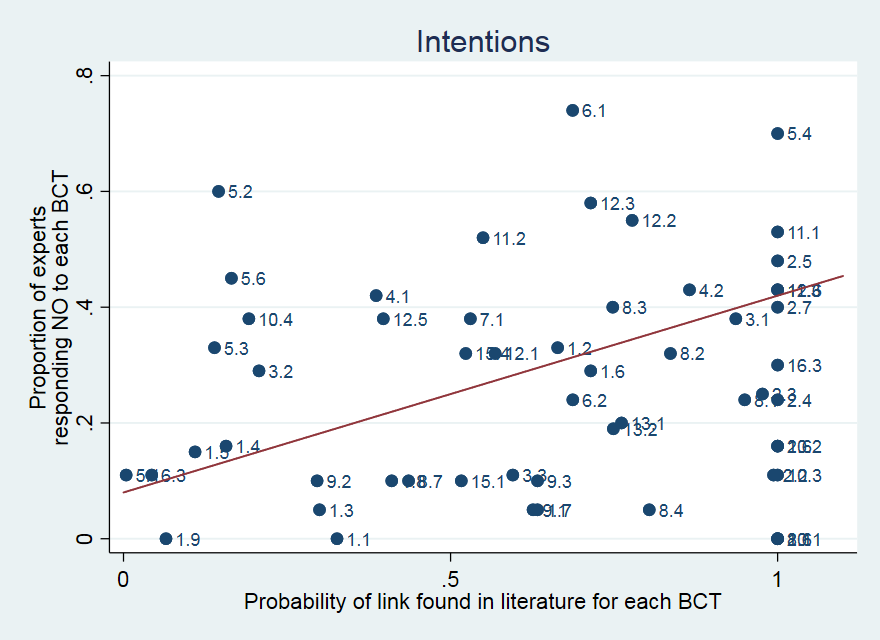


Figure 8b. Relation between literature and expert consensus when no links between BCTs and MoA “Intentions” have been proposed. Each dot represents one BCT (with BCTTv1 label). The line represents the prediction from the MLM omitting BCTs for which p=1 in the literature study (but the expert consensus values for such BCTs are shown).


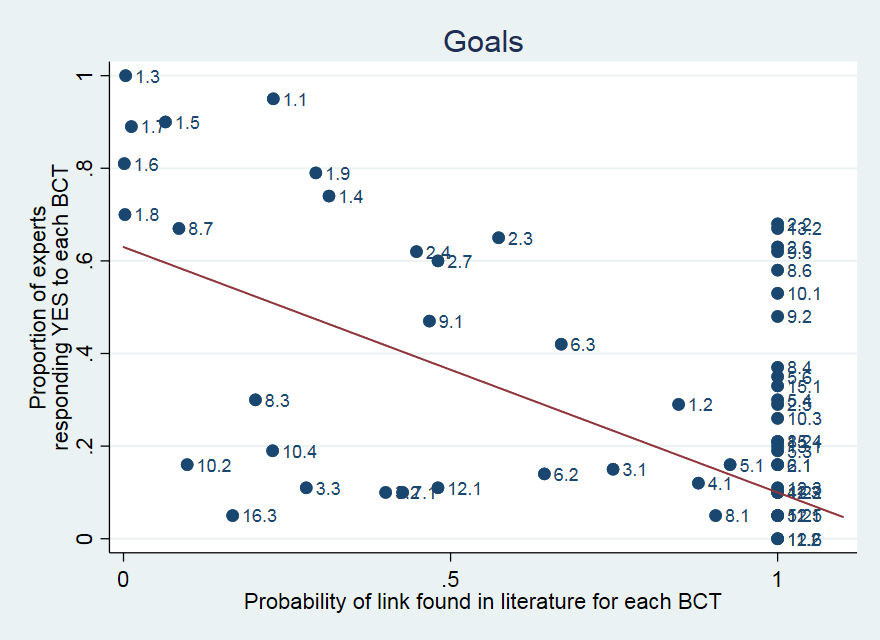


Figure 9a. Relation between literature and expert consensus when links between BCTs and MoA “Goals” have been proposed. Each dot represents one BCT (with BCTTv1 label). The line represents the prediction from the MLM omitting BCTs for which p=1 in the literature study (but the expert consensus values for such BCTs are shown).


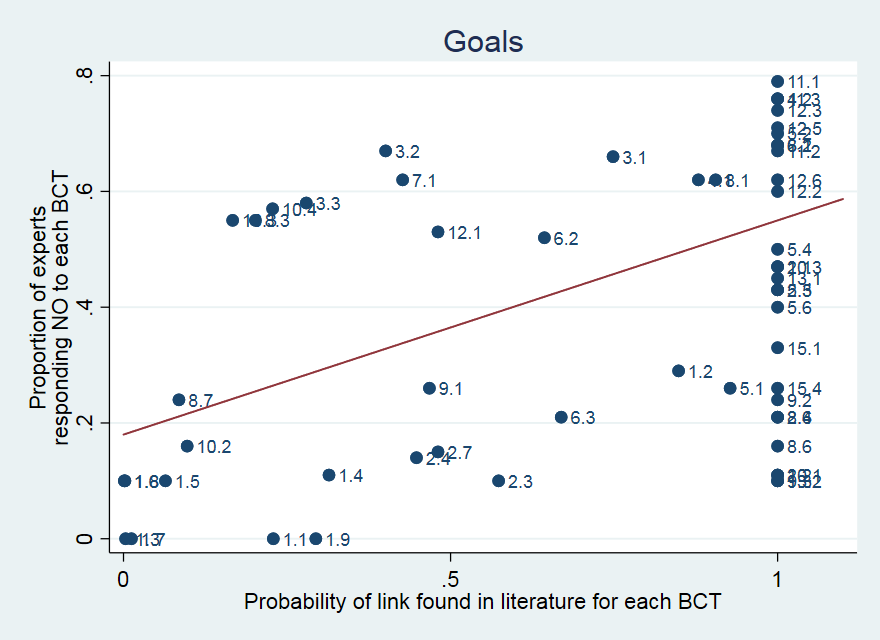


Figure 9b Relation between literature and expert consensus when no links between BCTs and MoA “Goals” have been proposed. Each dot represents one BCT (with BCTTv1 label). The line represents the prediction from the MLM omitting BCTs for which p=1 in the literature study (but the expert consensus values for such BCTs are shown).


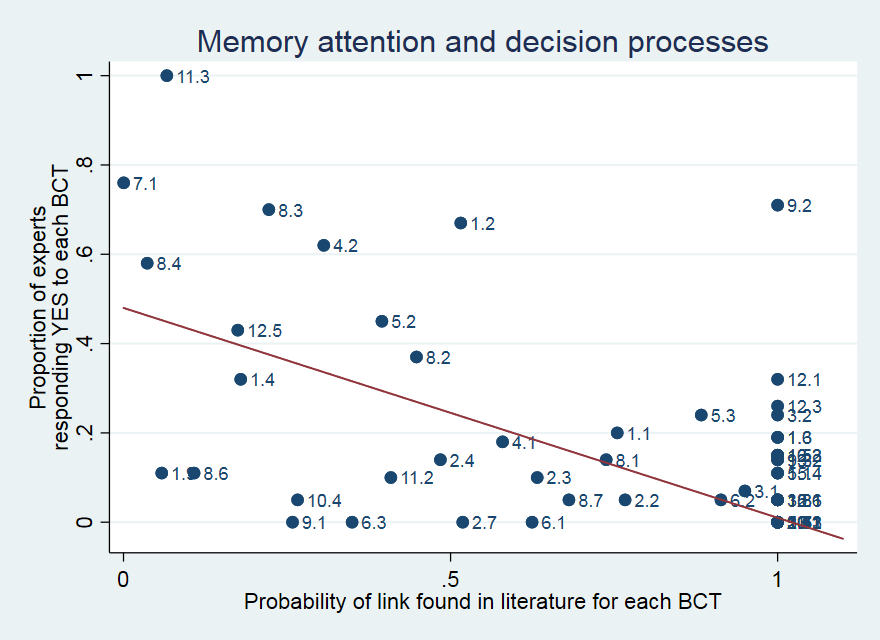


Figure 10a. Relation between literature and expert consensus when links between BCTs and MoA “Memory, attention and decision processes” have been proposed. Each dot represents one BCT (with BCTTv1 label). The line represents the prediction from the MLM omitting BCTs for which p=1 in the literature study (but the expert consensus values for such BCTs are shown).


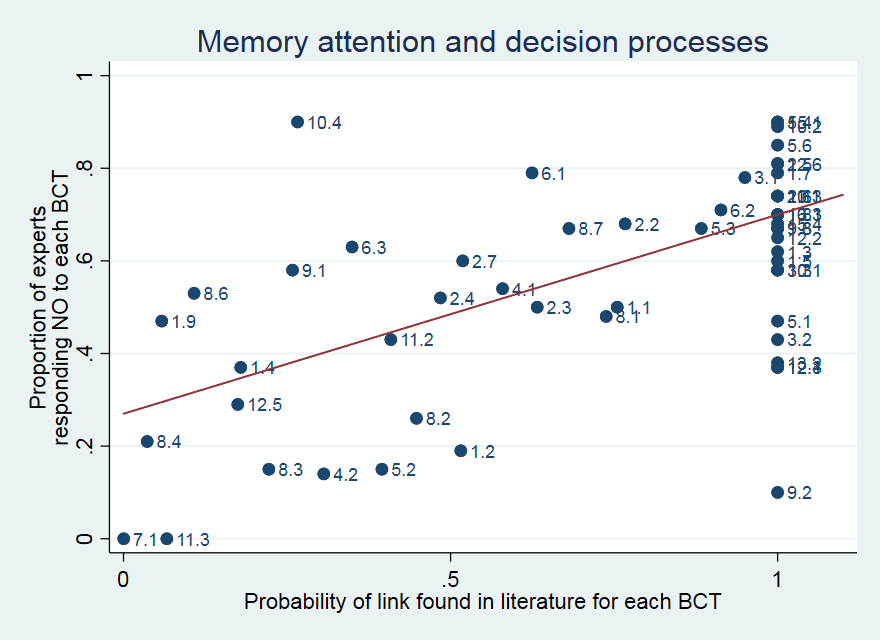


Figure 10b. Relation between literature and expert consensus when no links between BCTs and MoA “Memory, attention and decision processes” have been proposed. Each dot represents one BCT (with BCTTv1 label). The line represents the prediction from the MLM omitting BCTs for which p=1 in the literature study (but the expert consensus values for such BCTs are shown).


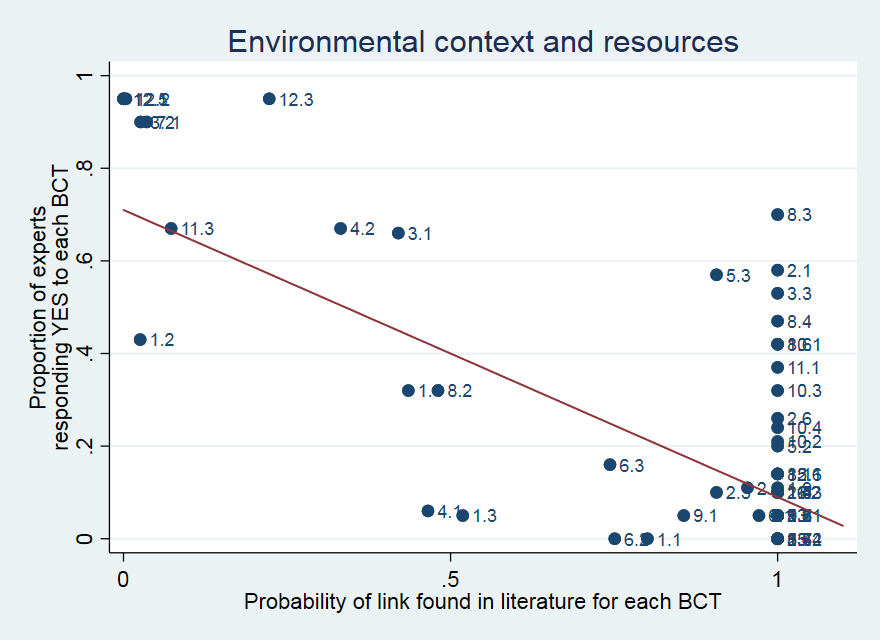


Figure 11a. Relation between literature and expert consensus when links between BCTs and MoA “Environmental context and resources” have been proposed. Each dot represents one BCT (with BCTTv1 label). The line represents the prediction from the MLM omitting BCTs for which p=1 in the literature study (but the expert consensus values for such BCTs are shown).


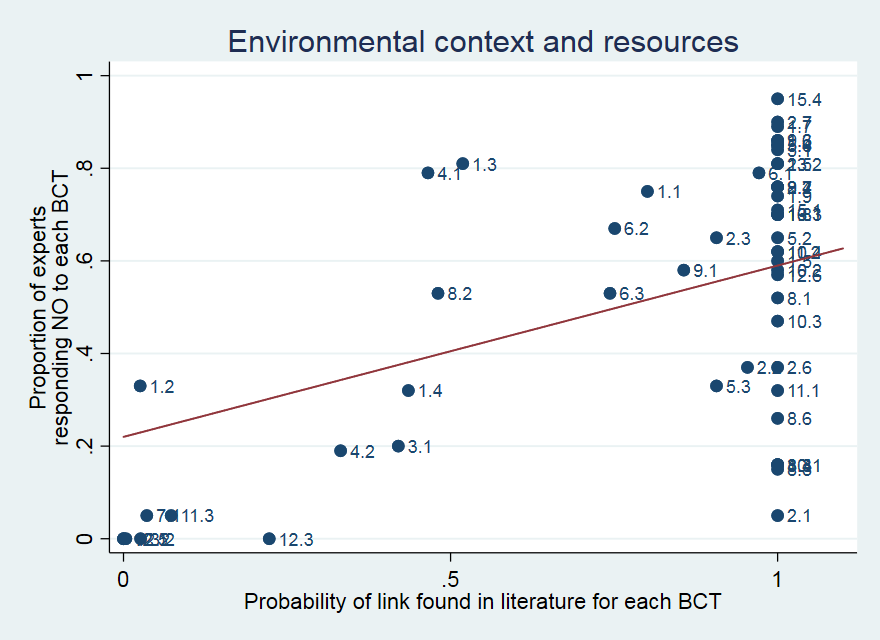


Figure 11b. Relation between literature and expert consensus when no links between BCTs and MoA “Environmental context and resources” have been proposed. Each dot represents one BCT (with BCTTv1 label). The line represents the prediction from the MLM omitting BCTs for which p=1 in the literature study (but the expert consensus values for such BCTs are shown).


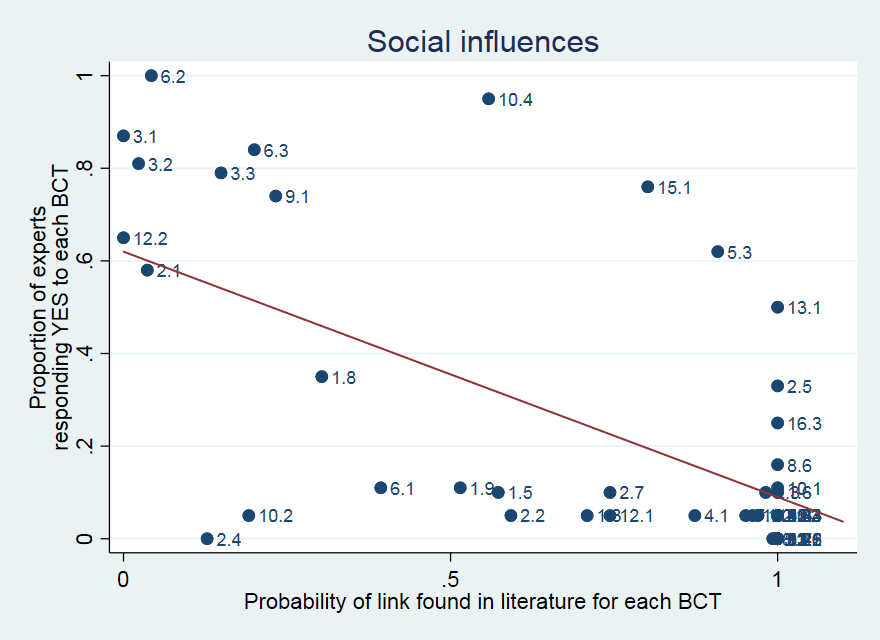


Figure 12a. Relation between literature and expert consensus when links between BCTs and MoA “Social influence” have been proposed. Each dot represents one BCT (with BCTTv1 label). The line represents the prediction from the MLM omitting BCTs for which p=1 in the literature study (but the expert consensus values for such BCTs are shown).


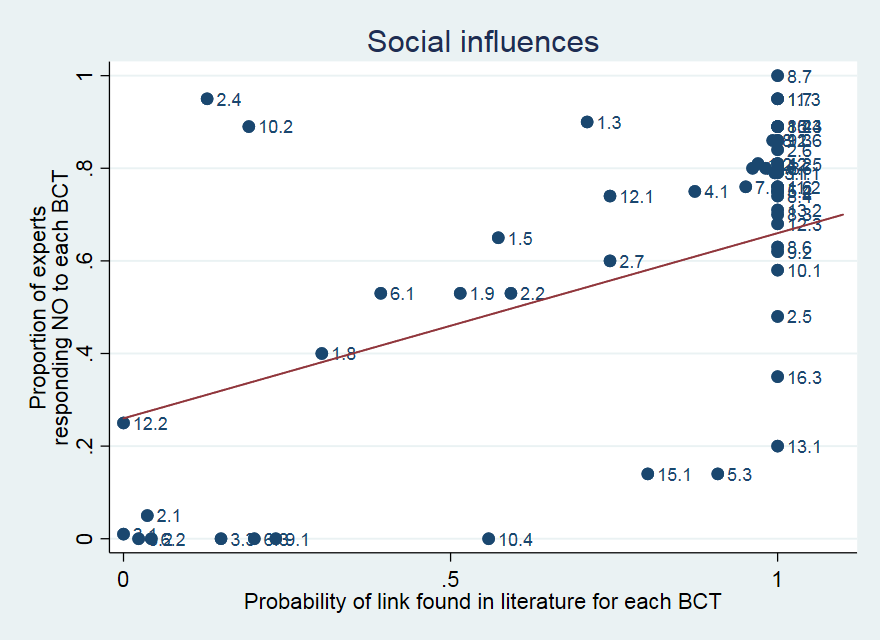


Figure 12b. Relation between literature and expert consensus when no links between BCTs and MoA “Social influence” have been proposed. Each dot represents one BCT (with BCTTv1 label). The line represents the prediction from the MLM omitting BCTs for which p=1 in the literature study (but the expert consensus values for such BCTs are shown).


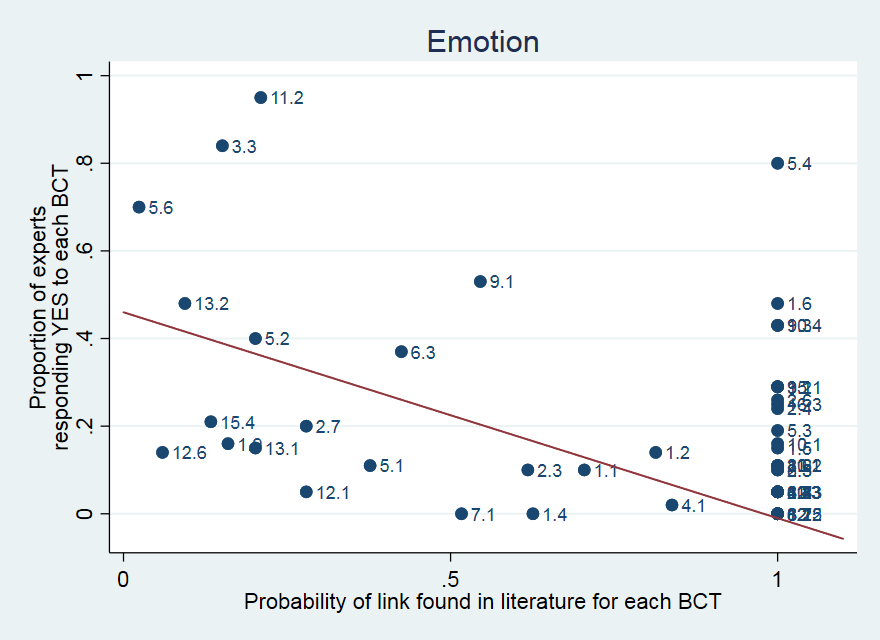


Figure 13a. Relation between literature and expert consensus when links between BCTs and MoA “Emotion” have been proposed. Each dot represents one BCT (with BCTTv1 label). The line represents the prediction from the MLM omitting BCTs for which p=1 in the literature study (but the expert consensus values for such BCTs are shown).


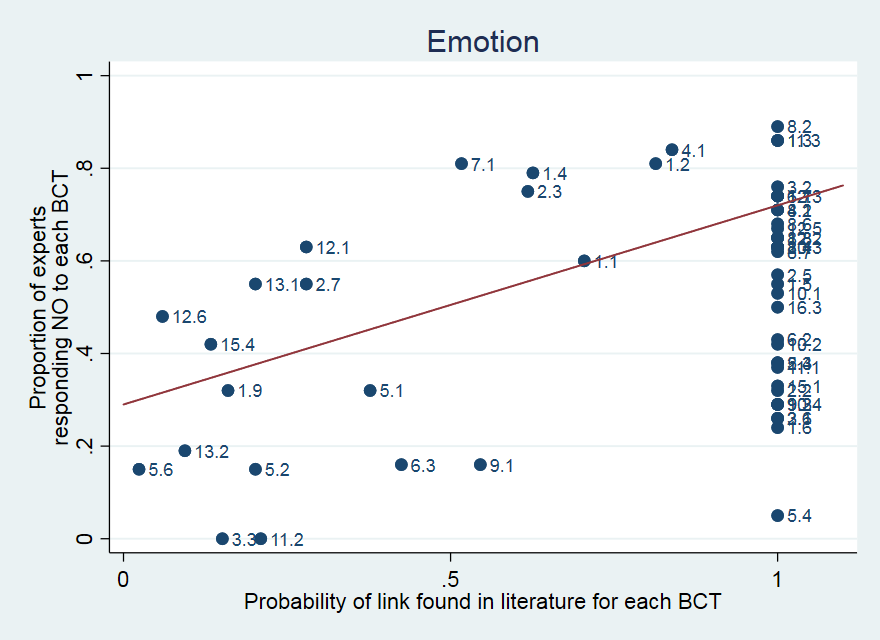


Figure 13b. Relation between literature and expert consensus when no links between BCTs and MoA “Emotion” have been proposed. Each dot represents one BCT (with BCTTv1 label). The line represents the prediction from the MLM omitting BCTs for which p=1 in the literature study (but the expert consensus values for such BCTs are shown).


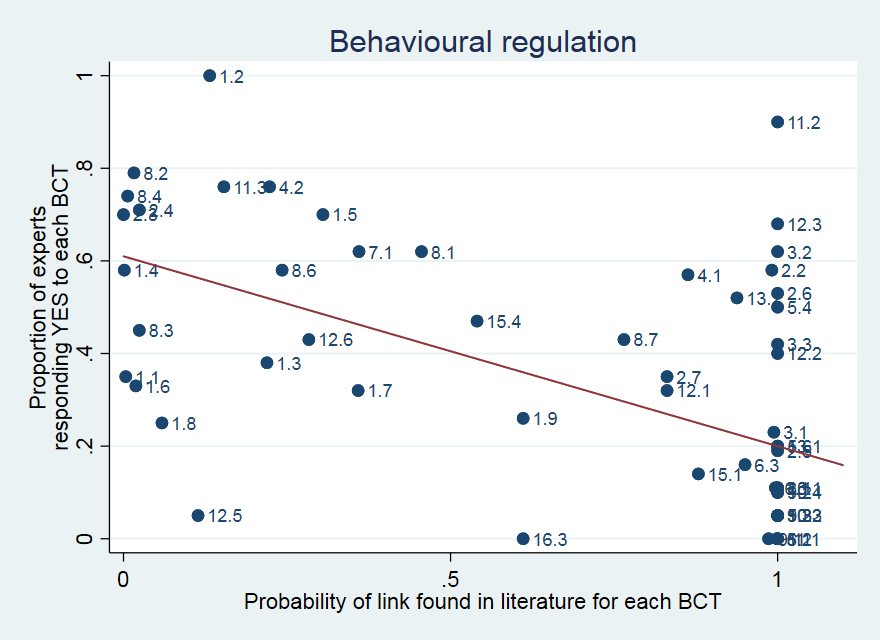


Figure 14a. Relation between literature and expert consensus when links between BCTs and MoA “Behavioural regulation” have been proposed. Each dot represents one BCT (with BCTTv1 label). The line represents the prediction from the MLM omitting BCTs for which p=1 in the literature study (but the expert consensus values for such BCTs are shown).


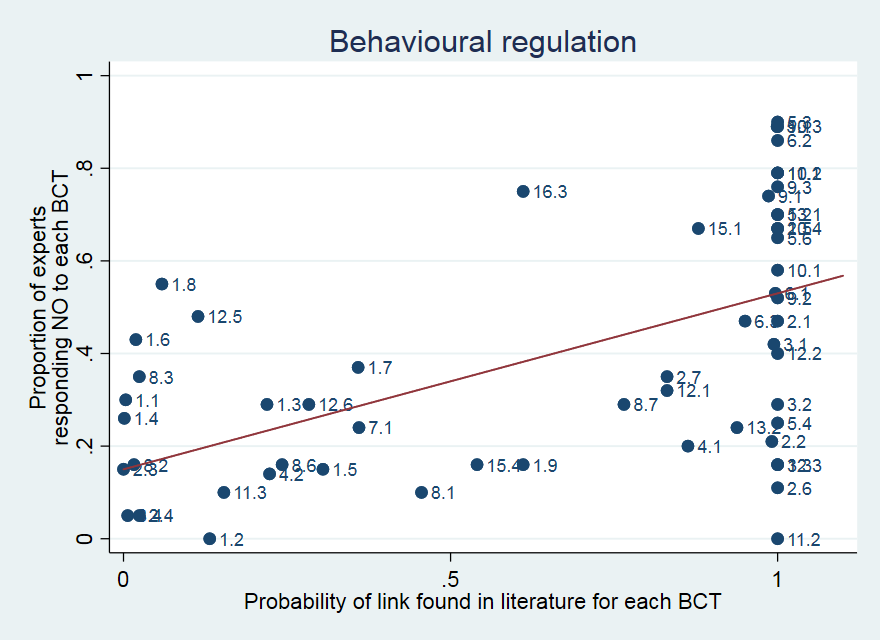


Figure 14b. Relation between literature and expert consensus when no links between BCTs and MoA “Behavioural regulation” have been proposed. Each dot represents one BCT (with BCTTv1 label). The line represents the prediction from the MLM omitting BCTs for which p=1 in the literature study (but the expert consensus values for such BCTs are shown).


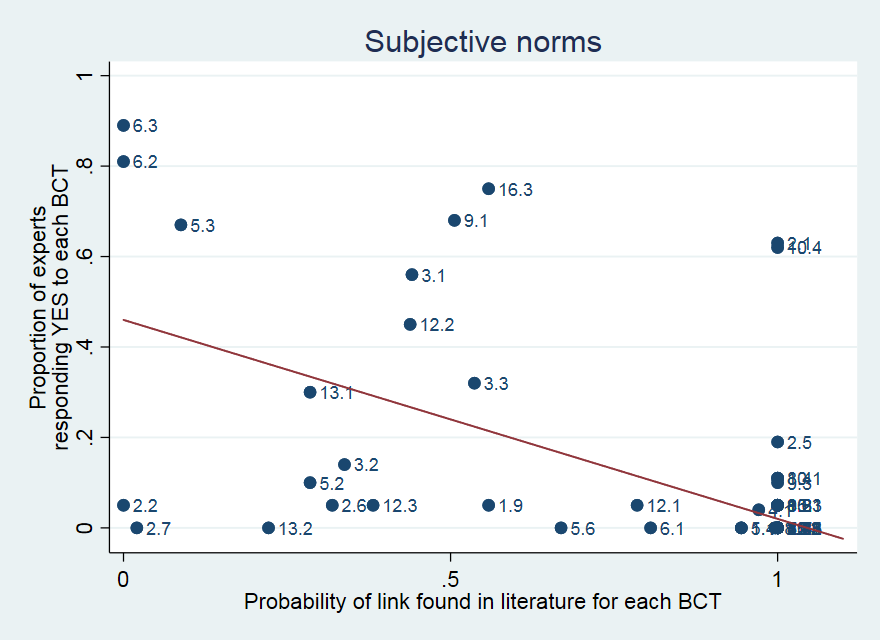


Figure 15a. Relation between literature and expert consensus when links between BCTs and MoA “Subjective knowledge” have been proposed. Each dot represents one BCT (with BCTTv1 label). The line represents the prediction from the MLM omitting BCTs for which p=1 in the literature study (but the expert consensus values for such BCTs are shown).


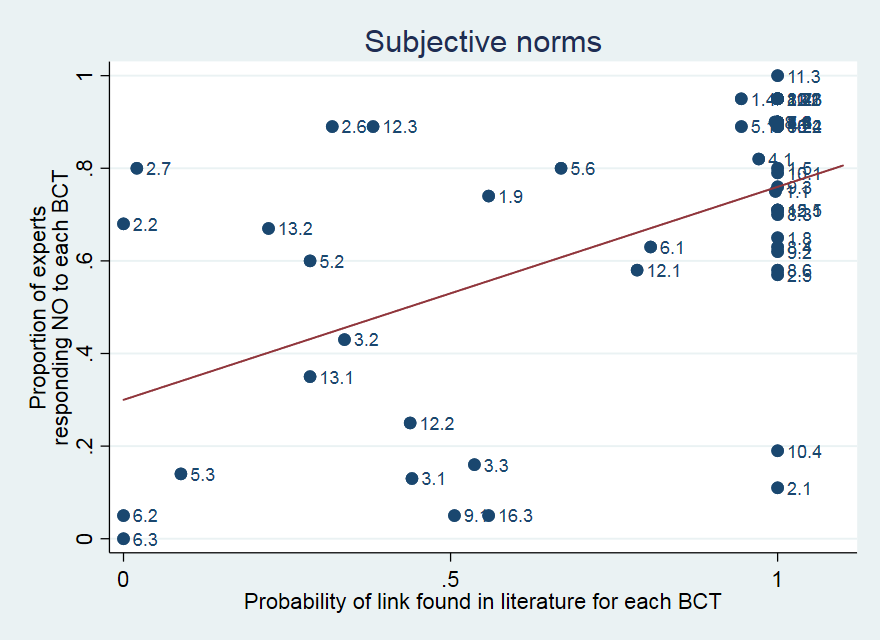


Figure 15b. Relation between literature and expert consensus when no links between BCTs and MoA “Subjective knowledge” have been proposed. Each dot represents one BCT (with BCTTv1 label). The line represents the prediction from the MLM omitting BCTs for which p=1 in the literature study (but the expert consensus values for such BCTs are shown).


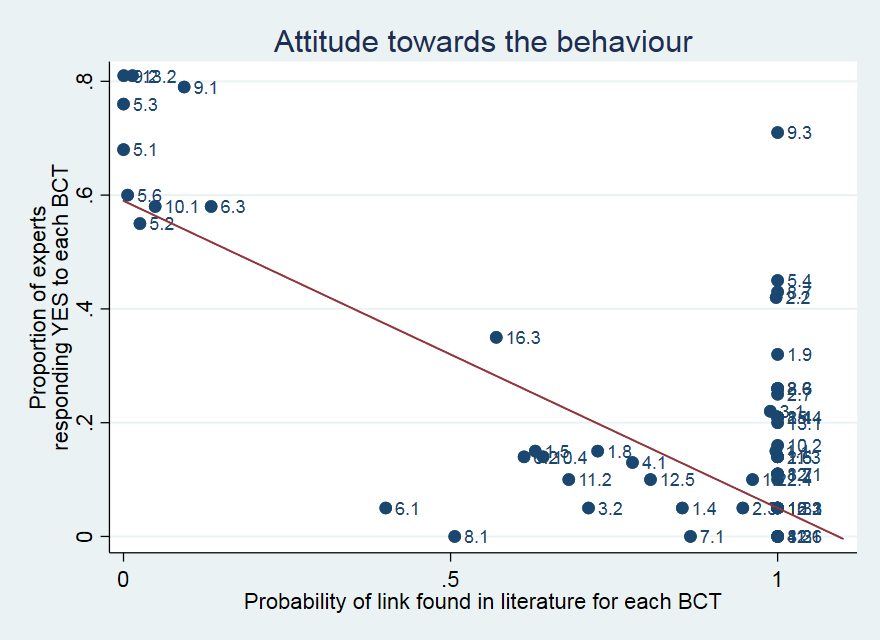


Figure 16a. Relation between literature and expert consensus when links between BCTs and MoA “Attitude towards the behaviour” have been proposed. Each dot represents one BCT (with BCTTv1 label). The line represents the prediction from the MLM omitting BCTs for which p=1 in the literature study (but the expert consensus values for such BCTs are shown).


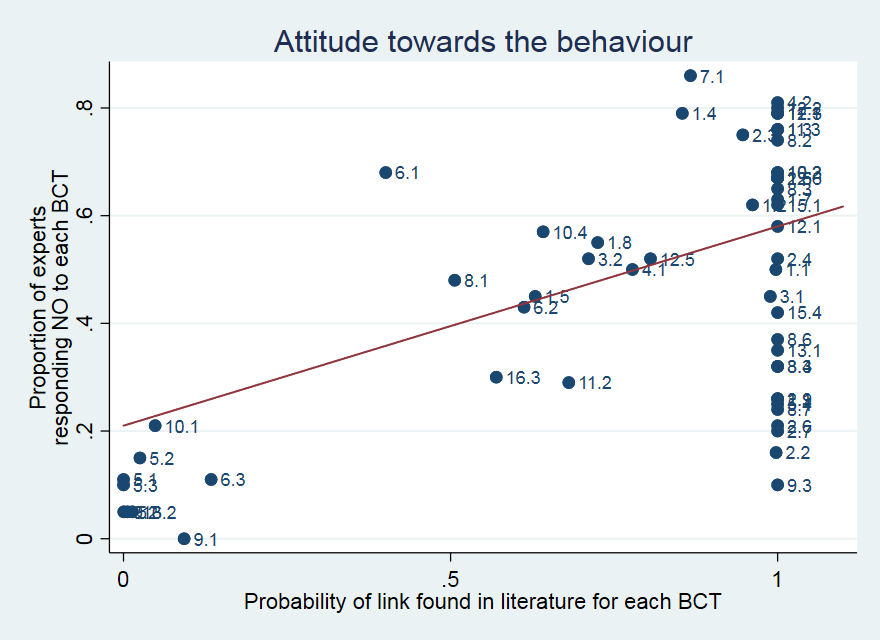


Figure 16b. Relation between literature and expert consensus when no links between BCTs and MoA “Attitude towards the behaviour” have been proposed. Each dot represents one BCT (with BCTTv1 label). The line represents the prediction from the MLM omitting BCTs for which p=1 in the literature study (but the expert consensus values for such BCTs are shown).


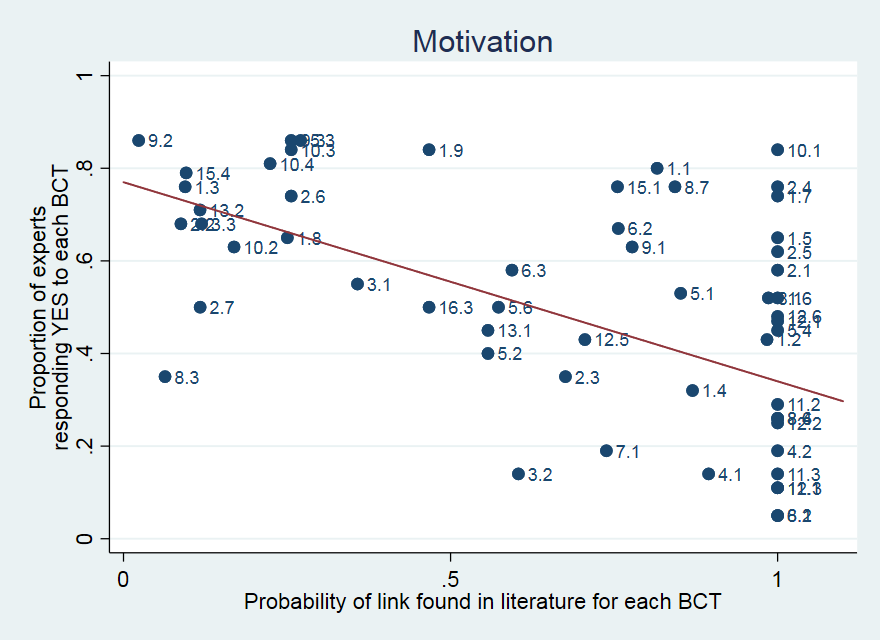


Figure 17a. Relation between literature and expert consensus when links between BCTs and MoA “Motivation” have been proposed. Each dot represents one BCT (with BCTTv1 label). The line represents the prediction from the MLM omitting BCTs for which p=1 in the literature study (but the expert consensus values for such BCTs are shown).


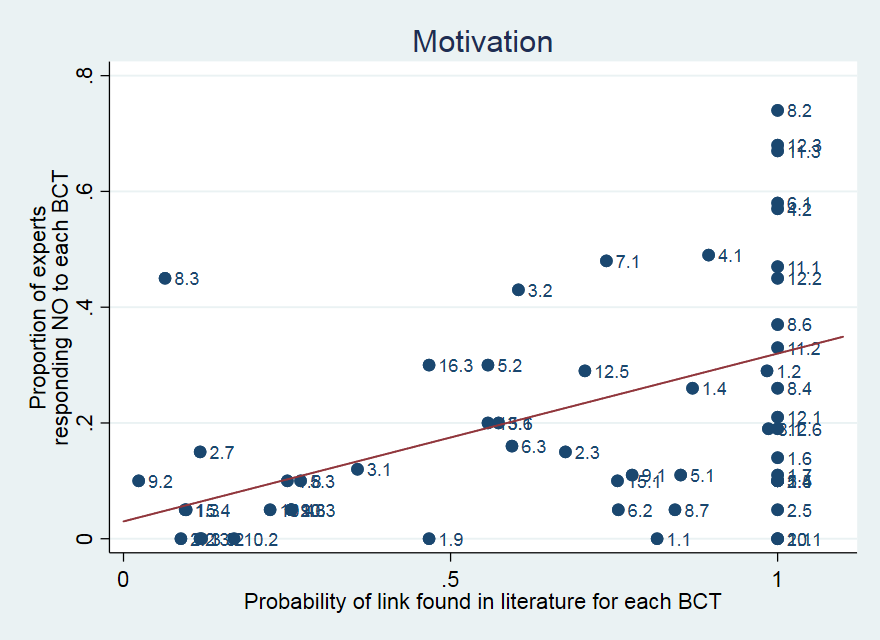


Figure 17b. Relation between literature and expert consensus when no links between BCTs and MoA “Motivation” have been proposed. Each dot represents one BCT (with BCTTv1 label). The line represents the prediction from the MLM omitting BCTs for which p=1 in the literature study (but the expert consensus values for such BCTs are shown).


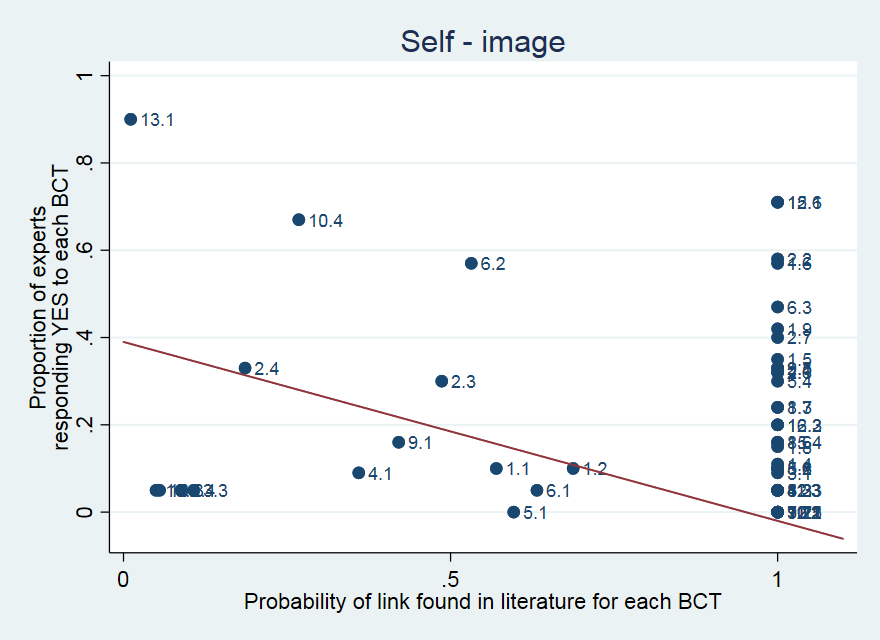


Figure 18a. Relation between literature and expert consensus when links between BCTs and MoA “Self-image” have been proposed. Each dot represents one BCT (with BCTTv1 label). The line represents the prediction from the MLM omitting BCTs for which p=1 in the literature study (but the expert consensus values for such BCTs are shown).


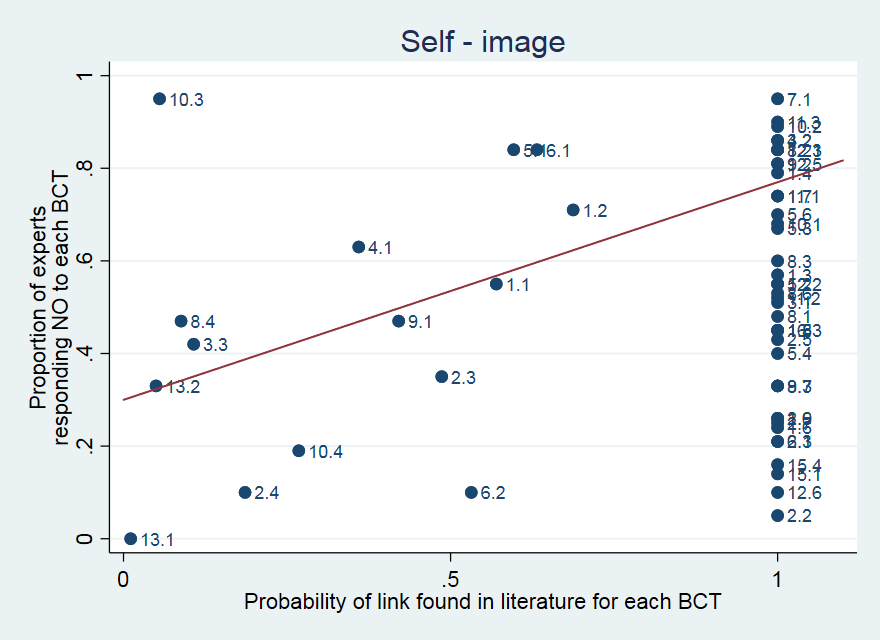


Figure 18b. Relation between literature and expert consensus when no links between BCTs and MoA “Self-image” have been proposed. Each dot represents one BCT (with BCTTv1 label). The line represents the prediction from the MLM omitting BCTs for which p=1 in the literature study (but the expert consensus values for such BCTs are shown


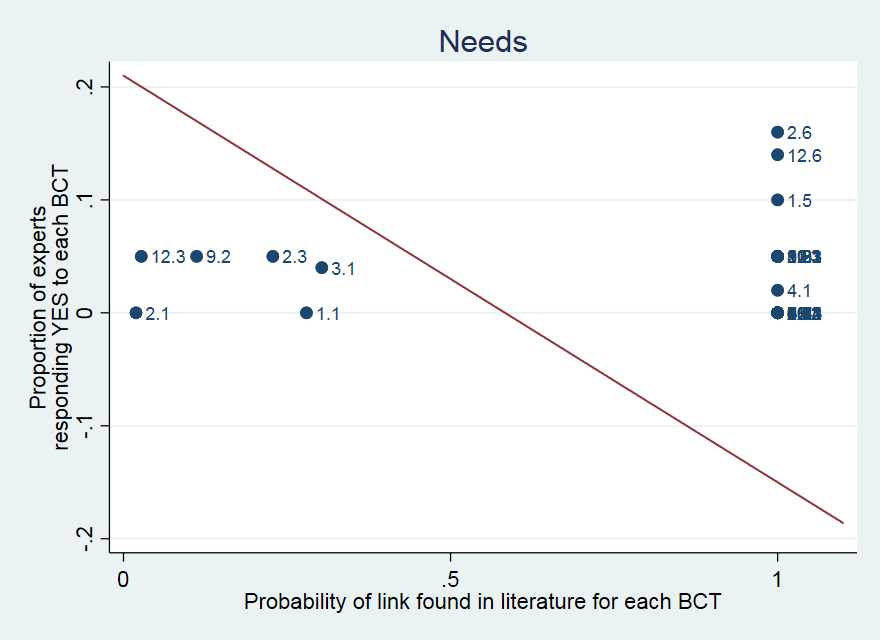


Figure 19a. Relation between literature and expert consensus when links between BCTs and MoA “Needs” have been proposed. Each dot represents one BCT (with BCTTv1 label). The line represents the prediction from the MLM omitting BCTs for which p=1 in the literature study (but the expert consensus values for such BCTs are shown).


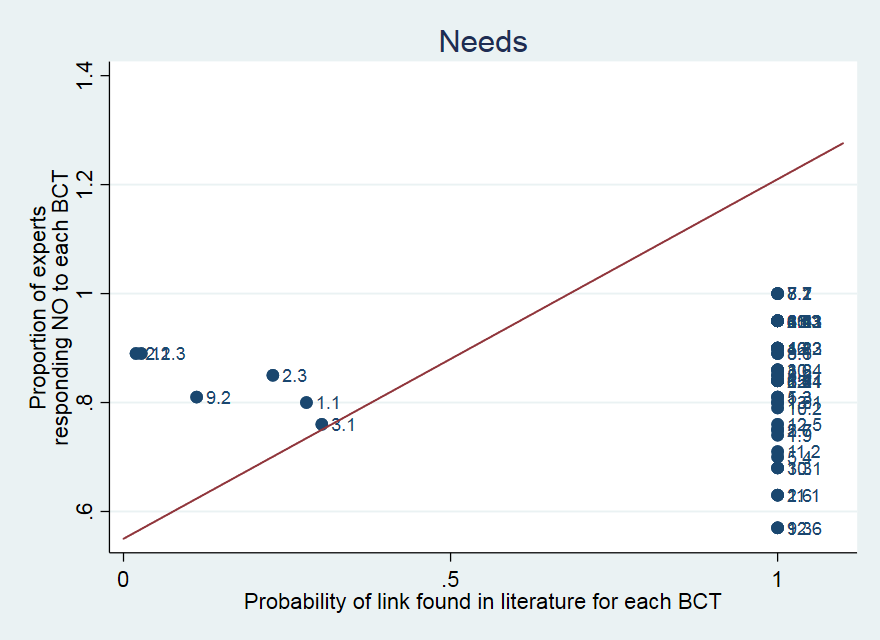


Figure 19b. Relation between literature and expert consensus when no links between BCTs and MoA “Needs” have been proposed. Each dot represents one BCT (with BCTTv1 label). The line represents the prediction from the MLM omitting BCTs for which p=1 in the literature study (but the expert consensus values for such BCTs are shown).


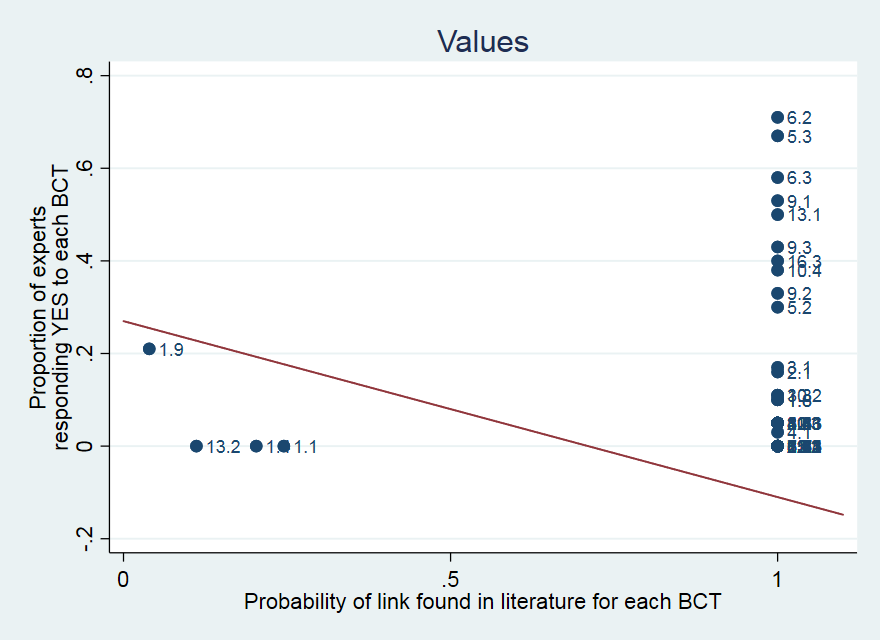


Figure 20a. Relation between literature and expert consensus when links between BCTs and MoA “Values” have been proposed. Each dot represents one BCT (with BCTTv1 label). The line represents the prediction from the MLM omitting BCTs for which p=1 in the literature study (but the expert consensus values for such BCTs are shown).


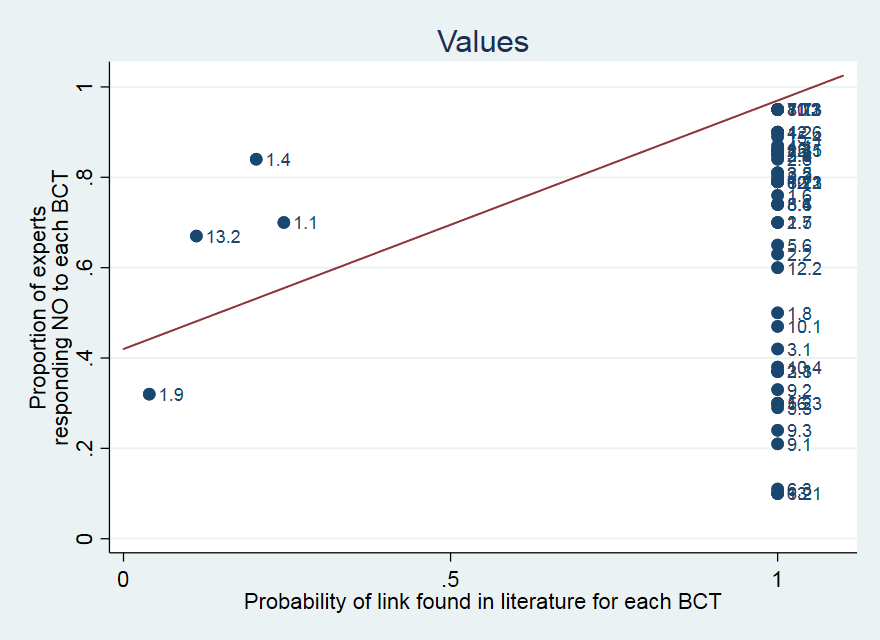


Figure 20b. Relation between literature and expert consensus when no links between BCTs and MoA “Values” have been proposed. Each dot represents one BCT (with BCTTv1 label). The line represents the prediction from the MLM omitting BCTs for which p=1 in the literature study (but the expert consensus values for such BCTs are shown).


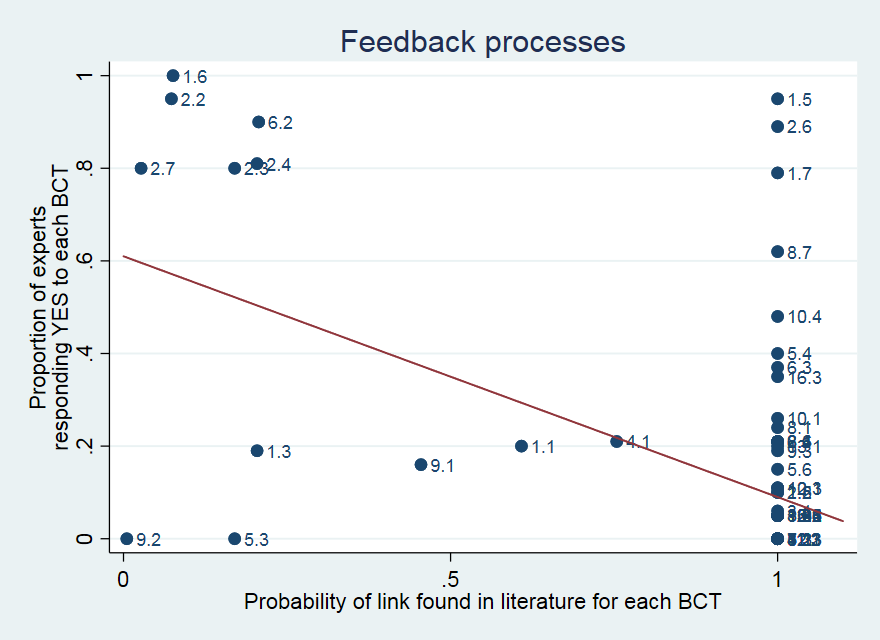


Figure 21a. Relation between literature and expert consensus when links between BCTs and MoA “Feedback processes” have been proposed. Each dot represents one BCT (with BCTTv1 label). The line represents the prediction from the MLM omitting BCTs for which p=1 in the literature study (but the expert consensus values for such BCTs are shown).


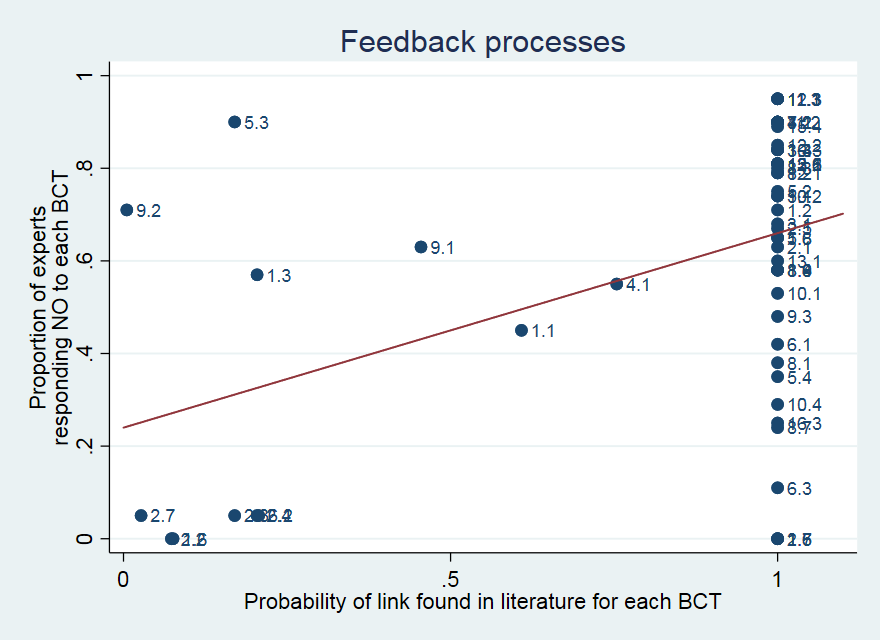


Figure 21ab. Relation between literature and expert consensus when no links between BCTs and MoA “Feedback processes” have been proposed. Each dot represents one BCT (with BCTTv1 label). The line represents the prediction from the MLM omitting BCTs for which p=1 in the literature study (but the expert consensus values for such BCTs are shown).


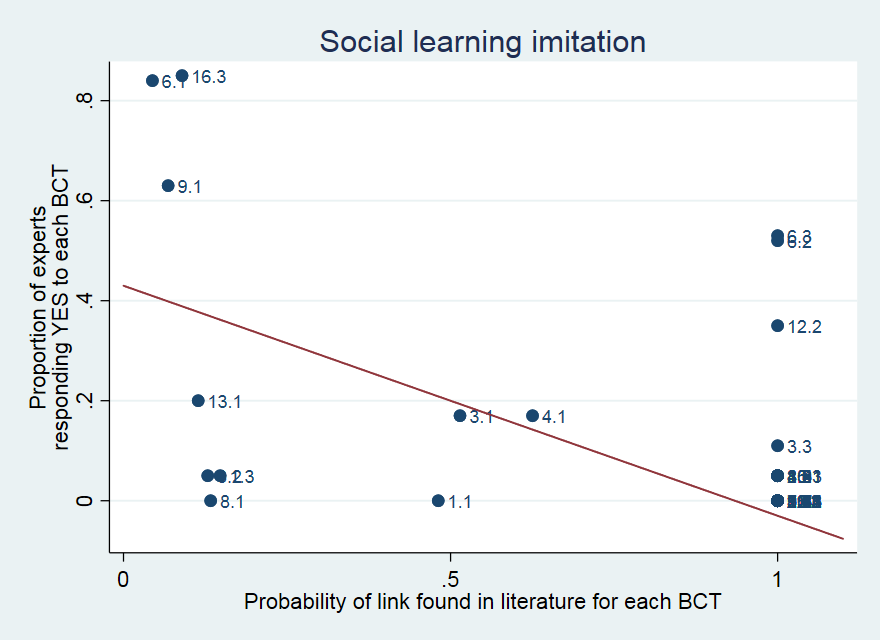


Figure 22a. Relation between literature and expert consensus when links between BCTs and MoA “Social learning/imitation” have been proposed. Each dot represents one BCT (with BCTTv1 label). The line represents the prediction from the MLM omitting BCTs for which p=1 in the literature study (but the expert consensus values for such BCTs are shown).


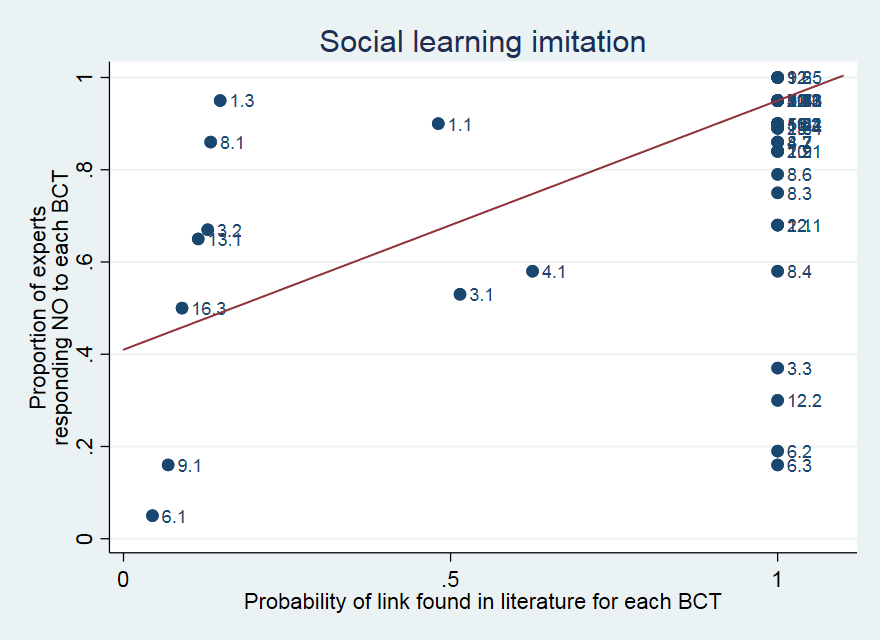


Figure 22b. Relation between literature and expert consensus when no links between BCTs and MoA “Social learning/imitation” have been proposed. Each dot represents one BCT (with BCTTv1 label). The line represents the prediction from the MLM omitting BCTs for which p=1 in the literature study (but the expert consensus values for such BCTs are shown).


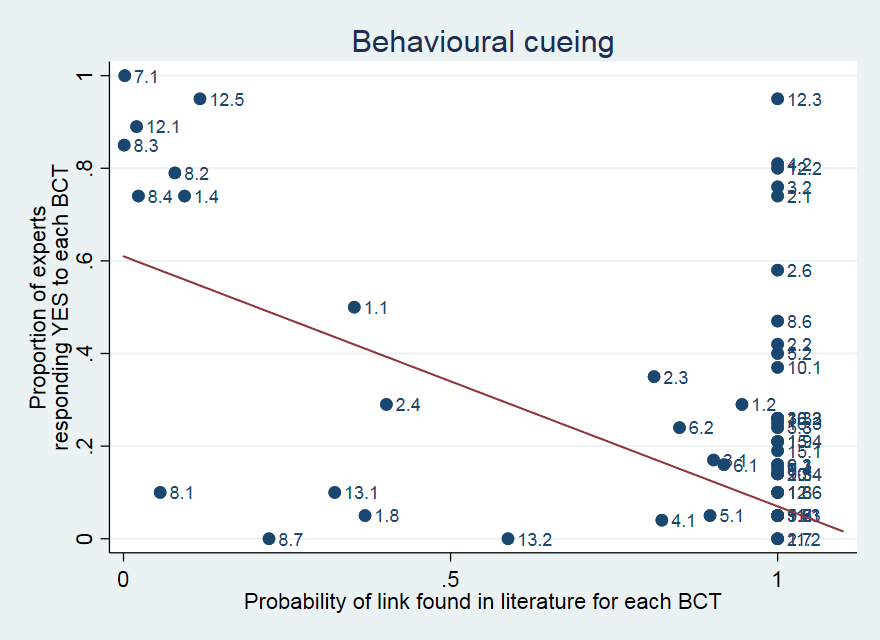


Figure 23a. Relation between literature and expert consensus when links between BCTs and MoA “Behavioral cueing” have been proposed. Each dot represents one BCT (with BCTTv1 label). The line represents the prediction from the MLM omitting BCTs for which p=1 in the literature study (but the expert consensus values for such BCTs are shown).


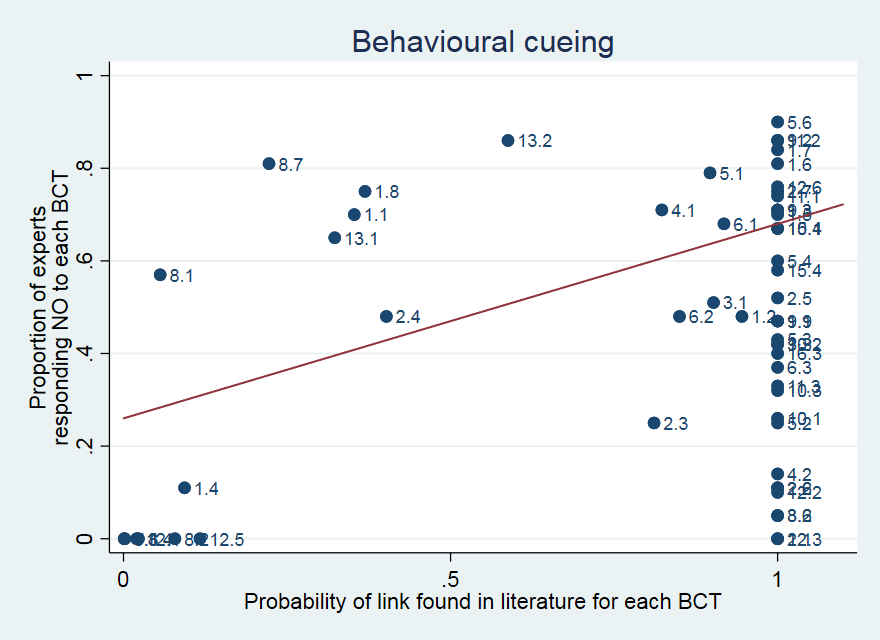


Figure 23b. Relation between literature and expert consensus when no links between BCTs and MoA “Behavioral cueing” have been proposed. Each dot represents one BCT (with BCTTv1 label). The line represents the prediction from the MLM omitting BCTs for which p=1 in the literature study (but the expert consensus values for such BCTs are shown


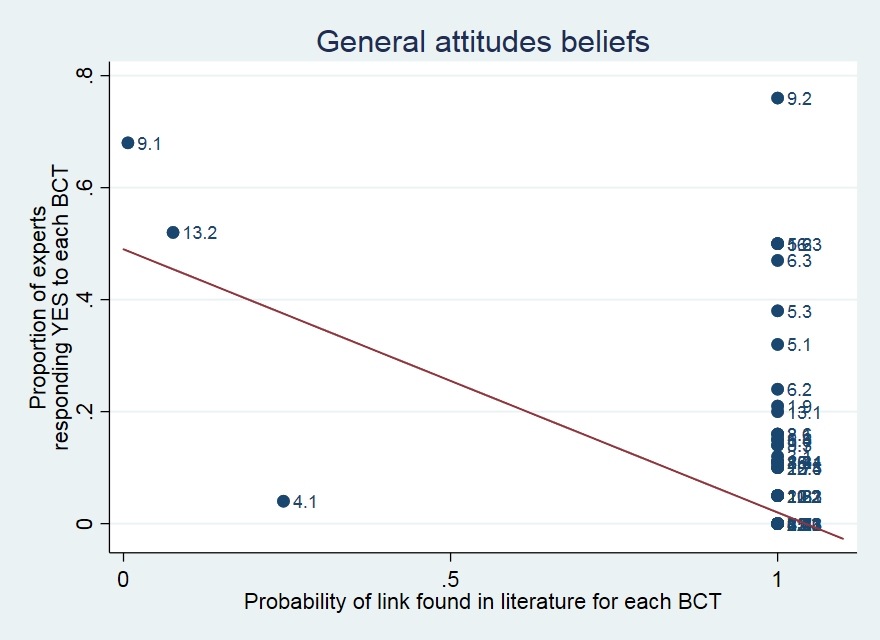


Figure 24a. Relation between literature and expert consensus when links between BCTs and MoA “General attitudes/beliefs” have been proposed. Each dot represents one BCT (with BCTTv1 label). The line represents the prediction from the MLM omitting BCTs for which p=1 in the literature study (but the expert consensus values for such BCTs are shown).


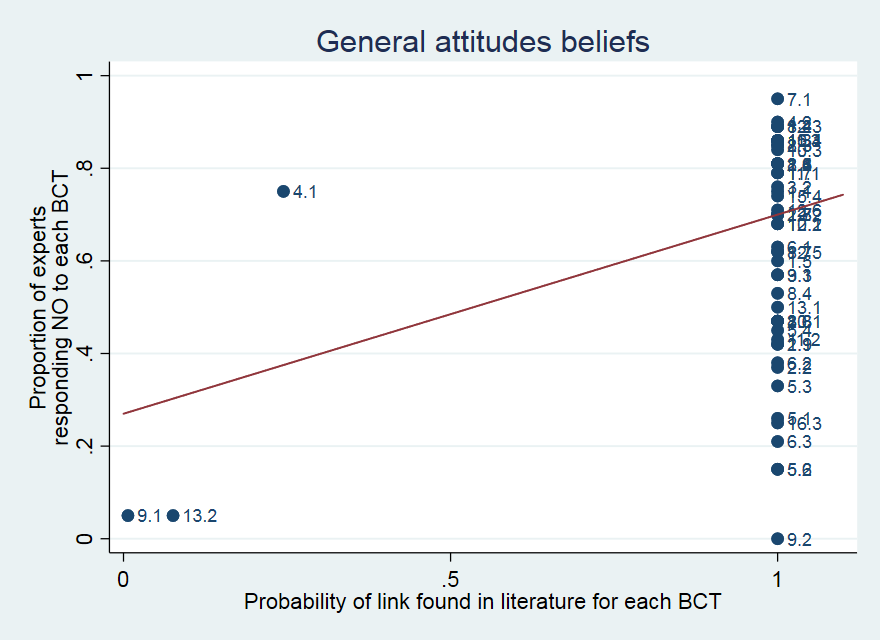


Figure 24b. Relation between literature and expert consensus when no links between BCTs and MoA “General attitudes/beliefs” have been proposed. Each dot represents one BCT (with BCTTv1 label). The line represents the prediction from the MLM omitting BCTs for which p=1 in the literature study (but the expert consensus values for such BCTs are shown).


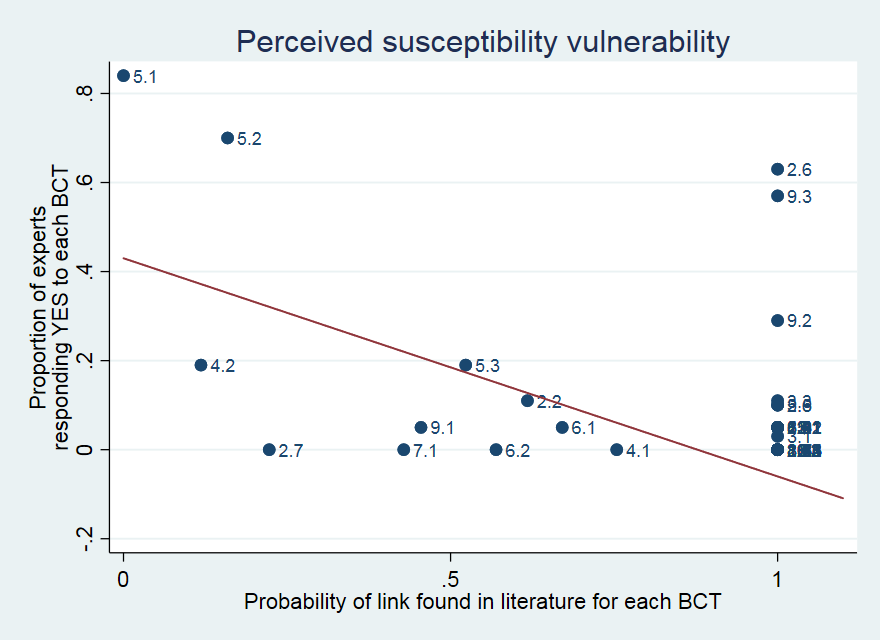


Figure 25a. Relation between literature and expert consensus when links between BCTs and MoA “Perceived susceptibility/vulnerability” have been proposed. Each dot represents one BCT (with BCTTv1 label). The line represents the prediction from the MLM omitting BCTs for which p=1 in the literature study (but the expert consensus values for such BCTs are shown).


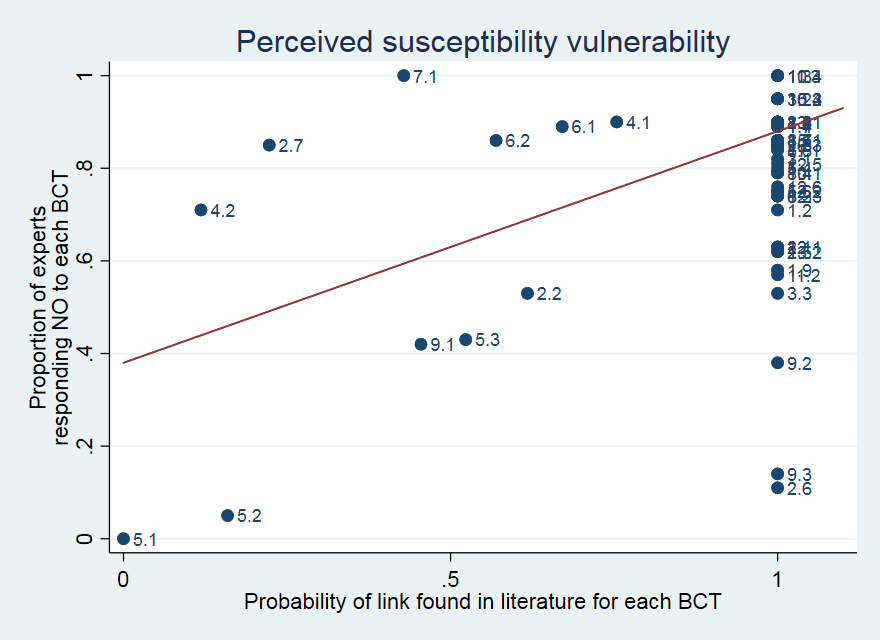


Figure 25b. Relation between literature and expert consensus when no links between BCTs and MoA “Perceived susceptibility/vulnerability” have been proposed. Each dot represents one BCT (with BCTTv1 label). The line represents the prediction from the MLM omitting BCTs for which p=1 in the literature study (but the expert consensus values for such BCTs are shown).
