# Supplementary material for: Development of an online tool for linking behavior change techniques and mechanisms of action based on triangulation of findings from literature synthesis and expert consensus
Source: Transl Behav Med. 2020 Aug 4;11(5):1049–65. doi: 10.1093/tbm/ibaa050 (PMC8158171; doi:10.1093/tbm/ibaa050)

Electronic supplementary material File 3

**The Theory and Techniques Tool (TATT):** screen shots

Figure S1 TATT illustration of results of clicking on a cell for a) a ‘link’ and b) a ‘non-link’

Figure S2 TATT illustration of screens for a) Resource and b) Collaboration

Figure S1: TATT illustration of results of clicking on a cell for a) a ‘link’ and b) a ‘non-link’

a)


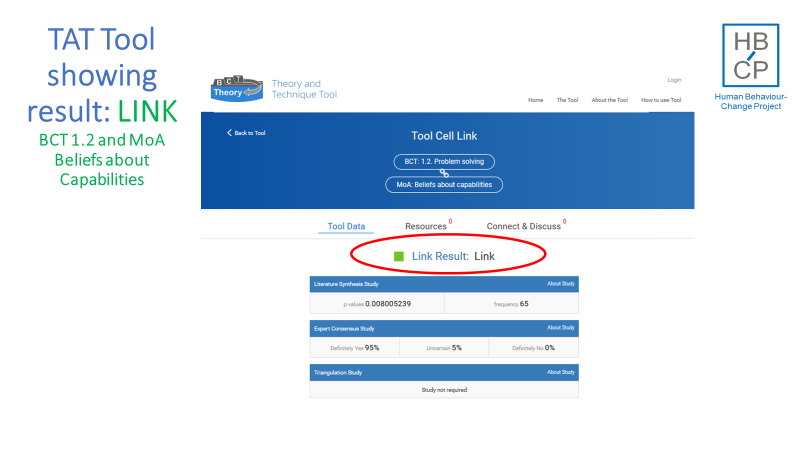


b)


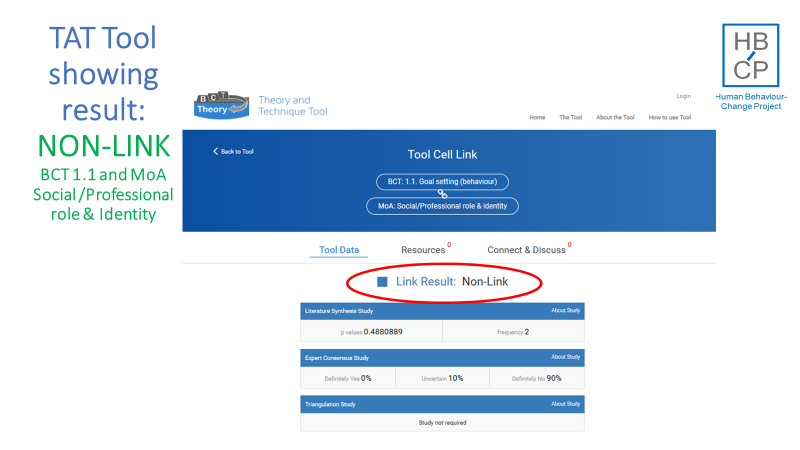


Figure S2: TATT illustration of screens for a) Resources and b) Collaboration relted to a cell of the matrix

a)
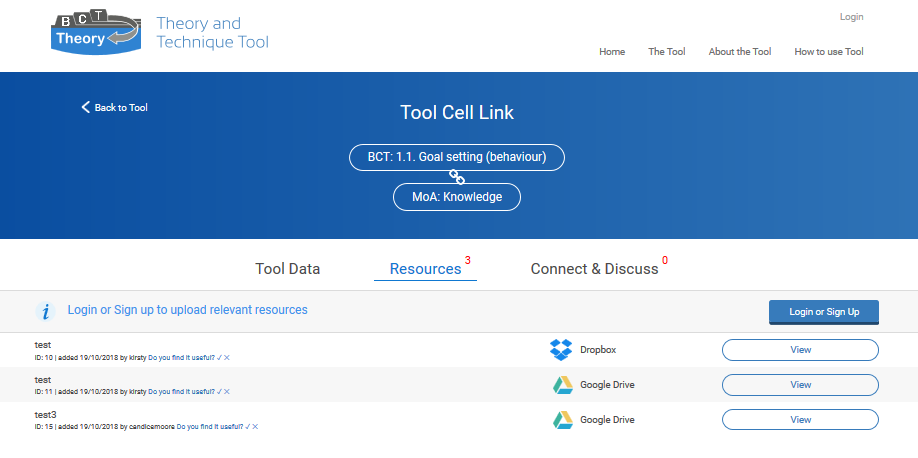


b)


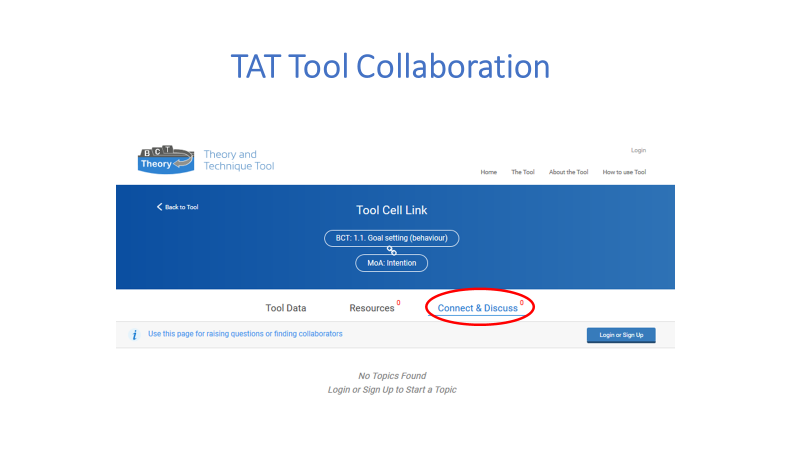

Supplement: ibaa050_suppl_Supplementary-File-3 [file ibaa050_suppl_supplementary-file-3.docx]
