# Supplementary material for: Development of an online tool for linking behavior change techniques and mechanisms of action based on triangulation of findings from literature synthesis and expert consensus
Source: Transl Behav Med. 2020 Aug 4;11(5):1049–65. doi: 10.1093/tbm/ibaa050 (PMC8158171; doi:10.1093/tbm/ibaa050)
Supplement: ibaa050_suppl_Supplementary-File-4 [file ibaa050_suppl_supplementary-file-4.pdf]

Guidelines for Round 1 of Consensus Exercise – Triangulation

Background & Aim: The Theories and Techniques of Behaviour Change Project (www.ucl.ac.uk/behaviour-change-techniques) aims to identify hypothesised links between behaviour change techniques (BCTs) and the mechanisms of action through which they influence behaviour. The first two studies in this project evaluated evidence of BCT-mechanism of action links from (i) literature synthesis and (ii) expert consensus. The next phase of the research aims to integrate findings from the two previous studies, and to identify final, agreed links between BCTs and mechanisms of action.

Participants: You are one of 17 experts from 5 countries, selected because you have a high level of expertise in theories and BCTs.

Overview of Study: The consensus exercise is a modified Nominal Group Technique (NGT) involving 3 rounds. In Round 1, you will be directed to an online questionnaire and asked to consider, in light of the data from the literature synthesis and expert consensus (which will be presented on screen), whether a specific BCT is likely to change behaviour through a particular mechanism of action (see Figure 1). In Round 2, you will be invited to participate in an online, asynchronous (i.e. each member can participate at a time of their choosing), anonymous discussion in which you will have the opportunity to exchange views with other experts. In Round 3, you will be presented with the BCTs and mechanisms of action as in Round 1 and asked to provide final ratings (which may or may not be the same as your previous ratings). At this point, you will also be asked for your own expert opinion regarding the link. More detail on Round 1 can be found below; Guidelines for Rounds 2 and 3 will be sent out separately.

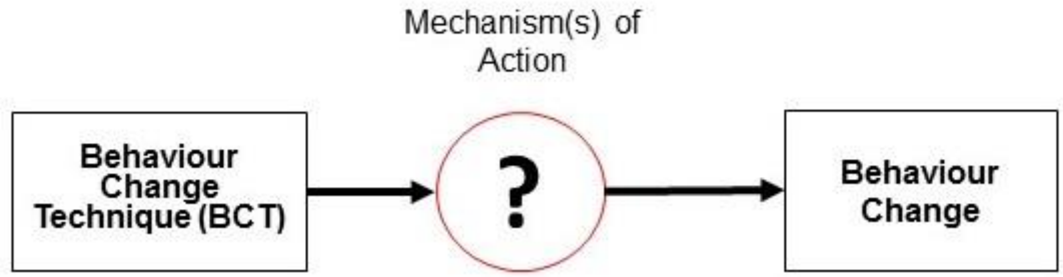

Figure 1. BCT changing behaviour through mechanism(s) of action. Note that there may be more than one mechanism of action for any BCT, and a BCT–mechanism of action link may be direct or indirect.

Theories and Techniques of Behaviour Change Project
Susan Michie, Marie Johnston, Alex Rothman, Mike Kelly, Marijn de Bruin
Email bct.theory@ucl.ac.uk

Round 1 Guidelines

Information Provided

In Appendix D, you will find confidential data relating to inconsistencies and uncertainties between the literature synthesis and expert consensus findings. This information will be presented to you on screen during the consensus exercise, and relates to the following types of hypothesised BCT-mechanism of action links:

a) Evidence indicates a link in the literature synthesis, evidence indicates 'definitely no' link in expert consensus
b) Evidence indicates a link in the literature synthesis, disagreement regarding the link in expert consensus
c) No evidence to indicate a link in the literature synthesis, evidence indicates 'definitely' a link in expert consensus
d) Marginal evidence to indicate a link in the literature synthesis and/or expert consensus.

Question

Links from the four categories above will be presented in a randomised order, along with data from the literature synthesis and expert consensus. You will be asked to answer the following question:

1. **<u>Based on the data presented</u>, when [BCT X] changes behaviour, is it likely to do so by changing [Mechanism of Action Y]?**

Response Options

a) 'Definitely Yes': Based on the data provided from the literature synthesis and expert consensus, when this BCT is effective in changing behaviour, it does so by changing this mechanism of action.
b) 'Definitely No': Based on the data provided from the literature synthesis and expert consensus, when this BCT is effective in changing behaviour, it does not do so by changing this mechanism of action.
c) 'Uncertain / Don't Know': Based on the data provided from the literature synthesis and expert consensus, you are uncertain about whether or not the BCT changes behaviour by changing this mechanism of action.

Your response to all questions in this first round should be based <u>only</u> on the data from the literature synthesis and expert consensus.

When providing your ratings, please remember:

Definitions for all BCTs and mechanisms of action will appear on screen during the consensus exercise (see Appendices A & B). Please read and re-read these definitions before beginning the task. Definitions can also be accessed via www.bcts.23.co.uk, and through the BCTTv1 smartphone app, which can be downloaded for free via the Apple App and Google Play stores.

Appendix A: Behaviour Change Techniques

Behaviour Change Techniques (BCTs) are the potentially active ingredients of behaviour change interventions. For this study, BCTs will be taken from the 93-item BCT Taxonomy Version 1 (BCTTv1) (Michie et al., 2013). A user-friendly version of BCTTv1, including definitions and examples, can be found via the app (details above), or www.bcts.23.co.uk:

When you enter the website you will see this page, and view BCTs according to their hierarchical categories. You can return to this view at any time by clicking 'Grouped'. The categories can be clicked to view the BCTs contained within that category.

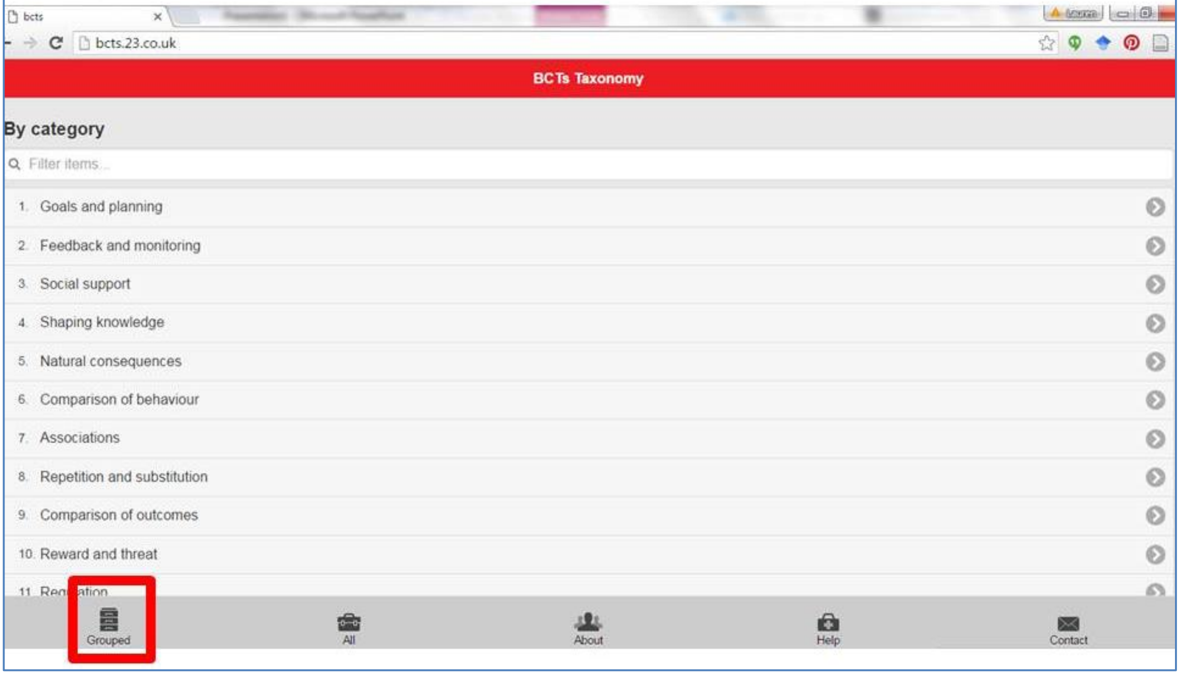

If you would like to view/search all BCTs individually, you can click the 'All' button at the bottom of the page (indicated in red below). In 'All BCTs' you can search for a particular BCT in the search bar at the top of the page (indicated in blue below).

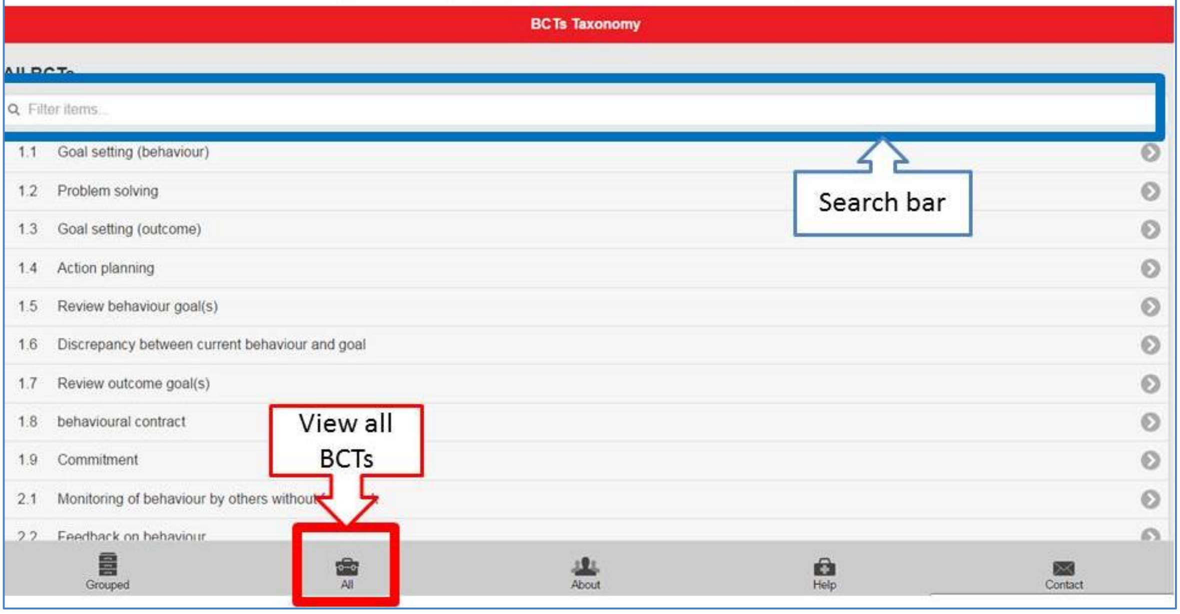

Appendix B: Mechanisms of Action

Mechanisms of action are defined as "the processes through which a BCT affects behaviour". Below are 26 mechanisms of action, taken from the theoretical domains as described in the Theoretical Domains Framework (Cane et al., 2012) and the most frequently occurring mechanisms derived from a set of 83 behaviour change theories (Michie, West, et al., 2014). You will be asked about a number of these during the consensus exercise:

	**Mechanism Definition**	**Mechanism Label**
1.	An awareness of the existence of something	Knowledge
2.	An ability or proficiency acquired through practice	Skills
3.	A coherent set of behaviours and displayed personal qualities of an individual in a social or work setting	Social/ Professional Role and Identity
4.	Beliefs about one's ability to successfully carry out a behaviour	Beliefs about Capabilities
5.	Confidence that things will happen for the best or that desired goals will be attained	Optimism
6.	Beliefs about the consequences of a behaviour (i.e. perceptions about what will be achieved and/or lost by undertaking a behaviour, as well as the probability that a behaviour will lead to a specific outcome)	Beliefs about Consequences
7.	Processes by which the frequency or probability of a response is increased through a dependent relationship or contingency with a stimulus or circumstance	Reinforcement
8.	A conscious decision to perform a behaviour or a resolve to act in a certain way	Intentions
9.	Mental representations of outcomes or end states that an individual wants to achieve	Goals
10.	Ability to retain information, focus on aspects of the environment and choose between two or more alternatives	Memory, Attention and Decision Processes

Susan Michie, Marie Johnston, Alex Rothman, Mike Kelly, Marijn de Bruin
Email bct.theory@ucl.ac.uk

11.	Aspects of a person's situation or environment that discourage or encourage the behaviour	Environmental Context and Resources
12.	Those interpersonal processes that can cause oneself to change one's thoughts, feelings or behaviours.	Social Influences
13.	A complex reaction pattern involving experiential, behavioural, and physiological elements	Emotion
14.	Behavioural, cognitive and/or emotional skills for managing or changing behaviour	Behavioural Regulation
15.	The attitudes held and behaviours exhibited by other people within a social group	Norms
16.	One's ***perceptions*** of what most other people within a social group believe and do	Subjective Norms
17.	The general evaluations of the behaviour on a scale ranging from negative to positive	Attitude towards the behaviour
18.	Processes relating to the impetus that gives purpose or direction to behaviour and operates at a conscious or unconscious level	Motivation
19.	One's conception and evaluation of oneself, including psychological and physical characteristics, qualities and skills	Self-image
20.	Deficit of something required for survival, well-being or personal fulfilment	Needs
21.	Moral, social or aesthetic principles accepted by an individual or society as a guide to what is good, desirable or important	Values
22.	Processes through which current behaviour is compared against a particular standard	Feedback Processes
23.	A process by which thoughts, feelings and motivational states observed in others are internalised and replicated without the need for conscious awareness	Social Learning / Imitation

Susan Michie, Marie Johnston, Alex Rothman, Mike Kelly, Marijn de Bruin
Email bct.theory@ucl.ac.uk

24.	Processes by which behaviour is triggered from either the external environment, the performance of another behaviour, or from ideas appearing in consciousness	Behavioural Cueing
25.	Evaluations of an object, person, group, issue or concept on a scale ranging from negative to positive	General Attitudes/ Beliefs
26.	Perceptions of the likelihood that one is vulnerable to a threat	Perceived susceptibility/ vulnerability

Susan Michie, Marie Johnston, Alex Rothman, Mike Kelly, Marijn de Bruin
Email bct.theory@ucl.ac.uk

Appendix C: Methodology for Literature Synthesis and Expert Consensus Studies

Literature Synthesis Study Method:

- BCT-mechanism of action links, as hypothesised by authors, were extracted from 277 behaviour change intervention papers.
- In order for a link to be extracted, the author(s) must have explicitly hypothesised that a particular BCT would have its effect on behaviour through a particular mechanism of action.
- BCTs were extracted using the coding guidelines for BCT Taxonomy v1. Mechanisms of action were extracted using the intervention authors' definitions, and then categorised into one of the 26 mechanisms of action listed in Appendix B.
- The 'frequency' of a given BCT-mechanism of action link refers to the number of papers that reported that link (out of a total 277).
- In order to determine whether a particular link occurred more frequently than would be expected compared to chance, a series of binomial tests were conducted on the data. The expected frequency used for comparison in the binomial test was calculated for each individual link, and was the product of the probability a given BCT was coded in a paper × the probability a given mechanism was coded in a paper.
- The p value in the tables below represents an index of the likelihood that an observed frequency of a BCT-mechanism of action link occurred more often than would be expected if that link was a random pairing of a BCT and a mechanism of action (i.e. lower p values denote higher likelihood).
- We are considering there to be 'evidence' for a specific link where $p < .05$.

Expert Consensus Study Method:

- 105 behaviour change experts were asked to consider links between BCTs and mechanisms of action in an online expert consensus exercise. Experts were divided into 5 groups, with 21 experts per group, and each group rated a sub-sample of BCTs. Thus, for the links below, n = 21 unless otherwise specified.
- Experts were asked the question 'When [BCT X] works, does it work through changing [Mechanism of Action Y]?'
- The response options were
    1. 'Definitely Yes' (i.e. When this BCT is effective in changing behaviour, it does so by changing this mechanism of action);
    2. 'Definitely No' (i.e. When this BCT is effective in changing behaviour, it does not do so by changing this mechanism of action);
    3. 'Possibly' (i.e. When this BCT is effective in changing behaviour, it is possible that it does so by changing this mechanism of action) and;
    4. 'Don't Know / Not Possible to Say' (i.e. It is not possible to say (e.g. not enough information to decide) and/or you are very uncertain about whether or not the BCT changes behaviour by changing this mechanism of action).
- We are considering there to be 'evidence' for a link where ≥80% of experts answered 'Definitely Yes'. All tables below are ordered according to the percentage of experts who answered 'Definitely Yes'.

Susan Michie, Marie Johnston, Alex Rothman, Mike Kelly, Marijn de Bruin
Email bct.theory@ucl.ac.uk

Appendix D: Data from Literature Synthesis and Expert Consensus

This appendix contains the links for which there are inconsistencies or uncertainties across literature synthesis and expert consensus (i.e. the links you will be asked to consider in the consensus exercise). Note that all data is confidential and should not be reproduced without permission.

Table 1: Evidence indicates that there was a link in the literature synthesis and 'definitely no' link in expert consensus

		Literature Synthesis Study		Expert Consensus Study			
**Behaviour Change Technique**	**Mechanism of Action**	Frequency (# papers)	*p* value	% Experts (Yes)	% Experts (Possibly)	% Experts (Don't Know)	% Experts (No)
2.1 Monitoring of Behaviour by Others without Feedback	Needs	1	.018	0	11	0	89
2.7 Feedback on Outcomes of Behaviour	Subjective Norms	5	.018	0	10	10	80
5.6 Information about Emotional Consequences	Social Influences	2	.022	5	10	5	80
12.3 Avoidance / Reducing Exposure to Cues for the Behaviour	Needs	1	.026	5	5	0	90

Susan Michie, Marie Johnston, Alex Rothman, Mike Kelly, Marijn de Bruin
Email bct.theory@ucl.ac.uk

Table 2: Evidence indicates that there was a link in the literature synthesis and disagreement about the link in expert consensus

		**Literature Synthesis Study**		**Expert Consensus Study**			
**Behaviour Change Technique**	**Mechanism of Action**	Frequency (# papers)	*p* value	% Experts (Yes)	% Experts (Possibly)	% Experts (Don't Know)	% Experts (No)
8.2 Behaviour Substitution	Behavioural Regulation	5	0.016	79	5	0	16
5.3 Information about Social and Environmental Consequences	Attitude towards the Behaviour	16	<0.001	76	5	9.5	9.5
7.1 Prompts/Cues	Memory, Attention, and Decision Processes	8	<0.001	76	24	0	0
4.1 Instruction on how to Perform the Behaviour	Knowledge	17	0.011	74	18	1	7
8.4 Habit Reversal	Behavioural Regulation	4	0.006	74	21	0	5
8.4 Habit Reversal	Behavioural Cueing	2	0.034	74	26	0	0
8.6 Generalisation of the Target Behaviour	Skills	2	0.044	74	10	0	16
2.4 Self-Monitoring of Outcomes of Behaviour	Behavioural Regulation	5	0.024	71	24	0	5
1.8 Behavioural Contract	Goals	4	0.002	70	0	20	10
2.3 Self-Monitoring of Behaviour	Behavioural Regulation	18	<0.001	70	15	0	15
5.6 Information about Emotional Consequences	Emotion	2	0.02	70	10	5	15
5.1 Information about Health Consequences	Attitude towards the Behaviour	19	<0.001	68	32	5	11
9.1 Credible Source	General Attitudes / Beliefs	2	0.006	68	26	0	6

Susan Michie, Marie Johnston, Alex Rothman, Mike Kelly, Marijn de Bruin
Email bct.theory@ucl.ac.uk

5.3 Information about Social and Environmental Consequences	Knowledge	13	0.001	67	24	0	9
12.2 Restructuring the Social Environment	Social Influences	6	<0.001	65	5	5	25
5.1 Information about Health Consequences	Intention	28	0.005	63	26	0	11
11.2 Reduce Negative Emotions	Beliefs about Capabilities	12	0.039	62	14	5	19
5.6 Information about Emotional Consequences	Attitude towards the Behaviour	5	0.005	60	35	0	5
1.4 Action Planning	Behavioural Regulation	14	0.002	58	16	0	26
2.1 Monitoring of Behaviour by Others without Feedback	Social Influences	2	0.034	58	37	0	5
6.1 Demonstration of the Behaviour	Beliefs about Capabilities	60	0.001	58	37	5	0
8.4 Habit Reversal	Memory, Attention, and Decision Processes	2	0.034	58	16	5	21
10.1 Material Incentive (behaviour)	Attitude towards the behaviour	1	0.047	58	21	0	21
5.2 Salience of Consequences	Attitude towards the Behaviour	4	0.029	55	30	0	15
6.3 Information about Others' Approval	Intention	12	0.038	53	32	5	10
8.3 Habit Formation	Behavioural Regulation	3	0.022	45	20	0	35
2.2 Feedback on Behaviour	Knowledge	13	0.010	42	32	0	26
15.4 Self-talk	Beliefs about Capabilities	8	0.045	42	47	0	11

1.6 Discrepancy between current behaviour and goal	Behavioural Regulation	3	0.017	33	24	0	43
1.1 Goal Setting (behaviour)	Behavioural Regulation	15	0.004	35	35	0	30
6.1 Demonstration of the Behaviour	Skills	17	0.015	26	37	5	32
1.9 Commitment	Values	1	0.049	21	42	5	32
9.1 Credible Source	Social / Professional Role and Identity	4	0.021	11	58	0	31
3.1 Social Support (Unspecified)	Social / Professional Role and Identity	5	0.035	10	28	4	58
2.2 Feedback on Behaviour	Subjective Norms	19	<0.001	5	16	11	68
9.2 Pros and Cons	Feedback Processes	3	0.005	0	29	0	71
5.5 Anticipated Regret	Social Influences	2	0.002	N/A	N/A	N/A	N/A
7.8 Associative Learning	Reinforcement	1	0.049	N/A	N/A	N/A	N/A
10.7 Self-Incentive	Motivation	1	0.034	N/A	N/A	N/A	N/A
13.5 Identity Associated with Changed Behaviour	Values	1	0.016	N/A	N/A	N/A	N/A
15.2 Mental Rehearsal of Successful Performance	Environmental Context / Resources	3	0.001	N/A	N/A	N/A	N/A
15.2 Mental Rehearsal of Successful Performance	Values	1	0.034	N/A	N/A	N/A	N/A
15.3 Focus on Past Success	Beliefs about Capabilities	23	<0.001	N/A	N/A	N/A	N/A

Note: Cells display N/A for the literature synthesis where the BCT was not coded in any of the 277 interventions. Cells display N/A for the expert consensus where the BCT was not considered by experts.

Susan Michie, Marie Johnston, Alex Rothman, Mike Kelly, Marijn de Bruin
Email bct.theory@ucl.ac.uk

Table 3: No evidence to indicate that there was a link in the literature synthesis, evidence indicates there was 'definitely' a link in expert consensus

		Literature Synthesis Study		Expert Consensus Study			
**Behaviour Change Technique**	**Mechanism of Action**	Frequency (# papers)	*p* value	% Experts (Yes)	% Experts (Possibly)	% Experts (Don't Know)	% Experts (No)
1.2 Problem Solving	Behavioural Regulation	13	.16	100	0	0	0
1.6 Discrepancy between Current Behaviour and Goal	Feedback Processes	1	.07	100	0	0	0
11.3 Conserving Mental Resources	Memory, Attention, Decision Processes	1	.06	100	0	0	0
10.8 Incentive (Outcome)	Motivation	N/A	N/A	100	0	0	0
1.1 Goal Setting (behaviour)	Intention	17	.34	95	5	0	0
1.1 Goal Setting (behaviour)	Goals	4	.23	95	0	5	0
1.5 Review Behaviour Goals	Feedback Processes	0	1	95	5	0	0
2.2 Feedback on Behaviour	Feedback Processes	3	.07	95	5	0	0
10.1 Material Incentive (Behaviour)	Reinforcement	0	1	95	5	0	0
10.4 Social Reward	Social Influences	2	.55	95	5	0	0
11.2 Reduce Negative Emotions	Emotion	1	.21	95	0	5	0
12.3 Avoidance/Reducing Exposure to Cues	Environmental Context & Resources	1	.24	95	5	0	0
12.3 Avoidance/Reducing Exposure to Cues	Behavioural Cueing	0	1	95	5	0	0

Susan Michie, Marie Johnston, Alex Rothman, Mike Kelly, Marijn de Bruin
Email bct.theory@ucl.ac.uk

12.5 Adding Objects to the Environment	Behavioural Cueing	2	.11	95	5	0	0
14.10 Remove Punishment	Reinforcement	0	1	95	5	0	0
10.8 Incentive (Outcome)	Intention	N/A	N/A	95	0	0	5
1.5 Review Behaviour Goals	Goals	2	.07	90	0	0	10
6.2 Social Comparison	Norms	0	1	90	5	0	5
6.2 Social Comparison	Feedback Processes	2	.20	90	5	0	5
7.1 Prompts/Cues	Environmental Context & Resources	5	.05	90	5	0	5
11.2 Reduce Negative Emotions	Behavioural Regulation	0	1	90	5	5	0
7.5 Remove Aversive Stimulus	Environmental Context & Resources	N/A	N/A	90	5	5	0
10.8 Incentive (Outcome)	Beliefs about Consequences	N/A	N/A	90	5	0	5
10.8 Incentive (Outcome)	Reinforcement	N/A	N/A	90	0	5	5
10.10 Reward (Outcome)	Reinforcement	N/A	N/A	90	5	5	0
10.10 Reward (Outcome)	Motivation	N/A	N/A	90	10	0	0
6.3 Information about Other's Approval	Norms	0	1	90	5	5	0
10.2 Material Reward (Behaviour)	Reinforcement	0	1	90	0	5	5
2.6 Biofeedback	Feedback Processes	0	1	89	11	0	0
1.3 Goal Setting (Outcome)	Intention	5	.27	86	9	0	5
4.2 Information about Antecedents	Knowledge	3	.05	86	14	0	0

Susan Michie, Marie Johnston, Alex Rothman, Mike Kelly, Marijn de Bruin
Email bct.theory@ucl.ac.uk

5.3 Information about Social and Environmental Consequences	Motivation	5	.24	86	5	0	9
9.3 Comparative Imagining of Future Outcomes	Motivation	1	.26	86	9	0	5
10.10 Reward (Outcome)	Beliefs about Consequences	N/A	N/A	85	5	0	10
5.2 Salience of Consequences	Beliefs about Consequences	2	.46	85	10	0	5
16.3 Vicarious Consequences	Social Learning/Imitation	1	.10	85	5	5	5
1.9 Commitment	Intention	5	.13	84	16	0	0
1.9 Commitment	Motivation	1	.53	84	16	0	0
3.3 Social Support (Emotional)	Emotion	1	.16	84	16	0	0
6.3 Information about Others' Approval	Social Influences	4	.19	84	16	0	0
8.4 Habit Reversal	Skills	1	.58	84	5	0	11
10.1 Material Incentive (Behaviour)	Motivation	0	1	84	16	0	0
10.3 Non-specific Reward	Motivation	1	.29	84	11	0	5
4.2 Information about Antecedents	Behavioural Cueing	0	1	81	5	0	14
8.7 Graded Tasks	Skills	4	.42	81	14	0	5
9.3 Comparative Imagining of Future Outcomes	Intention	1	.66	81	10	0	9
2.4 Self-Monitoring of Outcomes of Behaviour	Feedback Processes	1	.20	81	9	5	5

Susan Michie, Marie Johnston, Alex Rothman, Mike Kelly, Marijn de Bruin
Email bct.theory@ucl.ac.uk

10.4 Social Reward	Motivation	3	.20	81	14	0	5
1.1 Goal Setting (behaviour)	Motivation	3	.80	80	20	0	0
1.8 Behavioural Contract	Intention	4	.44	80	5	5	10
2.3 Self-Monitoring of Behaviour	Feedback Processes	2	.16	80	15	0	5
5.4 Monitoring of Emotional Consequences	Emotion	0	1	80	15	0	5
12.2 Restructuring the Social Environment	Behavioural Cueing	0	1	80	10	0	10
16.3 Vicarious Consequences	Norms	0	1	80	5	10	5

Note: Cells display N/A for the literature synthesis where the BCT was not coded in any of the 277 interventions. Cells display N/A for the expert consensus where the BCT was not considered by experts.

Table 4: Marginal evidence indicates that there was a link in the literature synthesis OR expert consensus (i.e. *p* ≥ .05 and ≤ .1 OR 70 – 79% experts answered 'Definitely Yes').

		**Literature Synthesis Study**		**Expert Consensus Study**			
**Behaviour Change Technique**	**Mechanism of Action**	Frequency (# papers)	*p* value	% Experts (Yes)	% Experts (Possibly)	% Experts (Don't Know)	% Experts (No)
1.7 Review Outcome Goals	Intention	1	0.62	79	16	0	5
1.7 Review Outcome Goals	Feedback Processes	0	1	79	21	0	0
1.9 Commitment	Goals	1	0.35	79	21	0	0
2.6 Biofeedback	Knowledge	0	1	79	16	0	5
3.3 Social Support (emotional)	Social Influences	2	0.17	79	21	0	0
4.1 Instruction on how to Perform the Behaviour	Beliefs about Capabilities	62	0.06	79	17	0	4
8.4 Habit Reversal	Intention	1	0.79	79	16	0	5
8.2 Behaviour Substitution	Behavioural Cueing	2	0.0784	79	21	0	0
9.1 Credible Source	Attitude towards the Behaviour	7	0.08	79	21	0	0
10.1 Material Incentive (behaviour)	Beliefs about Consequences	0	1	79	11	0	11
10.6 Non-specific Incentive	Reinforcement	N/A	N/A	79	5	0	16
14.10 Remove Punishment	Beliefs about Consequences	0	1	79	10.5	0	10.5
15.4 Self-talk	Motivation	2	0.08	79	16	0	5

1.3 Goal Setting (outcome)	Motivation	3	0.08	76	19	0	5
2.4 Self-Monitoring of Outcomes of Behaviour	Beliefs about Capabilities	7	0.73	76	24	0	0
2.4 Self-Monitoring of Outcomes of Behaviour	Motivation	0	1	76	5	9.5	9.5
3.2 Social Support (practical)	Behavioural Cueing	0	1	76	19	0	5
4.2 Information about Antecedents	Behavioural Regulation	2	0.23	76	10	0	14
8.7 Graded Tasks	Motivation	1	0.82	76	19	0	5
9.2 Pros and Cons	General Attitudes & Beliefs	0	1	76	24	0	0
11.3 Conserving Mental Resources	Behavioural Regulation	1	0.15	76	14	0	10
15.1 Verbal Persuasion about Capability	Social Influences	1	0.79	76	10	0	14
15.1 Verbal Persuasion about Capability	Motivation	1	0.73	76	14	0	10
16.3 Vicarious Consequences	Subjective Norms	1	0.58	75	20	0	5
1.4 Action Planning	Goals	3	0.32	74	16	0	10
1.4 Action Planning	Behavioural Cueing	4	0.10	74	16	0	10
1.7 Review Outcome Goals	Motivation	0	1	74	16	0	10
2.1 Monitoring of Behaviour by Others without Feedback	Behavioural Cueing	0	1	74	26	0	0
2.6 Biofeedback	Reinforcement	0	1	74	21	0	5
2.6 Biofeedback	Motivation	1	0.24	74	21	0	5
8.6 Generalisation of the Target Behaviour	Beliefs about Capabilities	0	1	74	21	0	5

9.1 Credible Source	Social Influences	5	0.22	74	26	0	0
10.1 Material Incentive (behaviour)	Intention	0	1	74	26	0	0
10.6 Non-specific Incentive	Intention	N/A	N/A	74	16	0	10
10.6 Non-specific Incentive	Motivation	N/A	N/A	74	16	0	10
14.10 Remove Punishment	Motivation	0	1	74	21	0	5
6.2 Social Comparison	Values	0	1	71	19	0	10
9.2 Pros and Cons	Memory, Attention, & Decision Processes	0	1	71	14	5	10
9.3 Comparative Imagining of Future Outcomes	Attitude towards the Behaviour	0	1	71	14	5	10
10.8 Incentive (outcome)	Goals	N/A	N/A	71	14.5	0	14.5
12.6 Body Changes	Self-Image	0	1	71	19	0	10
13.2 Framing / Reframing	Beliefs about Consequences	5	0.28	71	24	5	0
13.2 Framing / Reframing	Motivation	3	0.29	71	29	0	0
15.1 Verbal Persuasion about Capability	Self-Image	0	1	71	14.5	0	14.5
1.5 Review Behaviour Goals	Intention	5	0.11	70	15	0	15
1.5 Review Behaviour Goals	Behavioural Regulation	2	0.30	70	15	0	15
2.7 Feedback on Outcomes of Behaviour	Beliefs about Capabilities	7	0.78	70	20	0	10
2.7 Feedback on Outcomes of Behaviour	Reinforcement	1	0.33	70	15	5	10
5.2 Salience of Consequences	Perceived Susceptibility/Vulnerability	1	0.17	70	25	0	5

8.3 Habit Formation	Memory, Attention, & Decision Processes	1	0.21	70	10	5	15
8.3 Habit Formation	Environmental Context & Resources	0	1	70	10	5	15
16.3 Vicarious Consequences	Social Influences	0	1	70	25	0	5
2.2 Feedback on Behaviour	Motivation	8	0.06	68	32	0	0
11.3 Conserving Mental Resources	Environment	1	0.08	67	28	0	5
9.1 Credible Source	Social Learning/Imitation	2	0.07	63	21	0	16
2.2 Feedback on Behaviour	Reinforcement	4	0.07	58	26	0	16
13.2 Framing / Reframing	General Attitude & Beliefs	1	0.08	52	38	5	5
2.7 Feedback on Outcomes of Behaviour	Motivation	3	0.10	50	35	0	15
1.2 Problem Solving	Skills	18	0.05	43	24	5	28
1.2 Problem Solving	Environment	9	0.05	43	24	0	33
10.4 Social Reward	Beliefs about Capabilities	18	0.08	43	29	0	28
1.1 Goal Setting (behaviour)	Beliefs about Capabilities	44	0.07	35	30	0	35
8.3 Habit Formation	Motivation	2	0.06	35	20	0	45
1.8 Behavioural Contract	Behavioural Regulation	3	0.07	25	15	5	55
2.1 Monitoring of Behaviour by Others without Feedback	Reinforcement	1	0.08	16	31.5	10.5	42
10.2 Material Reward (behaviour)	Goals	1	0.09	16	58	10	16
12.6 Body Changes	Emotion	1	0.07	14	29	9	48
1.7 Review Outcome Goals	Optimism	1	0.06	11	53	5	31
1.9 Commitment	Memory, Attention, & Decision Processes	2	0.09	11	26	16	47

2.6 Biofeedback	Beliefs about Capabilities	5	0.07	11	42	0	47
5.2 Salience of Consequences	Subjective Norms	8	0.09	10	25	5	60
8.1 Behavioural Practice / Rehearsal	Behavioural Cueing	5	0.05	10	33	0	57
4.2 Information about Antecedents	Skills	3	0.10	9	5	0	86
10.3 Non-specific Reward	Self-Image	1	0.07	5	0	0	95
13.2 Framing / Reframing	Self-Image	2	0.06	5	57	5	33
5.5 Anticipated Regret	Beliefs about Consequences	2	0.06	N/A	N/A	N/A	N/A
7.8 Associative Learning	Behavioural Cueing	1	0.07	N/A	N/A	N/A	N/A
7.8 Associative Learning	Memory, Attention, & Decision Processes	1	0.08	N/A	N/A	N/A	N/A
13.3 Incompatible Beliefs	Attitude towards the Behaviour	1	0.09	N/A	N/A	N/A	N/A
13.4 Valued Self-Identity	Intention	2	0.05	N/A	N/A	N/A	N/A
13.4 Valued Self-Identity	Motivation	1	0.10	N/A	N/A	N/A	N/A
13.5 Identity Associated with Changed Behaviour	Social/Professional Role & Identity	1	0.09	N/A	N/A	N/A	N/A
14.1 Behaviour Cost	Behavioural Regulation	1	0.05	N/A	N/A	N/A	N/A
15.2 Mental Rehearsal of Successful Performance	Motivation	2	0.09	N/A	N/A	N/A	N/A

Note: Cells display N/A for the literature synthesis where the BCT was not coded in any of the 277 interventions. Cells display N/A for the expert consensus where the BCT was not considered by experts.

Susan Michie, Marie Johnston, Alex Rothman, Mike Kelly, Marijn de Bruin
Email bct.theory@ucl.ac.uk

Appendix E: Examples of links agreed across the literature synthesis and expert consensus

The following are links for which there is agreement across literature synthesis and expert consensus. You will not be asked to consider these links and they are presented here for information only. Note that all data is confidential and should not be reproduced without permission.

Table 5: Example of links agreed across both studies (i.e. evidence indicates a link in literature synthesis and expert consensus)

		**Literature Synthesis Study**		**Expert Consensus Study**			
**Behaviour Change Technique**	**Mechanism of Action**	Frequency (# papers)	*p* value	% Experts (Yes)	% Experts (Possibly)	% Experts (Don't Know)	% Experts (No)
1.2 Problem Solving	Beliefs about Capabilities	65	0.017	95	5	0	0
7.1 Prompts/Cues	Behavioural Cueing	6	0.002	100	0	0	0
15.1 Verbal Persuasion about Capability	Beliefs about Capabilities	27	<0.001	100	0	0	0

Table 6: Example of non-links agreed across both studies (i.e. no evidence indicates a link in the literature synthesis and evidence indicates 'definitely no' link in expert consensus)

		**Literature Synthesis Study**		**Expert Consensus Study**			
**Behaviour Change Technique**	**Mechanism of Action**	Frequency (# papers)	*p* value	% Experts (Yes)	% Experts (Possibly)	% Experts (Don't Know)	% Experts (No)
1.4 Action Planning	Norms	0	1	5	0	0	95
1.9 Commitment	Skills	0	1	5	11	0	84
2.3 Self-Monitoring of Behaviour	Social Learning/Imitation	0	1	0	5	0	95

Susan Michie, Marie Johnston, Alex Rothman, Mike Kelly, Marijn de Bruin
Email bct.theory@ucl.ac.uk

Appendix F: Project Summary

BACKGROUND: The effectiveness of behaviour change interventions depends on good understanding of the links between behaviour change techniques (BCTs) and their mechanisms of action (i.e. the processes through which they affect behaviour). Considerable progress has been made in developing a method for specifying BCTs. The aim of this research is to develop and test a methodology for linking BCTs to their hypothesised mechanisms of action.

METHODS/DESIGN:

Study 1: To identify and evaluate evidence of links between BCTs (from the 93-item BCT taxonomy, BCTTv1) and their hypothesised mechanisms of action, data will be extracted from published intervention papers. The frequency with which each BCT is linked by authors to each mechanism of action in descriptions of behavioural interventions will be reported.

Study 2: Behaviour change experts will identify links between BCTs and mechanisms of action in a formal consensus development study. They will be asked about 26 mechanisms, 12 frequently occurring in theories of behaviour change and 14 domains from the Theoretical Domains Framework. The data from Studies 1 and 2 will generate matrices of hypothesised links between BCTs and mechanisms.

Study 3: Agreement between the matrices from Studies 1 and 2 will be evaluated. A new group of experts will be consulted to discuss and resolve discrepancies. An integrated matrix of BCT-mechanism links, annotated to indicate strength of evidence, will be generated.

Study 4: To identify whether groups of co-occurring BCTs can be linked to theories, groups of two or more BCTs that work together will be identified from the Study 1 literature synthesis. A consensus exercise will be used to rate strength of links between BCT groups and theories, generating a matrix of links between BCT groups and theories.

Discussion: The development of a formal methodology for linking BCTs to mechanisms of action has the potential to make a substantial contribution to the development and evaluation of behaviour change interventions. This research is a step towards developing an ‘ontology’ of behaviour change that specifies the relations between BCTs, theoretical mechanisms, modes of delivery, populations, settings and types of behaviour.

Susan Michie, Marie Johnston, Alex Rothman, Mike Kelly, Marijn de Bruin
Email bct.theory@ucl.ac.uk
